# Supplementary material for: Genetic Diversity and Trends of Ancestral and New Inbreeding in German Sheep Breeds by Pedigree Data
Source: Animals (Basel). 2023 Feb 10;13(4):623. doi: 10.3390/ani13040623 (PMC9951766; doi:10.3390/ani13040623)
Supplement: Supplementary file 1 [file animals-13-00623-s001.zip › Figure S1-Number.pdf]

Figure S1. Number of animals in the reference population (N_{Ref}) and the number of equivalent generations (EqG) for the birth years 2010 to 2020 for each of the 35 sheep breeds in Germany.

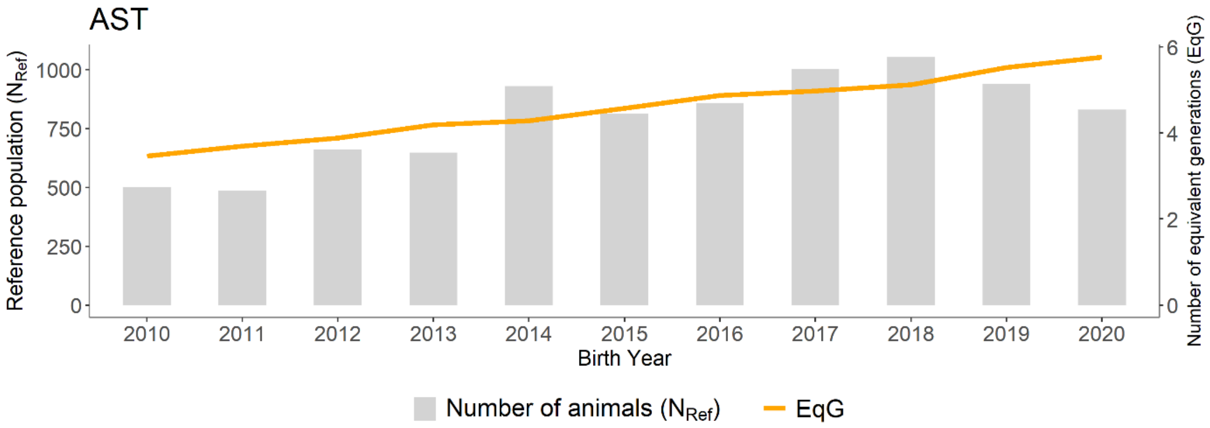

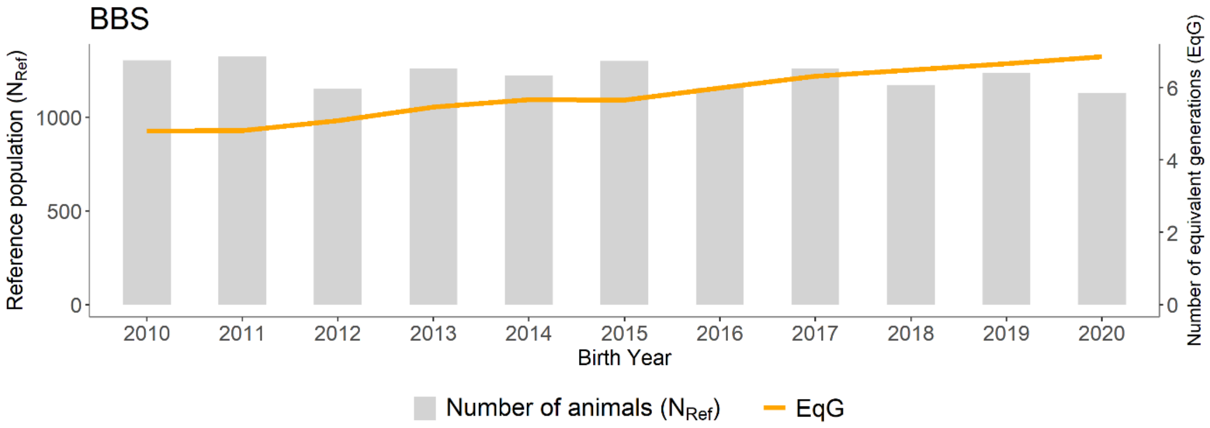

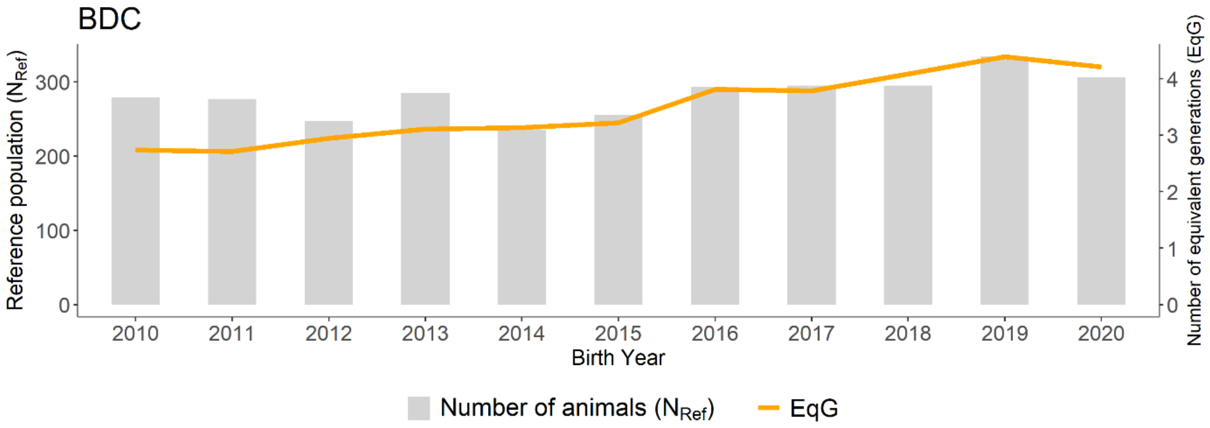

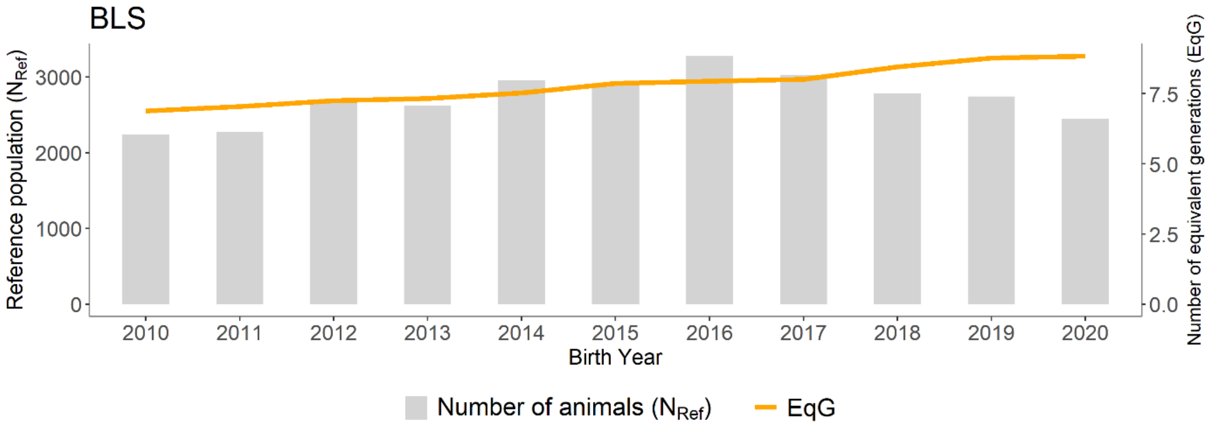
BLS
Reference population (N_{Ref})
0
1000
2000
3000
Number of equivalent generations (EqG)
0.0
2.5
5.0
7.5
2010
2011
2012
2013
2014
2015
2016
2017
2018
2019
2020
Birth Year
Number of animals (N_{Ref})
EqG

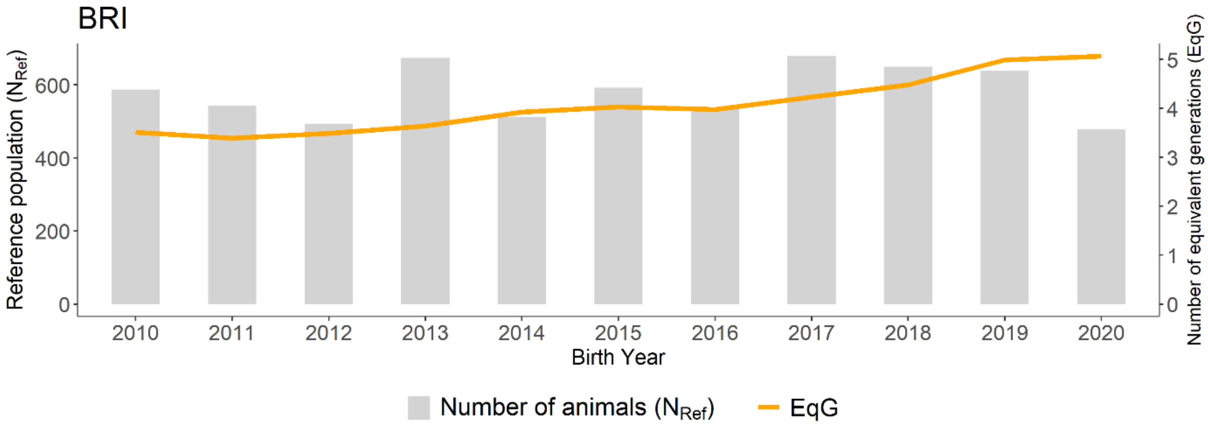
BRI
Reference population (N_{Ref})
0
200
400
600
Number of equivalent generations (EqG)
0
1
2
3
4
5
2010
2011
2012
2013
2014
2015
2016
2017
2018
2019
2020
Birth Year
Number of animals (N_{Ref})
EqG

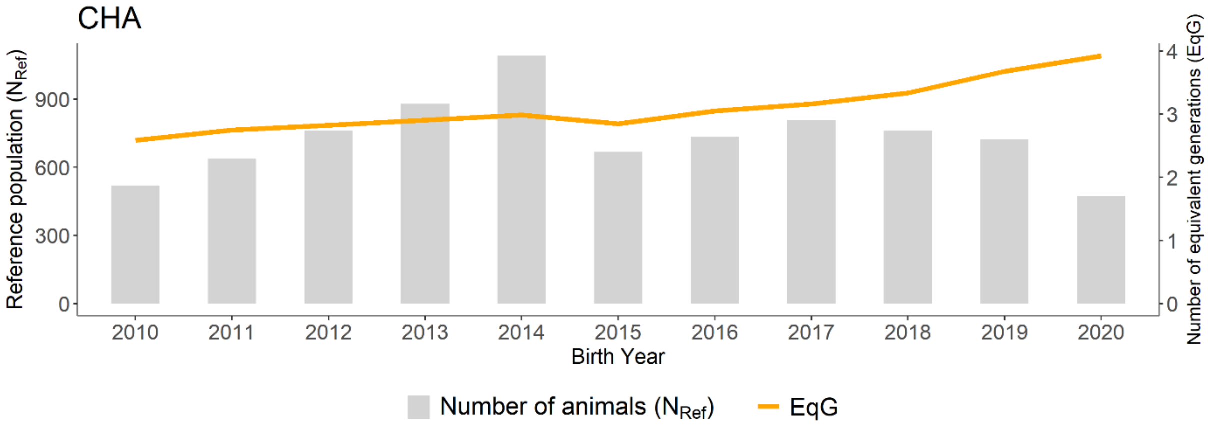
CHA
Reference population (N_{Ref})
0
300
600
900
Number of equivalent generations (EqG)
0
1
2
3
4
2010
2011
2012
2013
2014
2015
2016
2017
2018
2019
2020
Birth Year
Number of animals (N_{Ref})
EqG

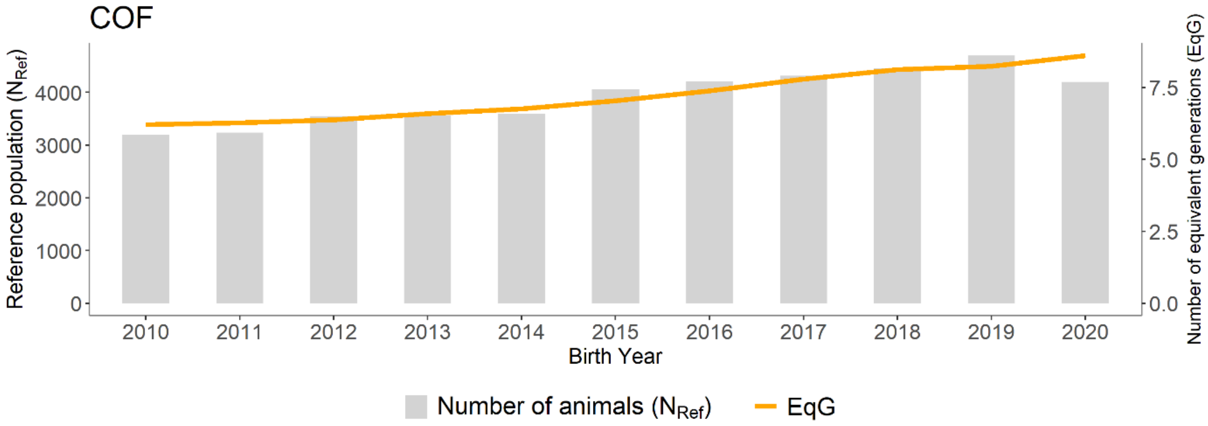
COF
Reference population (N_{Ref})
0
1000
2000
3000
4000
Number of equivalent generations (EqG)
0.0
2.5
5.0
7.5
2010
2011
2012
2013
2014
2015
2016
2017
2018
2019
2020
Birth Year
Number of animals (N_{Ref})
EqG

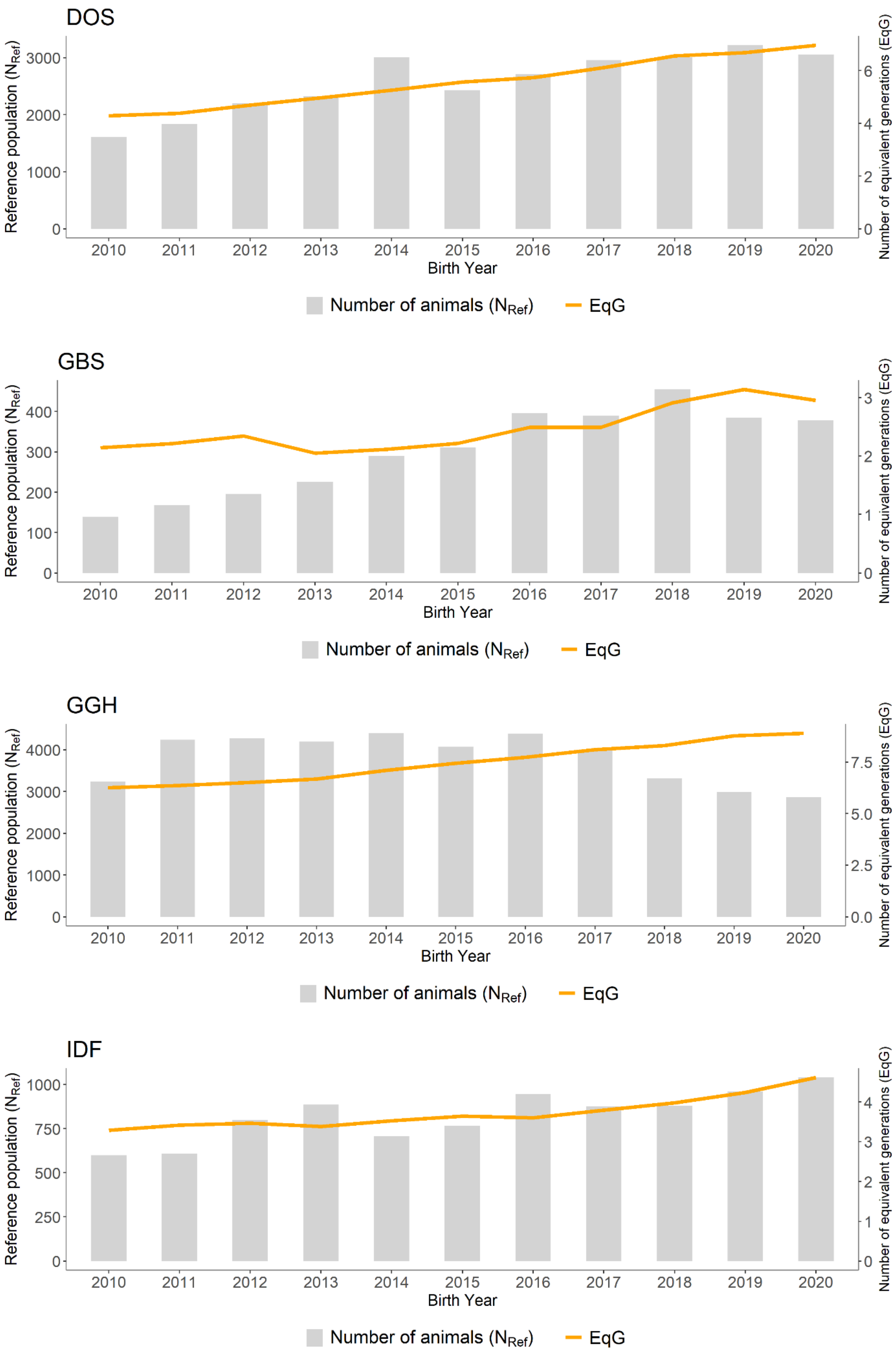
DOS
Reference population (N_{Ref})
Number of equivalent generations (EqG)
Birth Year
2010 2011 2012 2013 2014 2015 2016 2017 2018 2019 2020
Number of animals (N_{Ref})
EqG
GBS
Reference population (N_{Ref})
Number of equivalent generations (EqG)
Birth Year
Number of animals (N_{Ref})
EqG
GGH
Reference population (N_{Ref})
Number of equivalent generations (EqG)
Birth Year
Number of animals (N_{Ref})
EqG
IDF
Reference population (N_{Ref})
Number of equivalent generations (EqG)
Birth Year
Number of animals (N_{Ref})
EqG

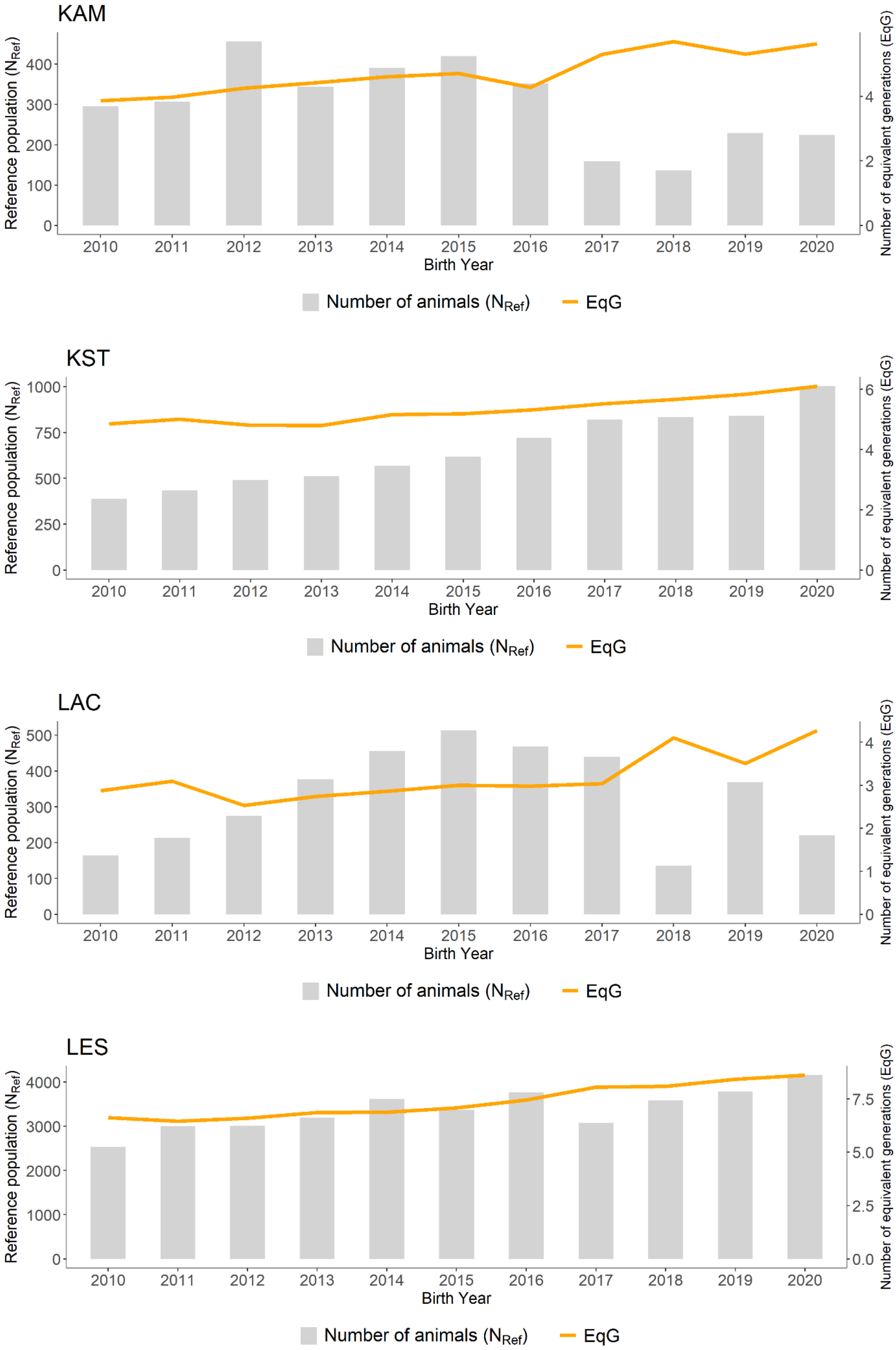
KAM
Reference population (N_{Ref})
Number of equivalent generations (EqG)
0
100
200
300
400
0
2
4
2010
2011
2012
2013
2014
2015
2016
2017
2018
2019
2020
Birth Year
Number of animals (N_{Ref})
EqG
KST
Reference population (N_{Ref})
Number of equivalent generations (EqG)
0
250
500
750
1000
0
2
4
6
2010
2011
2012
2013
2014
2015
2016
2017
2018
2019
2020
Birth Year
Number of animals (N_{Ref})
EqG
LAC
Reference population (N_{Ref})
Number of equivalent generations (EqG)
0
100
200
300
400
500
0
1
2
3
4
2010
2011
2012
2013
2014
2015
2016
2017
2018
2019
2020
Birth Year
Number of animals (N_{Ref})
EqG
LES
Reference population (N_{Ref})
Number of equivalent generations (EqG)
0
1000
2000
3000
4000
0.0
2.5
5.0
7.5
2010
2011
2012
2013
2014
2015
2016
2017
2018
2019
2020
Birth Year
Number of animals (N_{Ref})
EqG

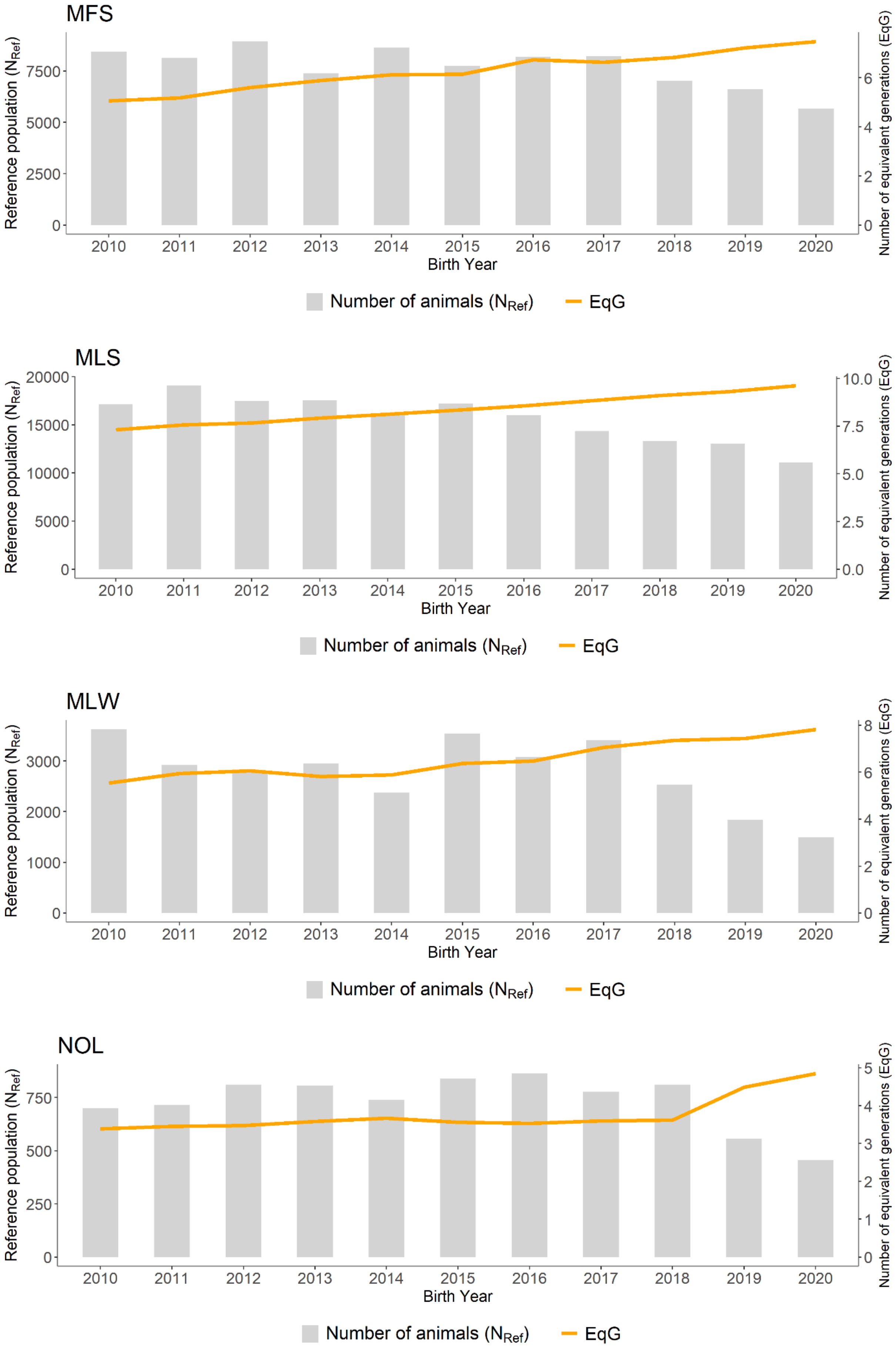
MFS
Reference population (N_{Ref})
0
2500
5000
7500
Number of equivalent generations (EqG)
0
2
4
6
2010
2011
2012
2013
2014
2015
2016
2017
2018
2019
2020
Birth Year
Number of animals (N_{Ref})
EqG
MLS
Reference population (N_{Ref})
0
5000
10000
15000
20000
Number of equivalent generations (EqG)
0.0
2.5
5.0
7.5
10.0
2010
2011
2012
2013
2014
2015
2016
2017
2018
2019
2020
Birth Year
Number of animals (N_{Ref})
EqG
MLW
Reference population (N_{Ref})
0
1000
2000
3000
Number of equivalent generations (EqG)
0
2
4
6
8
2010
2011
2012
2013
2014
2015
2016
2017
2018
2019
2020
Birth Year
Number of animals (N_{Ref})
EqG
NOL
Reference population (N_{Ref})
0
250
500
750
Number of equivalent generations (EqG)
0
1
2
3
4
5
2010
2011
2012
2013
2014
2015
2016
2017
2018
2019
2020
Birth Year
Number of animals (N_{Ref})
EqG

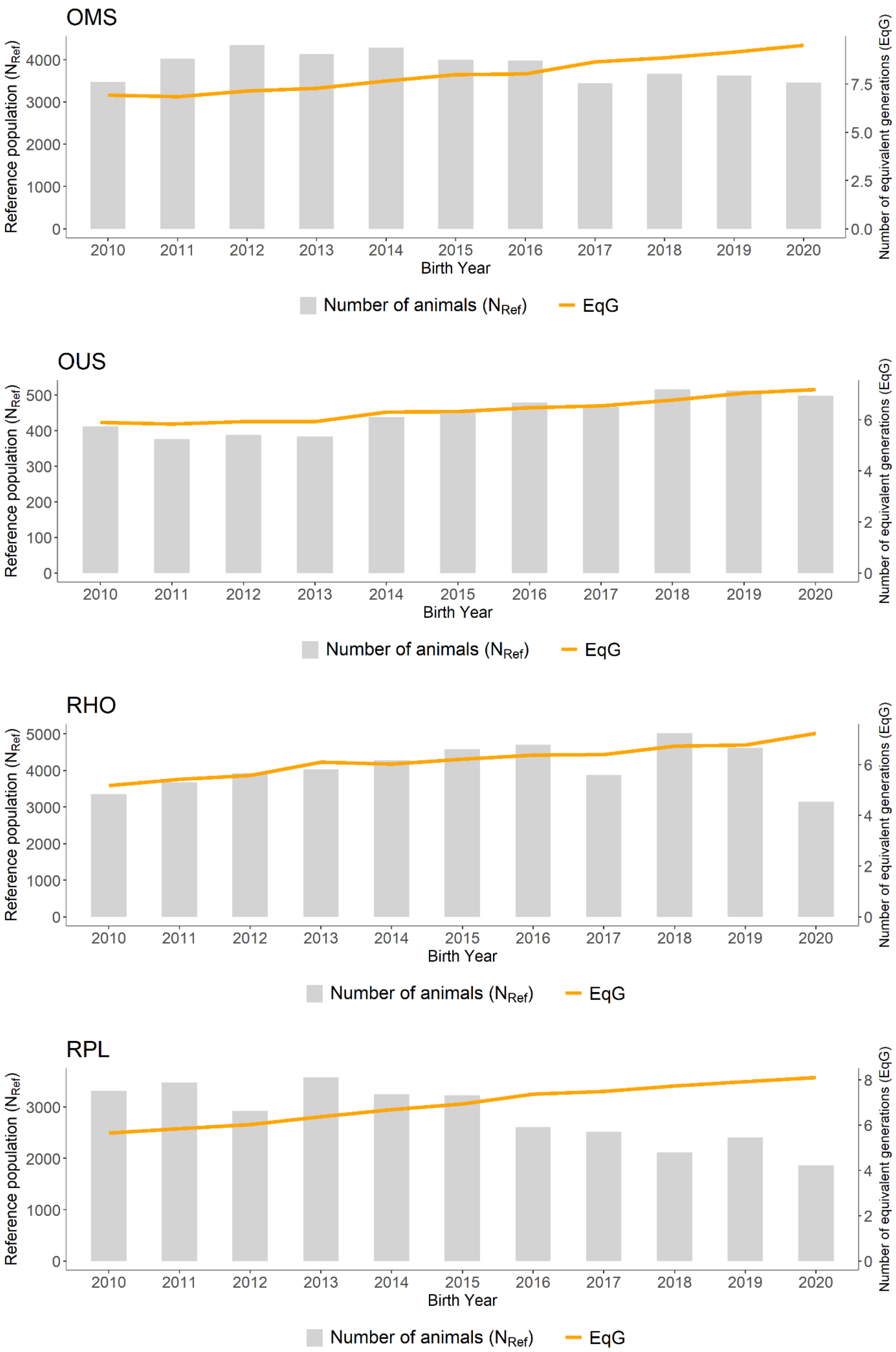
OMS
Reference population (N_{Ref})
0
1000
2000
3000
4000
Number of equivalent generations (EqG)
0.0
2.5
5.0
7.5
2010
2011
2012
2013
2014
2015
2016
2017
2018
2019
2020
Birth Year
Number of animals (N_{Ref})
EqG
OUS
Reference population (N_{Ref})
0
100
200
300
400
500
Number of equivalent generations (EqG)
0
2
4
6
2010
2011
2012
2013
2014
2015
2016
2017
2018
2019
2020
Birth Year
Number of animals (N_{Ref})
EqG
RHO
Reference population (N_{Ref})
0
1000
2000
3000
4000
5000
Number of equivalent generations (EqG)
0
2
4
6
2010
2011
2012
2013
2014
2015
2016
2017
2018
2019
2020
Birth Year
Number of animals (N_{Ref})
EqG
RPL
Reference population (N_{Ref})
0
1000
2000
3000
Number of equivalent generations (EqG)
0
2
4
6
8
2010
2011
2012
2013
2014
2015
2016
2017
2018
2019
2020
Birth Year
Number of animals (N_{Ref})
EqG

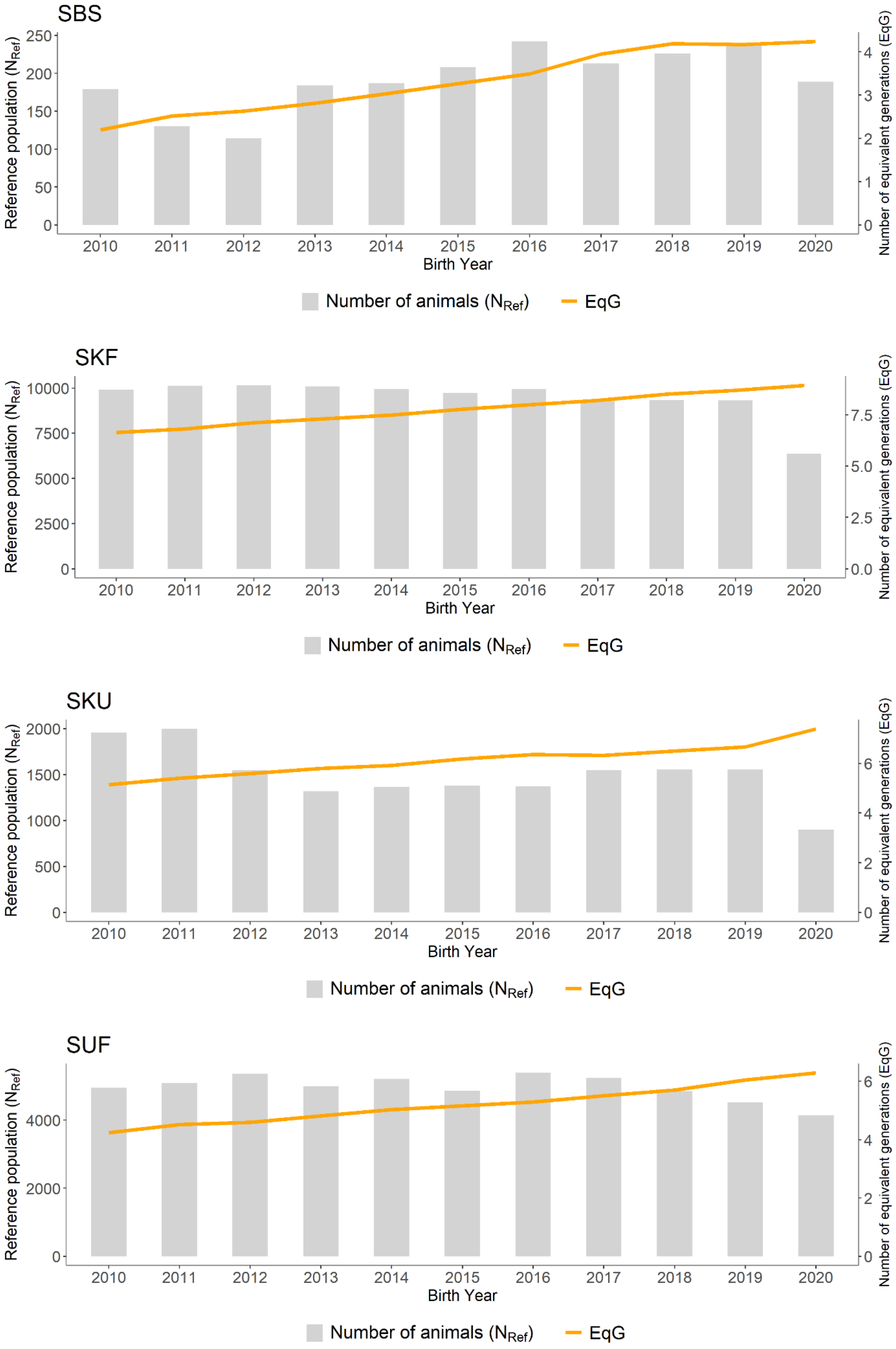
SBS
Reference population (N_{Ref})
Number of equivalent generations (EqG)
Birth Year
Number of animals (N_{Ref})
EqG
SKF
Reference population (N_{Ref})
Number of equivalent generations (EqG)
Birth Year
Number of animals (N_{Ref})
EqG
SKU
Reference population (N_{Ref})
Number of equivalent generations (EqG)
Birth Year
Number of animals (N_{Ref})
EqG
SUF
Reference population (N_{Ref})
Number of equivalent generations (EqG)
Birth Year
Number of animals (N_{Ref})
EqG

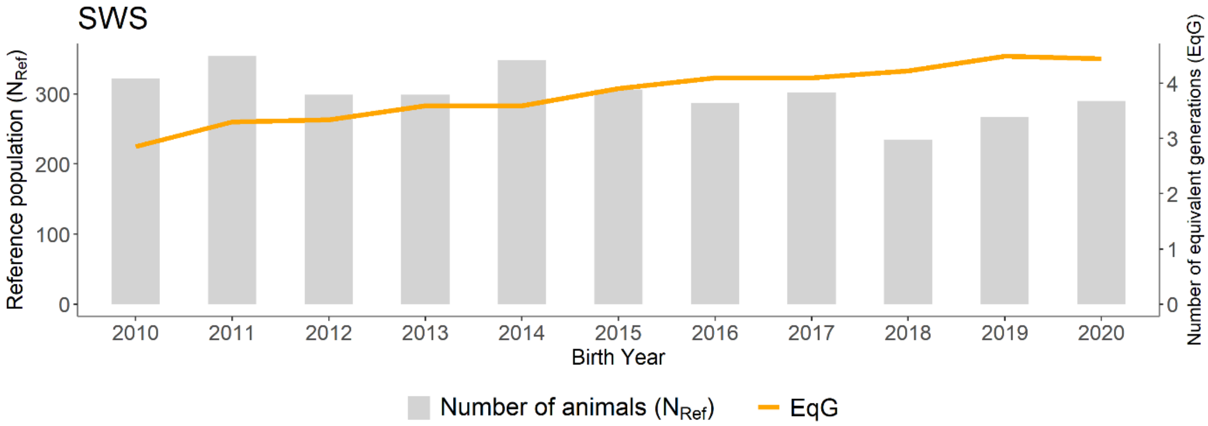
SWS
Reference population (N_Ref)
0
100
200
300
Number of equivalent generations (EqG)
0
1
2
3
4
2010
2011
2012
2013
2014
2015
2016
2017
2018
2019
2020
Birth Year
Number of animals (N_Ref)
EqG

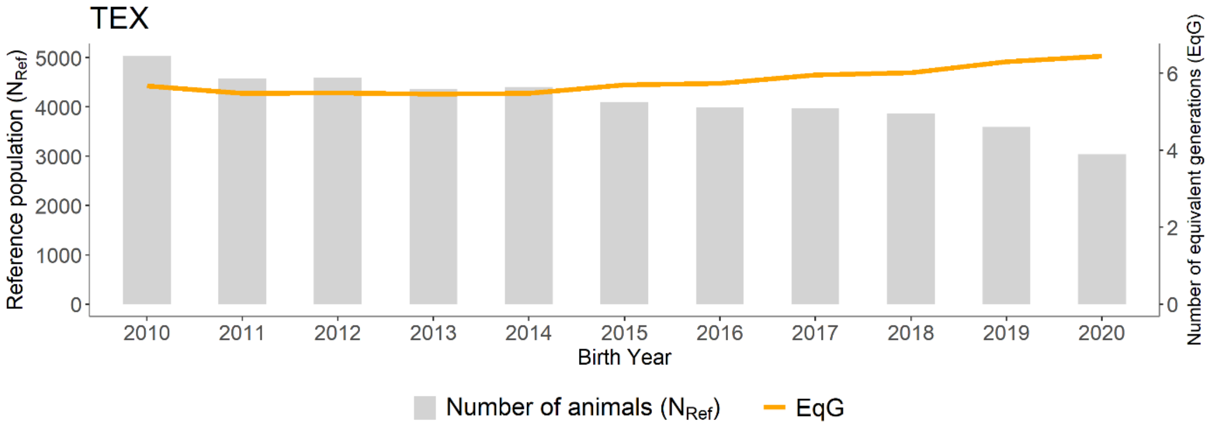
TEX
Reference population (N_Ref)
0
1000
2000
3000
4000
5000
Number of equivalent generations (EqG)
0
2
4
6
2010
2011
2012
2013
2014
2015
2016
2017
2018
2019
2020
Birth Year
Number of animals (N_Ref)
EqG

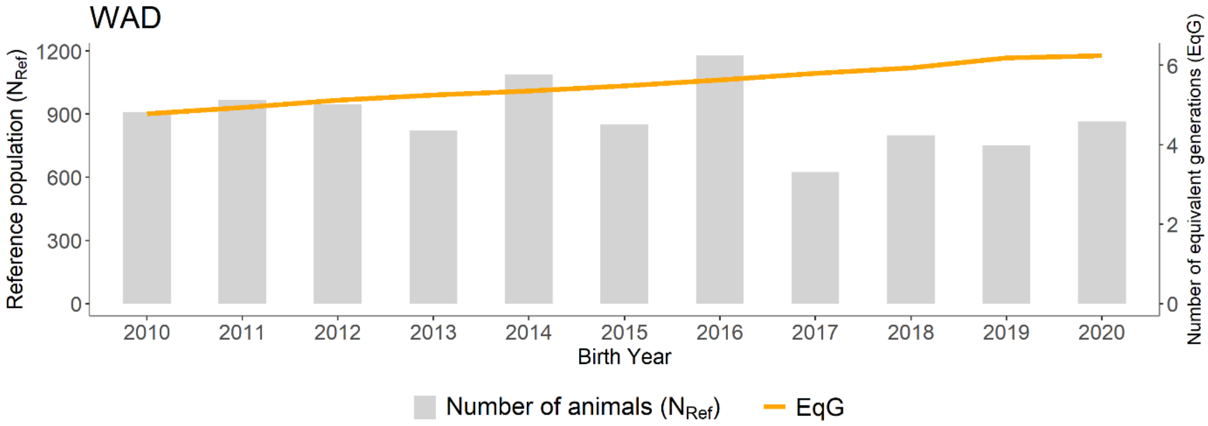
WAD
Reference population (N_Ref)
0
300
600
900
1200
Number of equivalent generations (EqG)
0
2
4
6
2010
2011
2012
2013
2014
2015
2016
2017
2018
2019
2020
Birth Year
Number of animals (N_Ref)
EqG

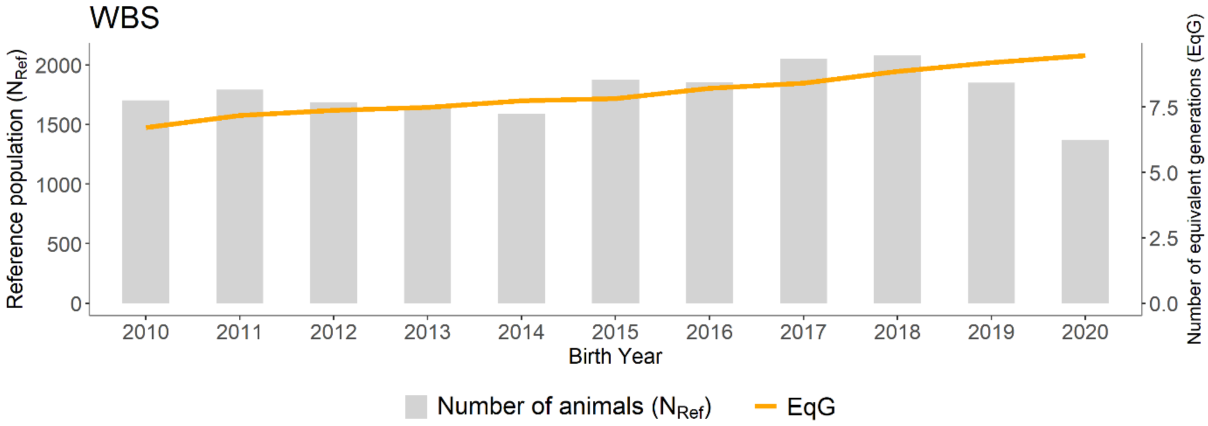
WBS
Reference population (N_Ref)
0
500
1000
1500
2000
Number of equivalent generations (EqG)
0.0
2.5
5.0
7.5
2010
2011
2012
2013
2014
2015
2016
2017
2018
2019
2020
Birth Year
Number of animals (N_Ref)
EqG

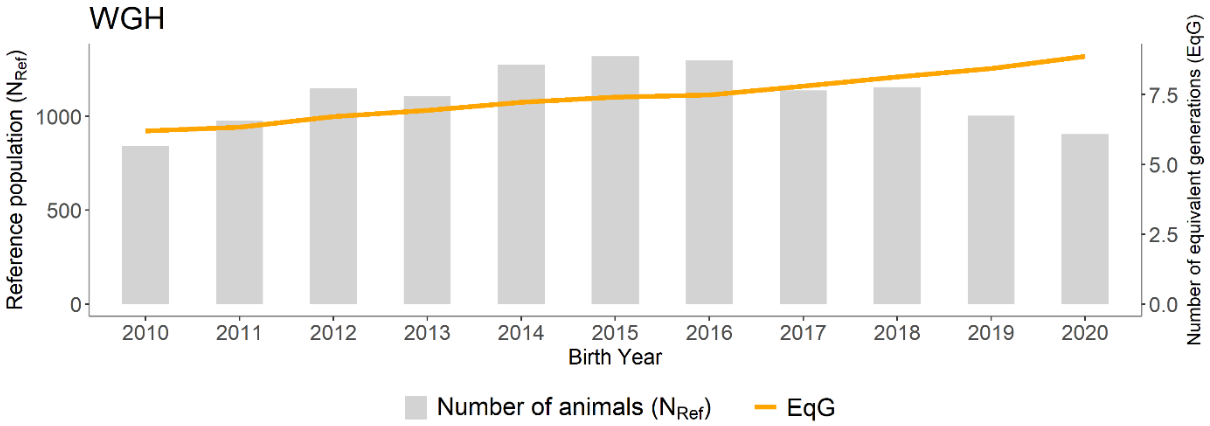
WGH
Reference population (N_{Ref})
0
500
1000
Number of equivalent generations (EqG)
0.0
2.5
5.0
7.5
2010 2011 2012 2013 2014 2015 2016 2017 2018 2019 2020
Birth Year
Number of animals (N_{Ref})
EqG

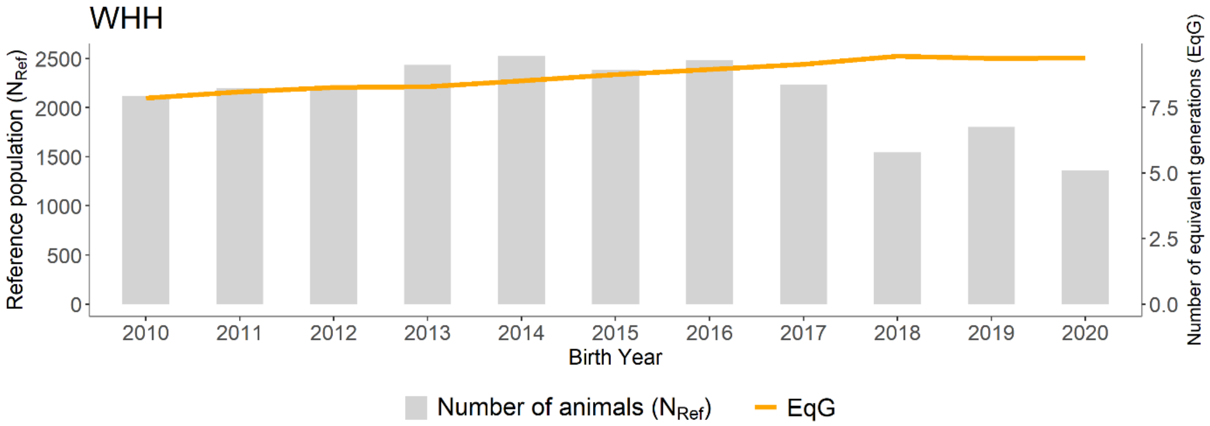
WHH
Reference population (N_{Ref})
0
500
1000
1500
2000
2500
Number of equivalent generations (EqG)
0.0
2.5
5.0
7.5
2010 2011 2012 2013 2014 2015 2016 2017 2018 2019 2020
Birth Year
Number of animals (N_{Ref})
EqG

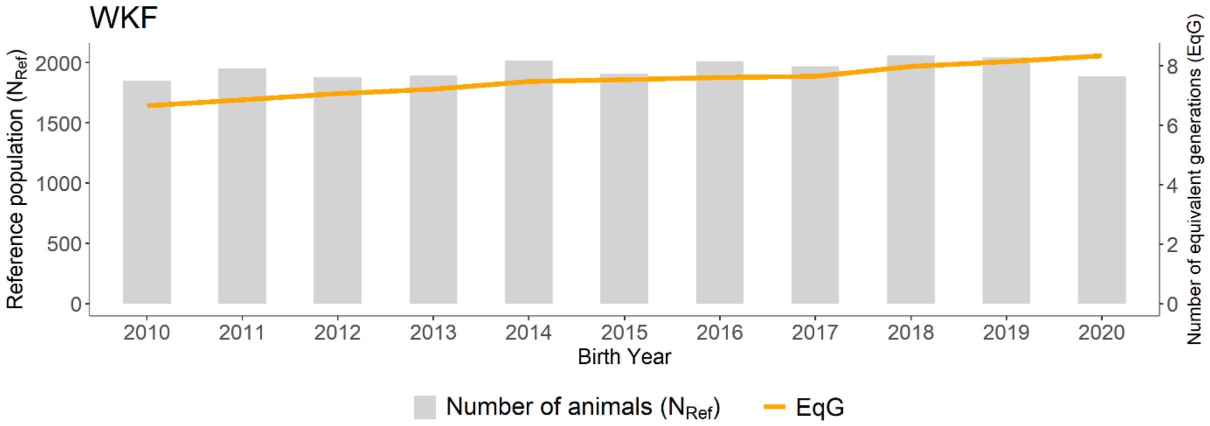
WKF
Reference population (N_{Ref})
0
500
1000
1500
2000
Number of equivalent generations (EqG)
0
2
4
6
8
2010 2011 2012 2013 2014 2015 2016 2017 2018 2019 2020
Birth Year
Number of animals (N_{Ref})
EqG

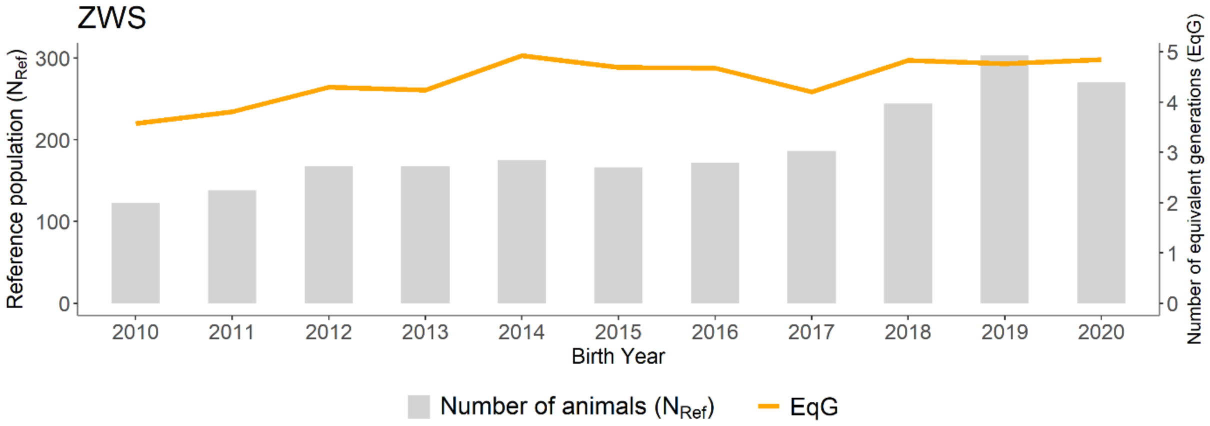
ZWS
Reference population (N_{Ref})
0
100
200
300
Number of equivalent generations (EqG)
0
1
2
3
4
5
2010 2011 2012 2013 2014 2015 2016 2017 2018 2019 2020
Birth Year
Number of animals (N_{Ref})
EqG
