# Supplementary material for: Genetic Diversity and Trends of Ancestral and New Inbreeding in German Sheep Breeds by Pedigree Data
Source: Animals (Basel). 2023 Feb 10;13(4):623. doi: 10.3390/ani13040623 (PMC9951766; doi:10.3390/ani13040623)
Supplement: Supplementary file 1 [file animals-13-00623-s001.zip › Figure S2-Coancestry-alpha.pdf]

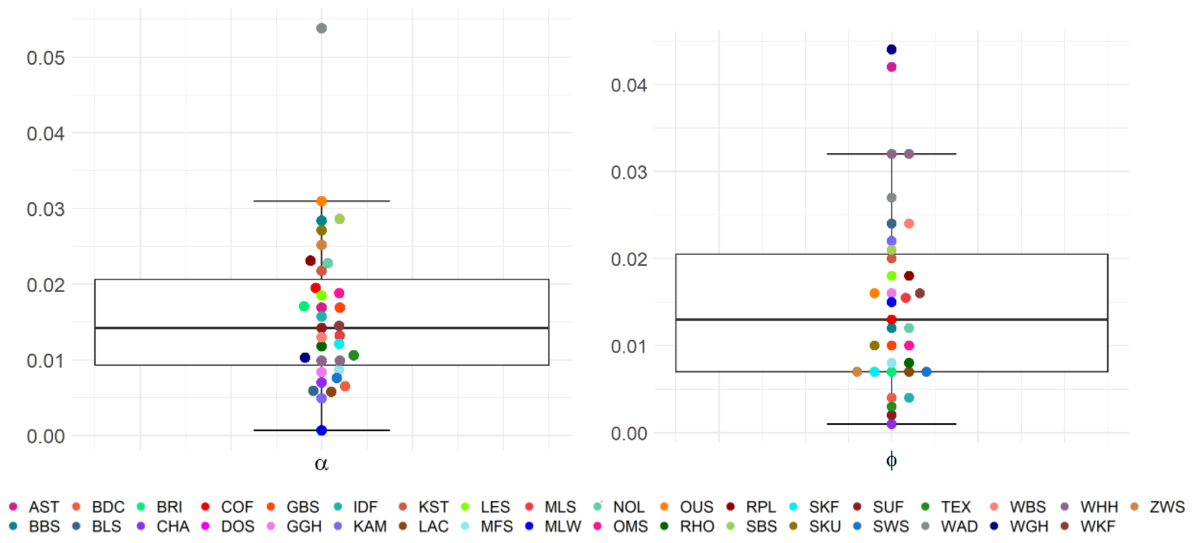

Figure S2. Degree of deviation of random mating from Hardy–Weinberg proportion (α) and average coancestry within the parental population (Φ) for the reference populations including birth years 2010–2020 in each of the 35 sheep breeds.
