# Supplementary material for: Genetic Diversity and Trends of Ancestral and New Inbreeding in German Sheep Breeds by Pedigree Data
Source: Animals (Basel). 2023 Feb 10;13(4):623. doi: 10.3390/ani13040623 (PMC9951766; doi:10.3390/ani13040623)
Supplement: Supplementary file 1 [file animals-13-00623-s001.zip › Figure S3-Inbreeding coefficient.pdf]

Figure S3. Inbreeding coefficients for all (F) and only inbred animals (F_{inbred}) as well the proportion of inbred animals for the birth years 2010 to 2020 for each of the 35 sheep breeds in Germany.

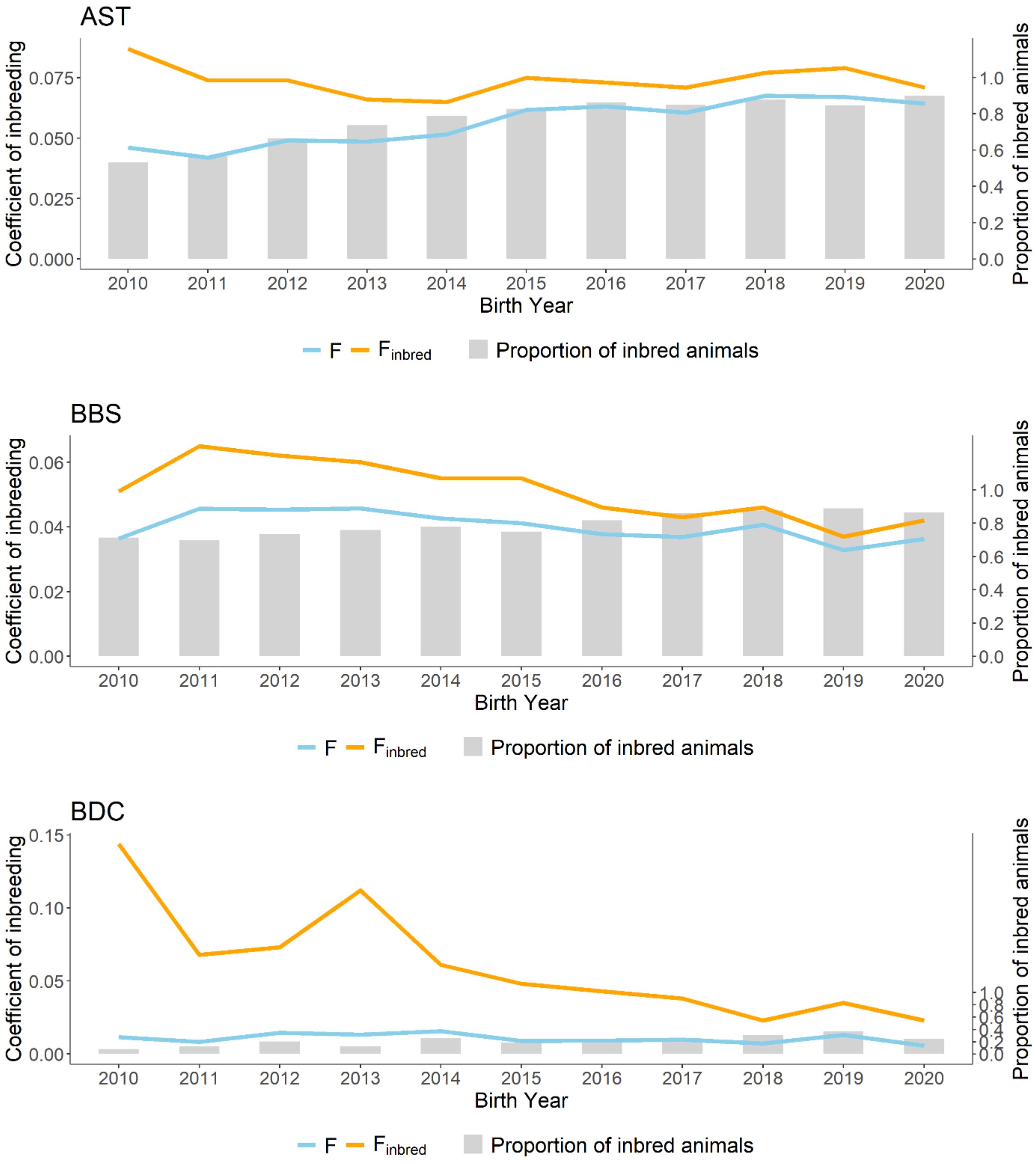

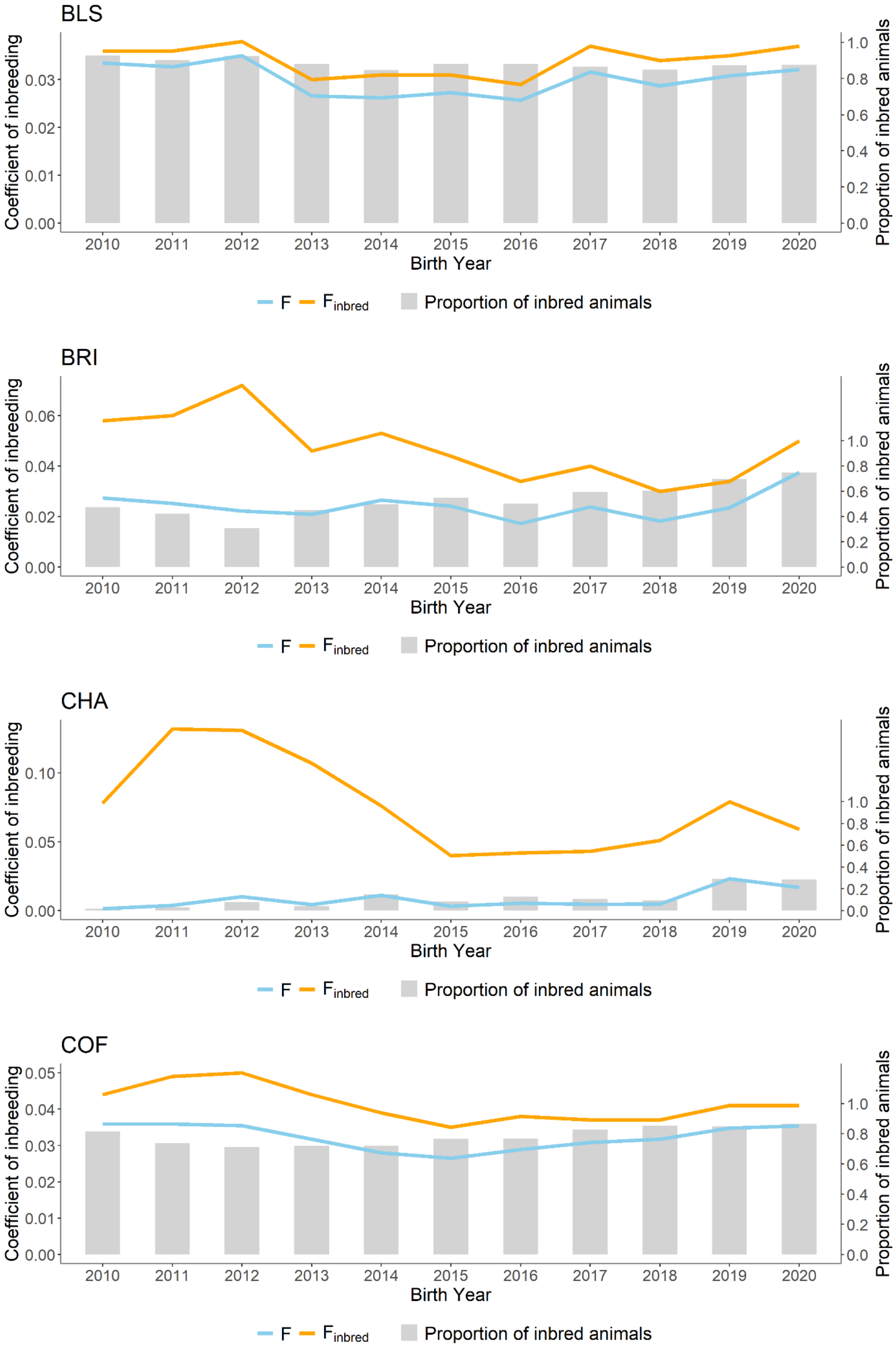
BLS
BRI
CHA
COF
Coefficient of inbreeding
Proportion of inbred animals
Birth Year
2010
2011
2012
2013
2014
2015
2016
2017
2018
2019
2020
F
F inbred
Proportion of inbred animals

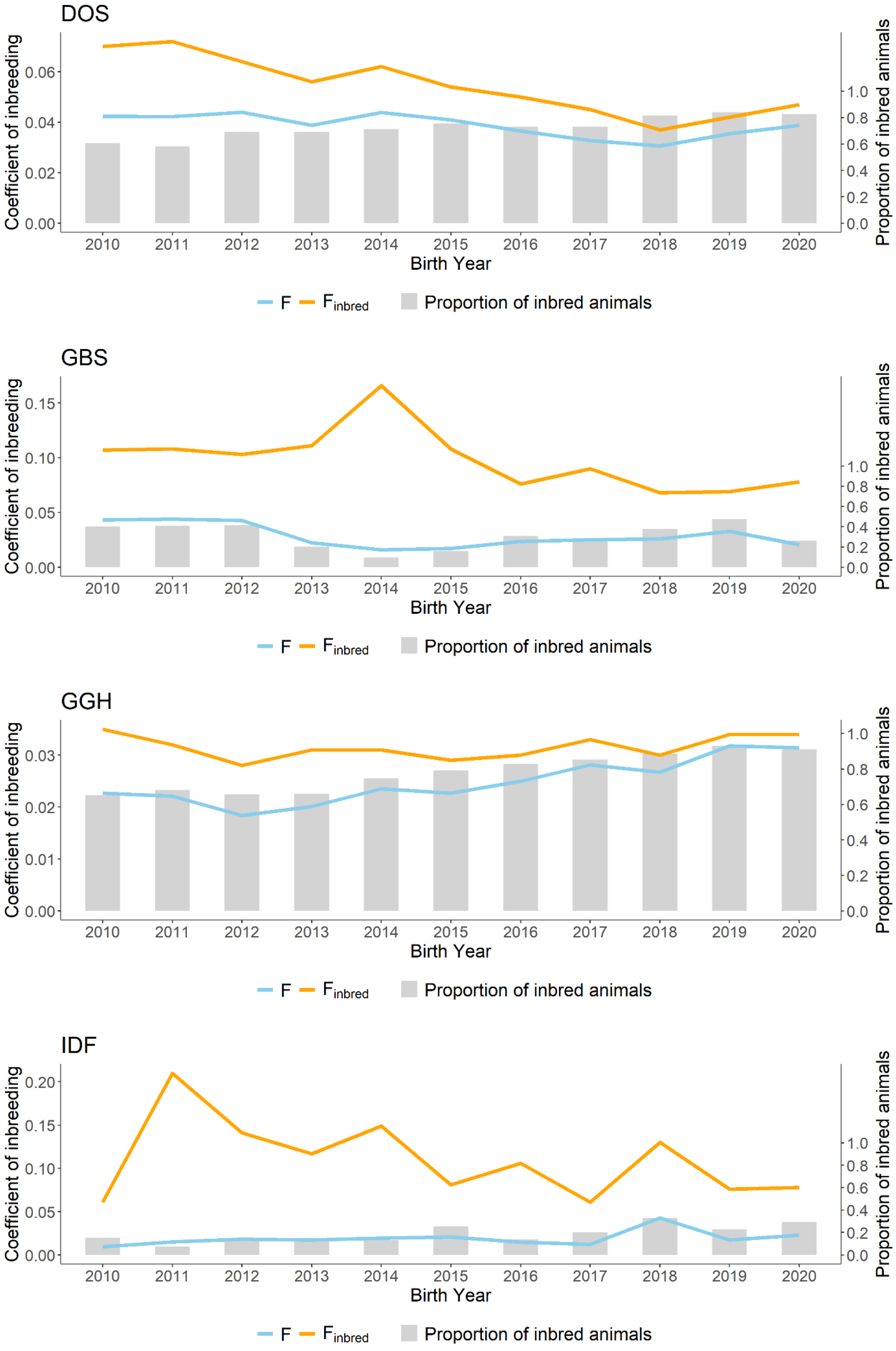
DOS
GBS
GGH
IDF
Coefficient of inbreeding
Proportion of inbred animals
Birth Year
2010
2011
2012
2013
2014
2015
2016
2017
2018
2019
2020
F
F_{inbred}
Proportion of inbred animals

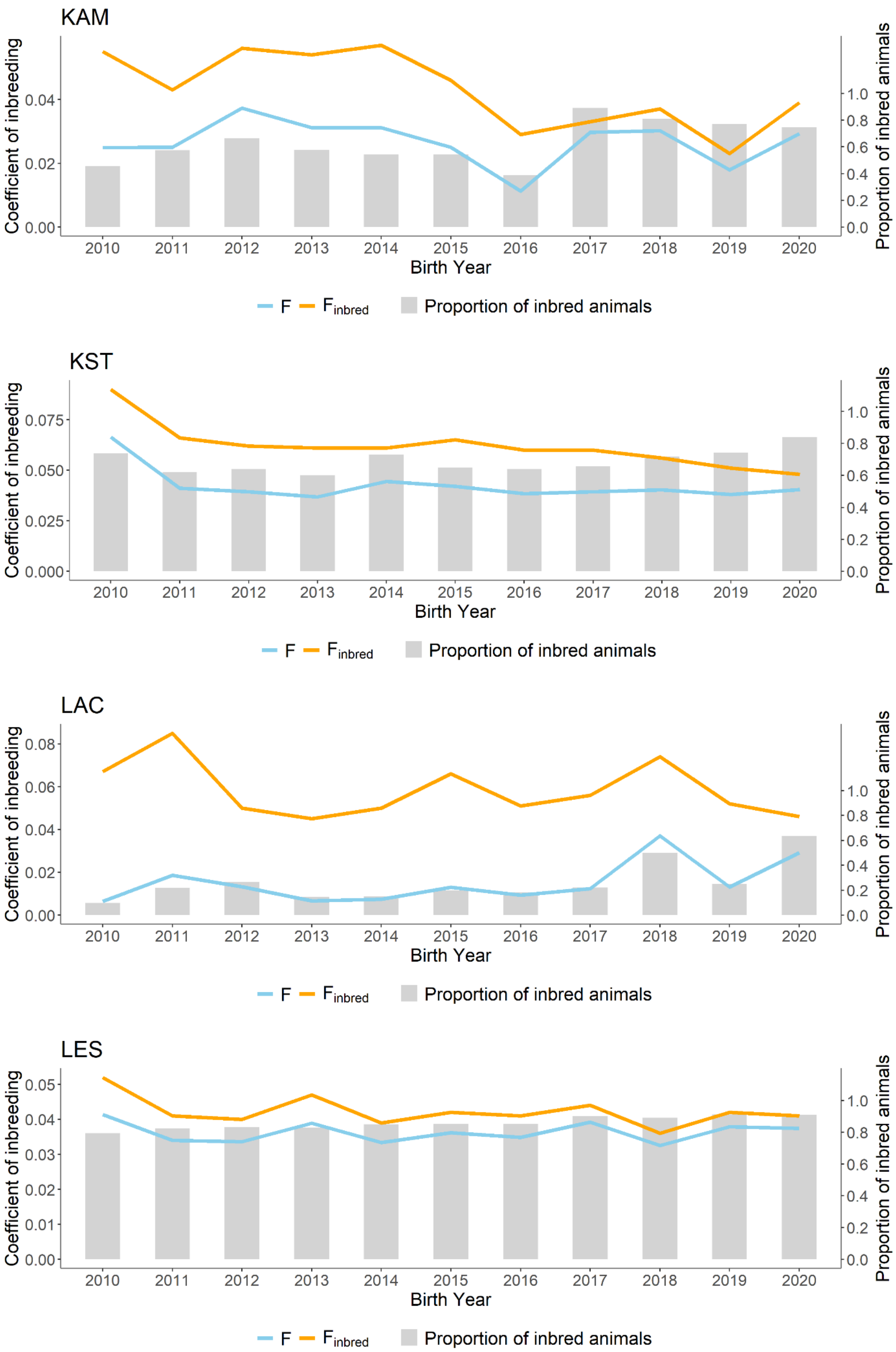

KAM
KST
LAC
LES
Coefficient of inbreeding
Proportion of inbred animals
Birth Year
2010
2011
2012
2013
2014
2015
2016
2017
2018
2019
2020
F
F inbred
Proportion of inbred animals

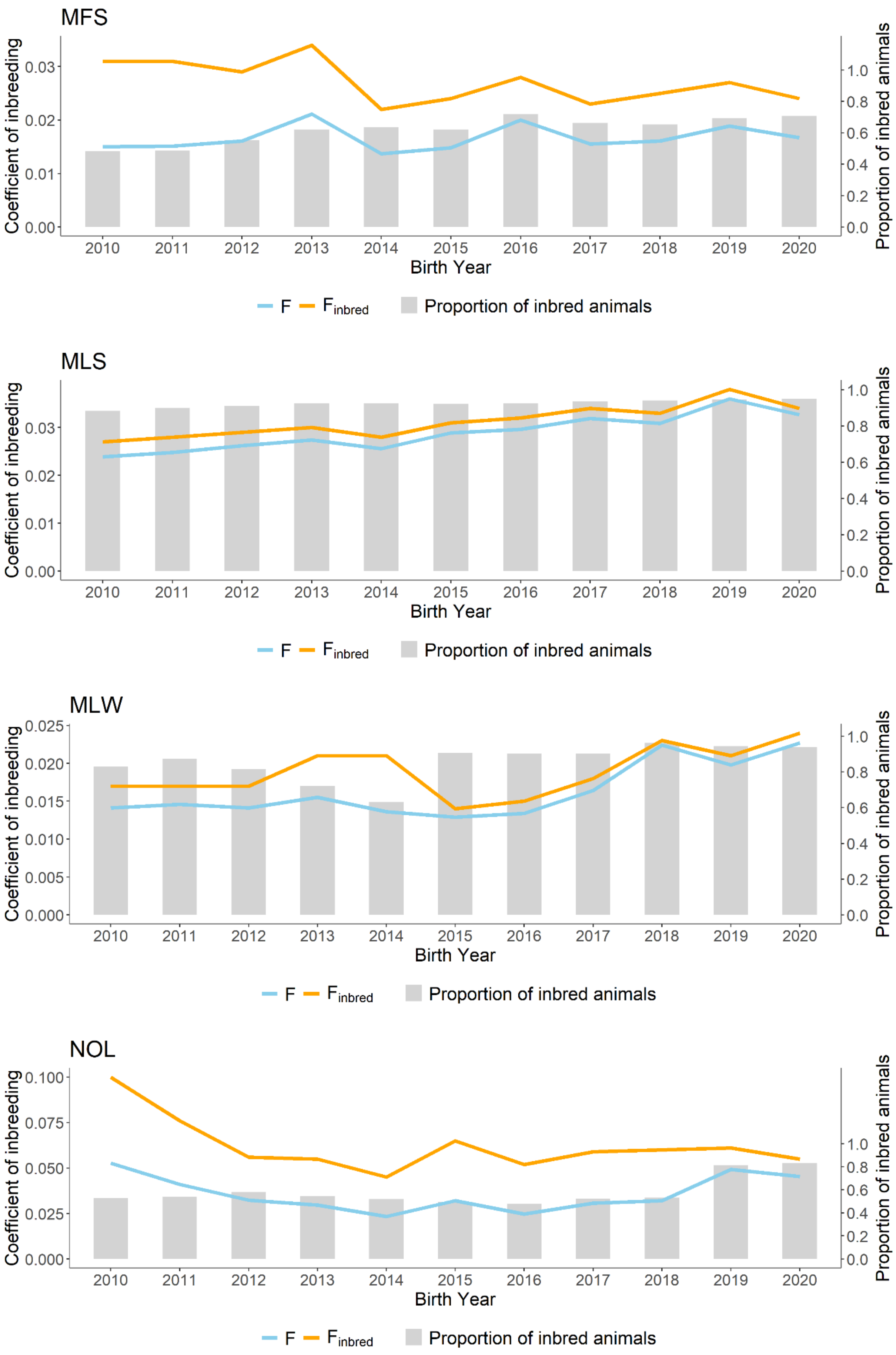
MFS
Coefficient of inbreeding
0.00
0.01
0.02
0.03
Proportion of inbred animals
0.0
0.2
0.4
0.6
0.8
1.0
2010
2011
2012
2013
2014
2015
2016
2017
2018
2019
2020
Birth Year
F
F_{inbred}
Proportion of inbred animals
MLS
Coefficient of inbreeding
0.00
0.01
0.02
0.03
Proportion of inbred animals
0.0
0.2
0.4
0.6
0.8
1.0
2010
2011
2012
2013
2014
2015
2016
2017
2018
2019
2020
Birth Year
F
F_{inbred}
Proportion of inbred animals
MLW
Coefficient of inbreeding
0.000
0.005
0.010
0.015
0.020
0.025
Proportion of inbred animals
0.0
0.2
0.4
0.6
0.8
1.0
2010
2011
2012
2013
2014
2015
2016
2017
2018
2019
2020
Birth Year
F
F_{inbred}
Proportion of inbred animals
NOL
Coefficient of inbreeding
0.000
0.025
0.050
0.075
0.100
Proportion of inbred animals
0.0
0.2
0.4
0.6
0.8
1.0
2010
2011
2012
2013
2014
2015
2016
2017
2018
2019
2020
Birth Year
F
F_{inbred}
Proportion of inbred animals

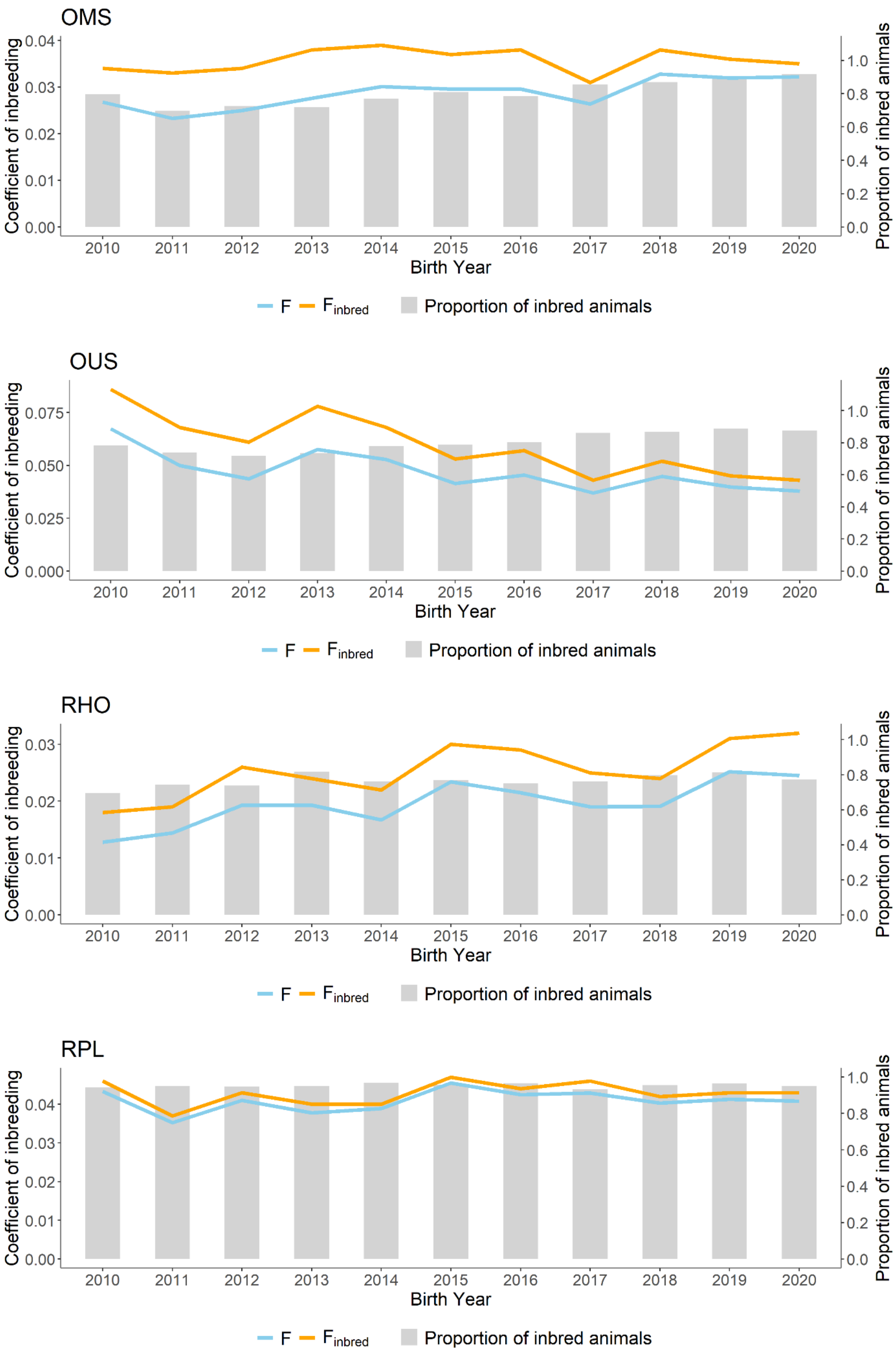
OMS
Coefficient of inbreeding
Proportion of inbred animals
0.00 0.01 0.02 0.03 0.04
0.0 0.2 0.4 0.6 0.8 1.0
2010 2011 2012 2013 2014 2015 2016 2017 2018 2019 2020
Birth Year
F F_{inbred} Proportion of inbred animals
OUS
Coefficient of inbreeding
Proportion of inbred animals
0.000 0.025 0.050 0.075
0.0 0.2 0.4 0.6 0.8 1.0
2010 2011 2012 2013 2014 2015 2016 2017 2018 2019 2020
Birth Year
F F_{inbred} Proportion of inbred animals
RHO
Coefficient of inbreeding
Proportion of inbred animals
0.00 0.01 0.02 0.03
0.0 0.2 0.4 0.6 0.8 1.0
2010 2011 2012 2013 2014 2015 2016 2017 2018 2019 2020
Birth Year
F F_{inbred} Proportion of inbred animals
RPL
Coefficient of inbreeding
Proportion of inbred animals
0.00 0.01 0.02 0.03 0.04
0.0 0.2 0.4 0.6 0.8 1.0
2010 2011 2012 2013 2014 2015 2016 2017 2018 2019 2020
Birth Year
F F_{inbred} Proportion of inbred animals

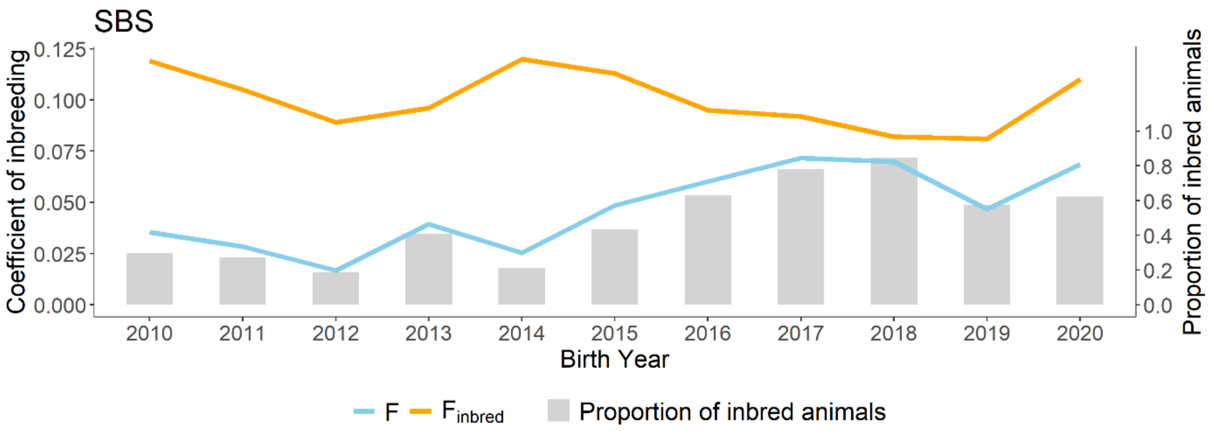
SBS
Coefficient of inbreeding
0.125
0.100
0.075
0.050
0.025
0.000
Proportion of inbred animals
1.0
0.8
0.6
0.4
0.2
0.0
2010 2011 2012 2013 2014 2015 2016 2017 2018 2019 2020
Birth Year
F
F inbred
Proportion of inbred animals

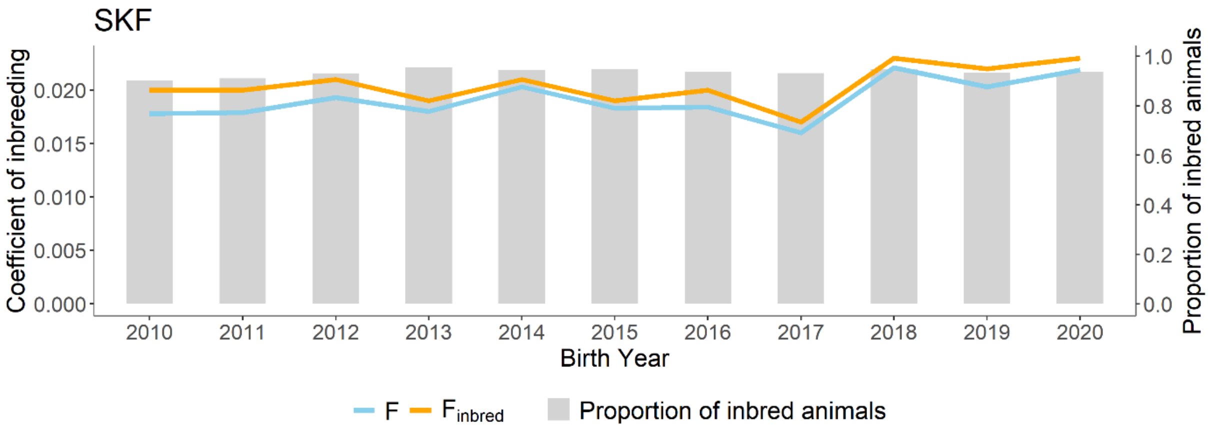
SKF
Coefficient of inbreeding
0.020
0.015
0.010
0.005
0.000
Proportion of inbred animals
1.0
0.8
0.6
0.4
0.2
0.0
2010 2011 2012 2013 2014 2015 2016 2017 2018 2019 2020
Birth Year
F
F inbred
Proportion of inbred animals

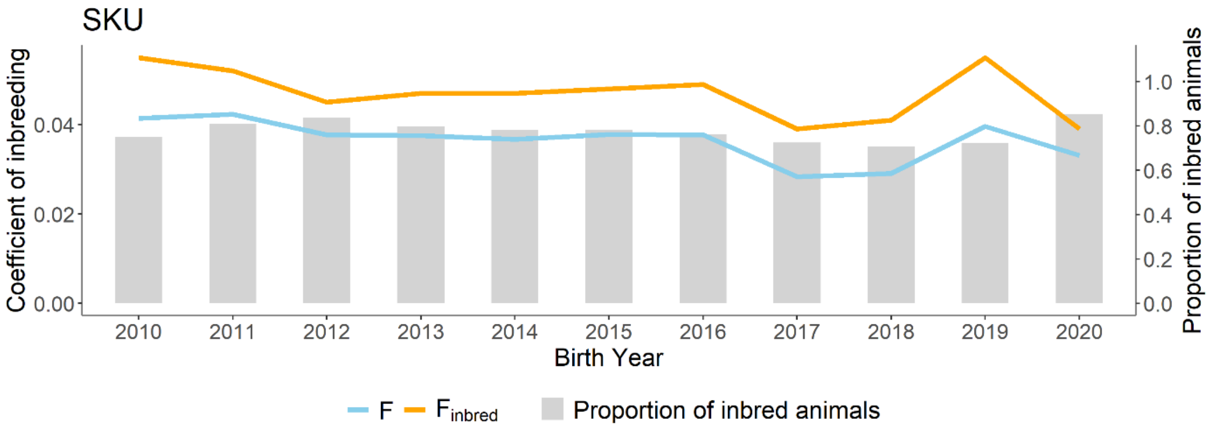
SKU
Coefficient of inbreeding
0.04
0.02
0.00
Proportion of inbred animals
1.0
0.8
0.6
0.4
0.2
0.0
2010 2011 2012 2013 2014 2015 2016 2017 2018 2019 2020
Birth Year
F
F inbred
Proportion of inbred animals

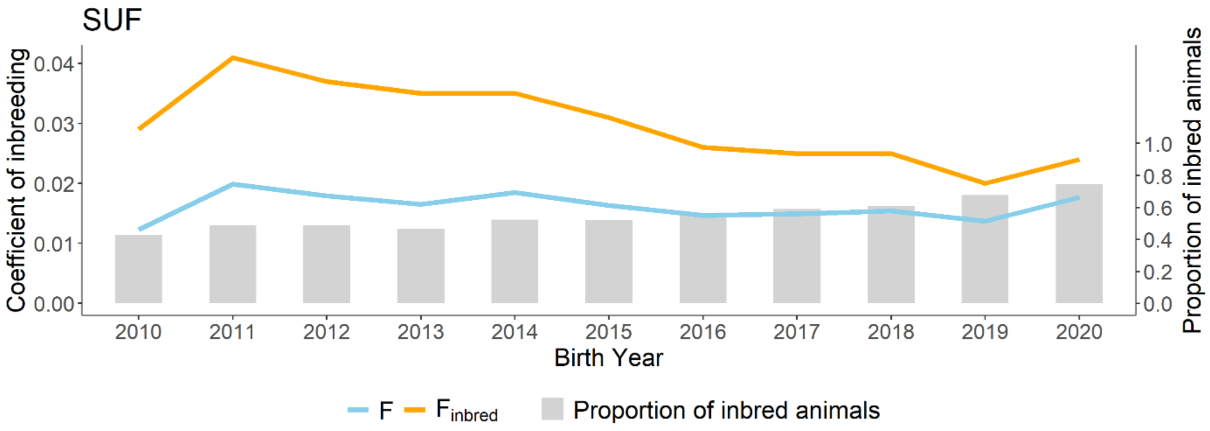
SUF
Coefficient of inbreeding
0.04
0.03
0.02
0.01
0.00
Proportion of inbred animals
1.0
0.8
0.6
0.4
0.2
0.0
2010 2011 2012 2013 2014 2015 2016 2017 2018 2019 2020
Birth Year
F
F inbred
Proportion of inbred animals

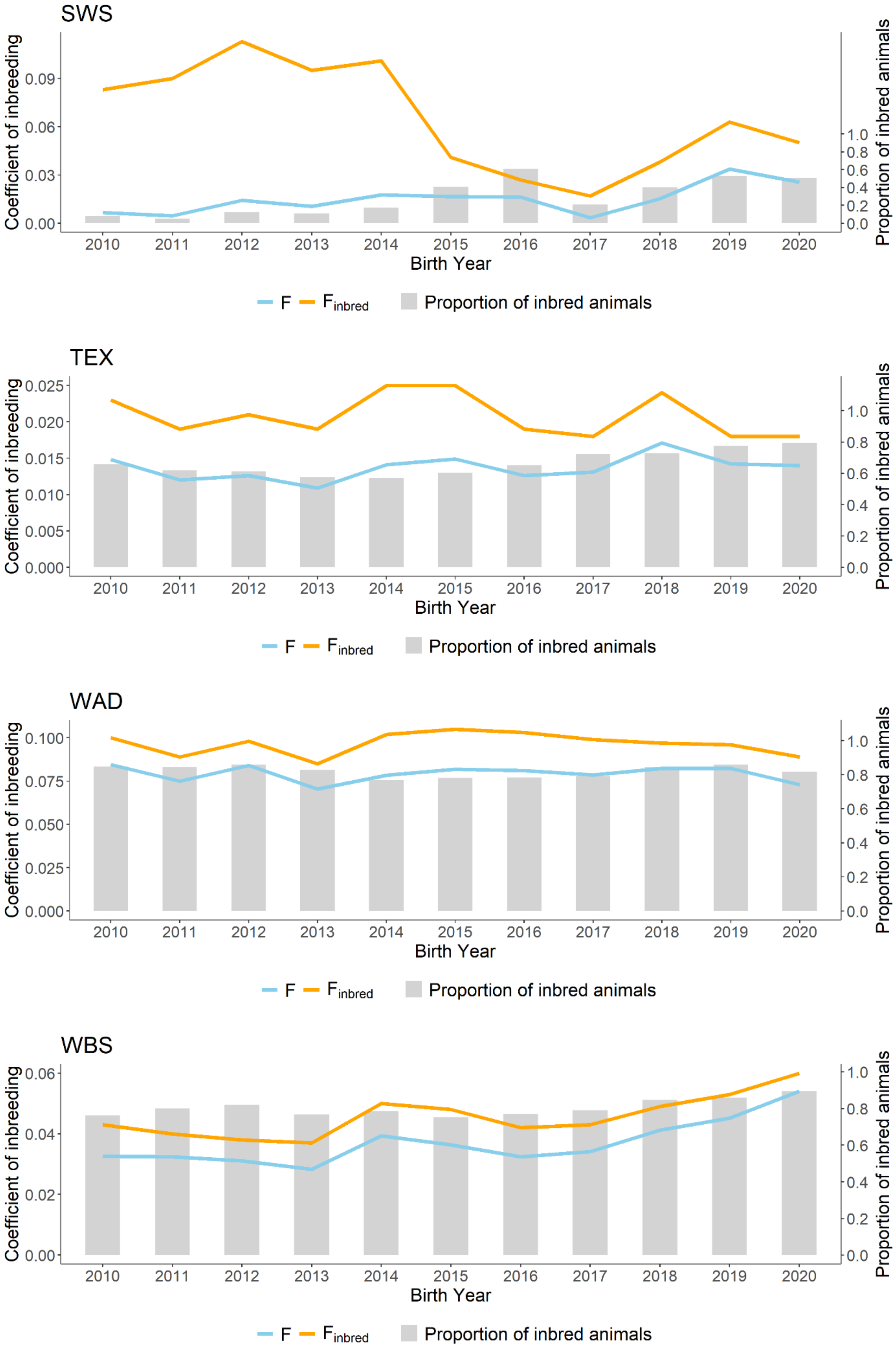
SWS
Coefficient of inbreeding
0.00
0.03
0.06
0.09
Proportion of inbred animals
0.0
0.2
0.4
0.6
0.8
1.0
2010
2011
2012
2013
2014
2015
2016
2017
2018
2019
2020
Birth Year
F
F_{inbred}
Proportion of inbred animals
TEX
Coefficient of inbreeding
0.000
0.005
0.010
0.015
0.020
0.025
Proportion of inbred animals
0.0
0.2
0.4
0.6
0.8
1.0
2010
2011
2012
2013
2014
2015
2016
2017
2018
2019
2020
Birth Year
F
F_{inbred}
Proportion of inbred animals
WAD
Coefficient of inbreeding
0.000
0.025
0.050
0.075
0.100
Proportion of inbred animals
0.0
0.2
0.4
0.6
0.8
1.0
2010
2011
2012
2013
2014
2015
2016
2017
2018
2019
2020
Birth Year
F
F_{inbred}
Proportion of inbred animals
WBS
Coefficient of inbreeding
0.00
0.02
0.04
0.06
Proportion of inbred animals
0.0
0.2
0.4
0.6
0.8
1.0
2010
2011
2012
2013
2014
2015
2016
2017
2018
2019
2020
Birth Year
F
F_{inbred}
Proportion of inbred animals

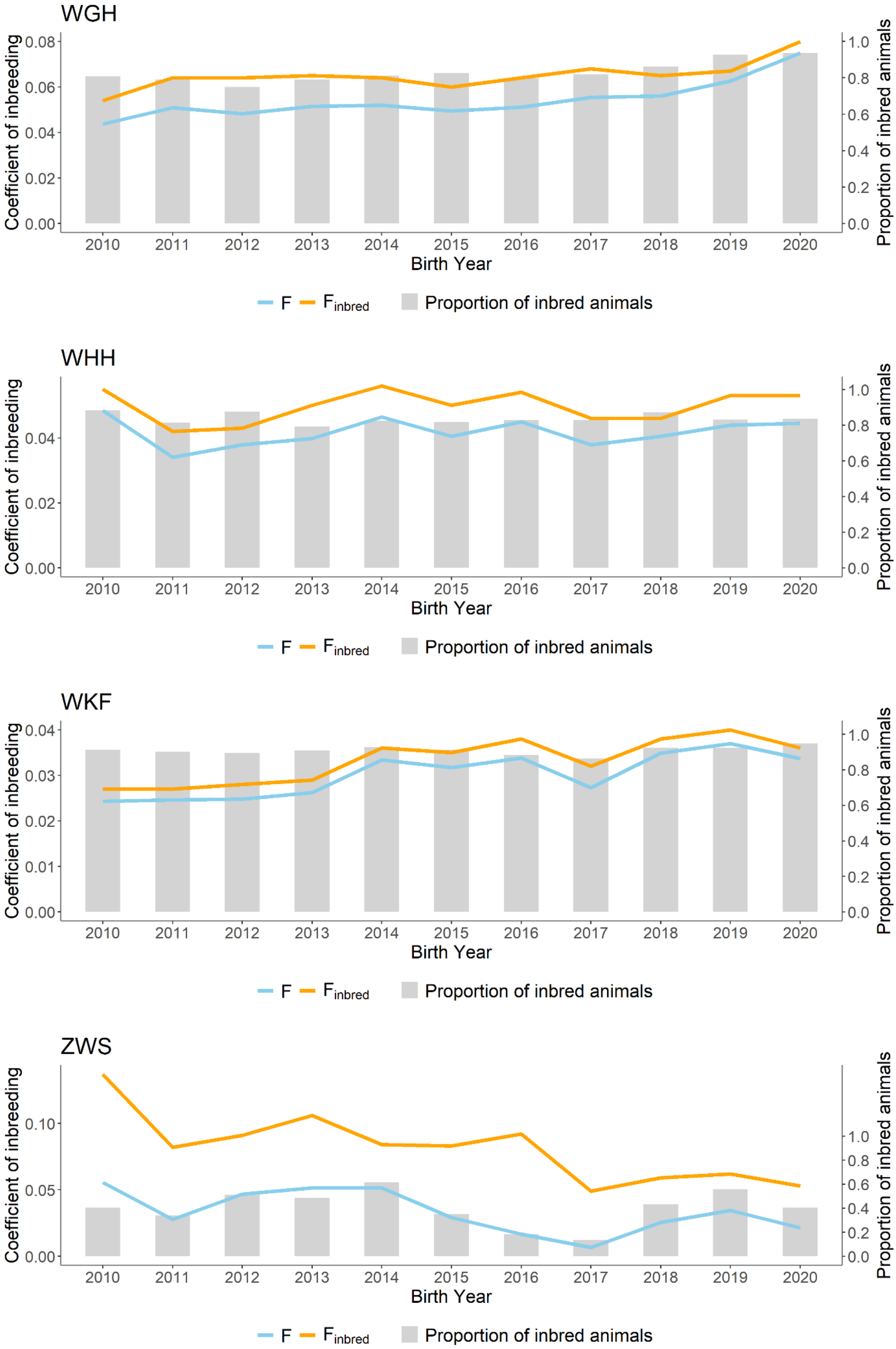
WGH
Coefficient of inbreeding
0.08
0.06
0.04
0.02
0.00
Proportion of inbred animals
1.0
0.8
0.6
0.4
0.2
0.0
2010 2011 2012 2013 2014 2015 2016 2017 2018 2019 2020
Birth Year
F
F inbred
Proportion of inbred animals
WHH
Coefficient of inbreeding
0.04
0.02
0.00
Proportion of inbred animals
1.0
0.8
0.6
0.4
0.2
0.0
2010 2011 2012 2013 2014 2015 2016 2017 2018 2019 2020
Birth Year
F
F inbred
Proportion of inbred animals
WKF
Coefficient of inbreeding
0.04
0.03
0.02
0.01
0.00
Proportion of inbred animals
1.0
0.8
0.6
0.4
0.2
0.0
2010 2011 2012 2013 2014 2015 2016 2017 2018 2019 2020
Birth Year
F
F inbred
Proportion of inbred animals
ZWS
Coefficient of inbreeding
0.10
0.05
0.00
Proportion of inbred animals
1.0
0.8
0.6
0.4
0.2
0.0
2010 2011 2012 2013 2014 2015 2016 2017 2018 2019 2020
Birth Year
F
F inbred
Proportion of inbred animals
