# Supplementary material for: Genetic Diversity and Trends of Ancestral and New Inbreeding in German Sheep Breeds by Pedigree Data
Source: Animals (Basel). 2023 Feb 10;13(4):623. doi: 10.3390/ani13040623 (PMC9951766; doi:10.3390/ani13040623)
Supplement: Supplementary file 1 [file animals-13-00623-s001.zip › Figure S4-rate of inbreeding.pdf]

Figure S4. Individual rate of inbreeding (ΔF_i) and realized effective population size (N_e) for the birth years 2010 to 2020 for each of the 35 sheep breeds in Germany.

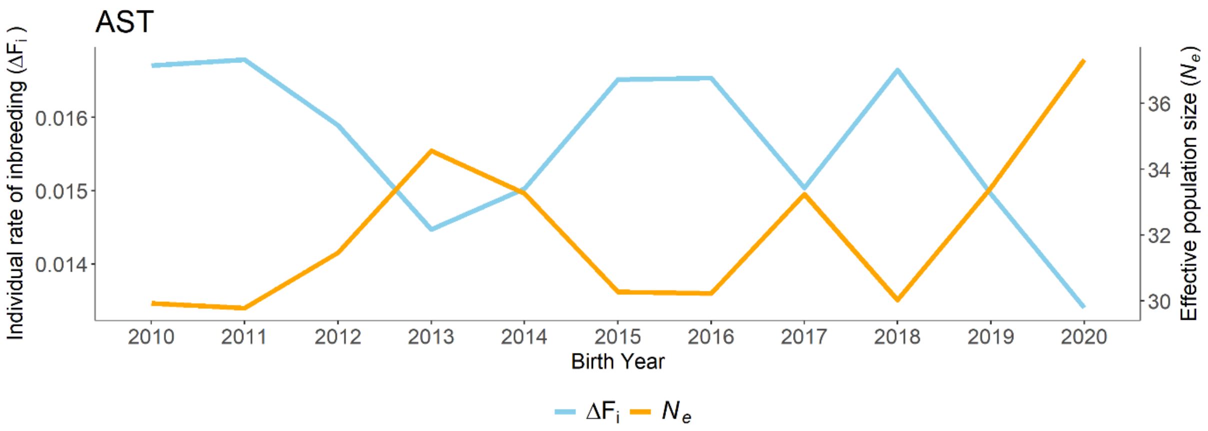

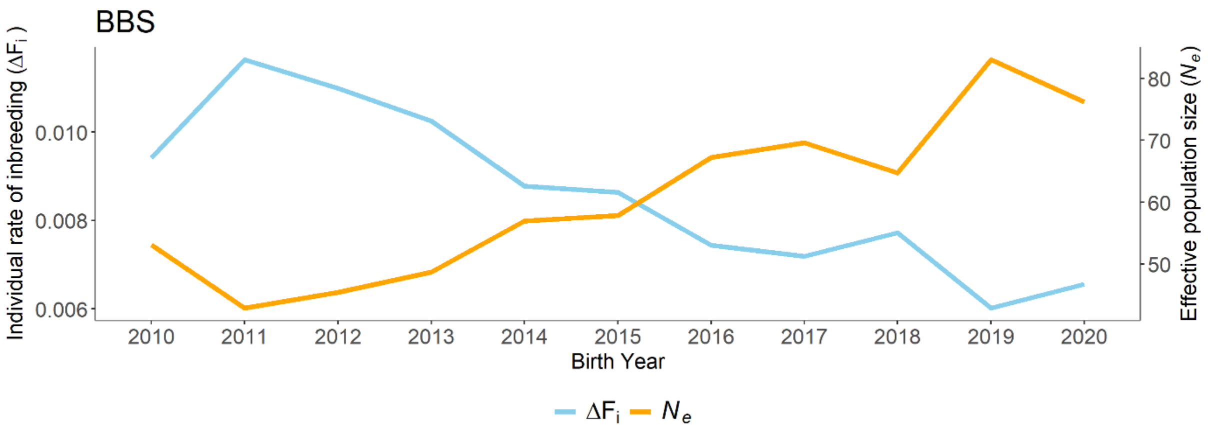

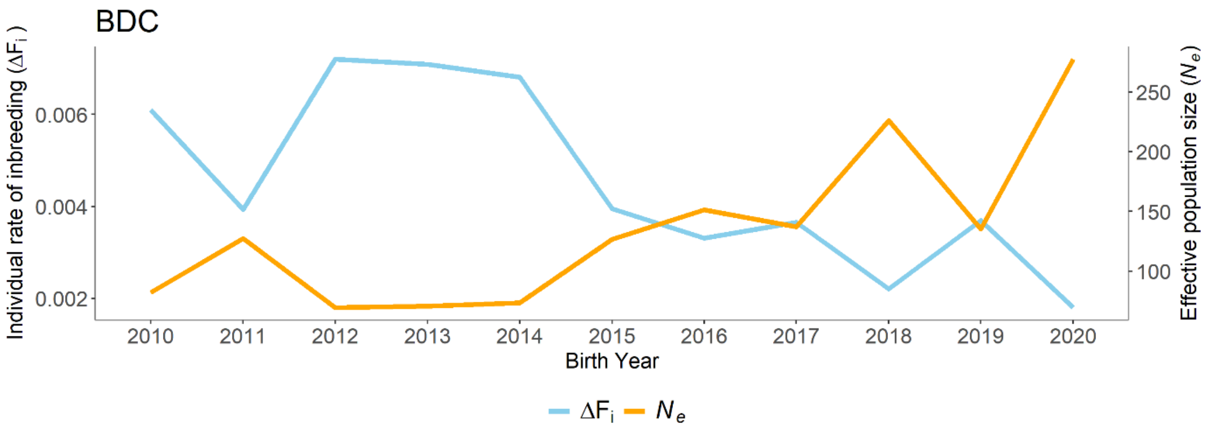

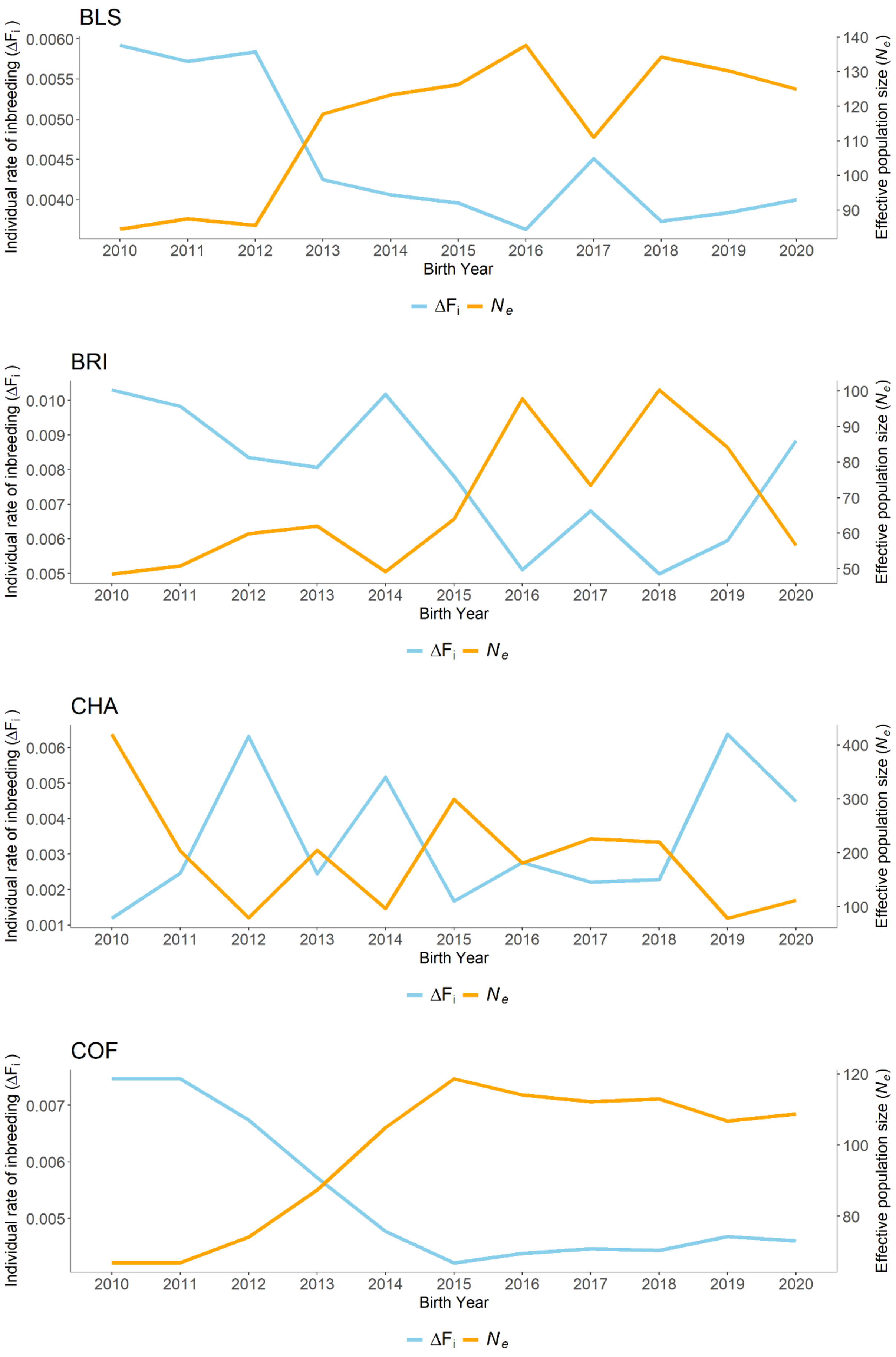
BLS
BRI
CHA
COF
Individual rate of inbreeding (ΔFi)
Effective population size (N_e)
Birth Year
2010
2011
2012
2013
2014
2015
2016
2017
2018
2019
2020
ΔFi
N_e
0.0060
0.0055
0.0050
0.0045
0.0040
140
130
120
110
100
90
0.010
0.009
0.008
0.007
0.006
0.005
100
90
80
70
60
50
0.006
0.005
0.004
0.003
0.002
0.001
400
300
200
100
0.007
0.006
0.005
120
100
80

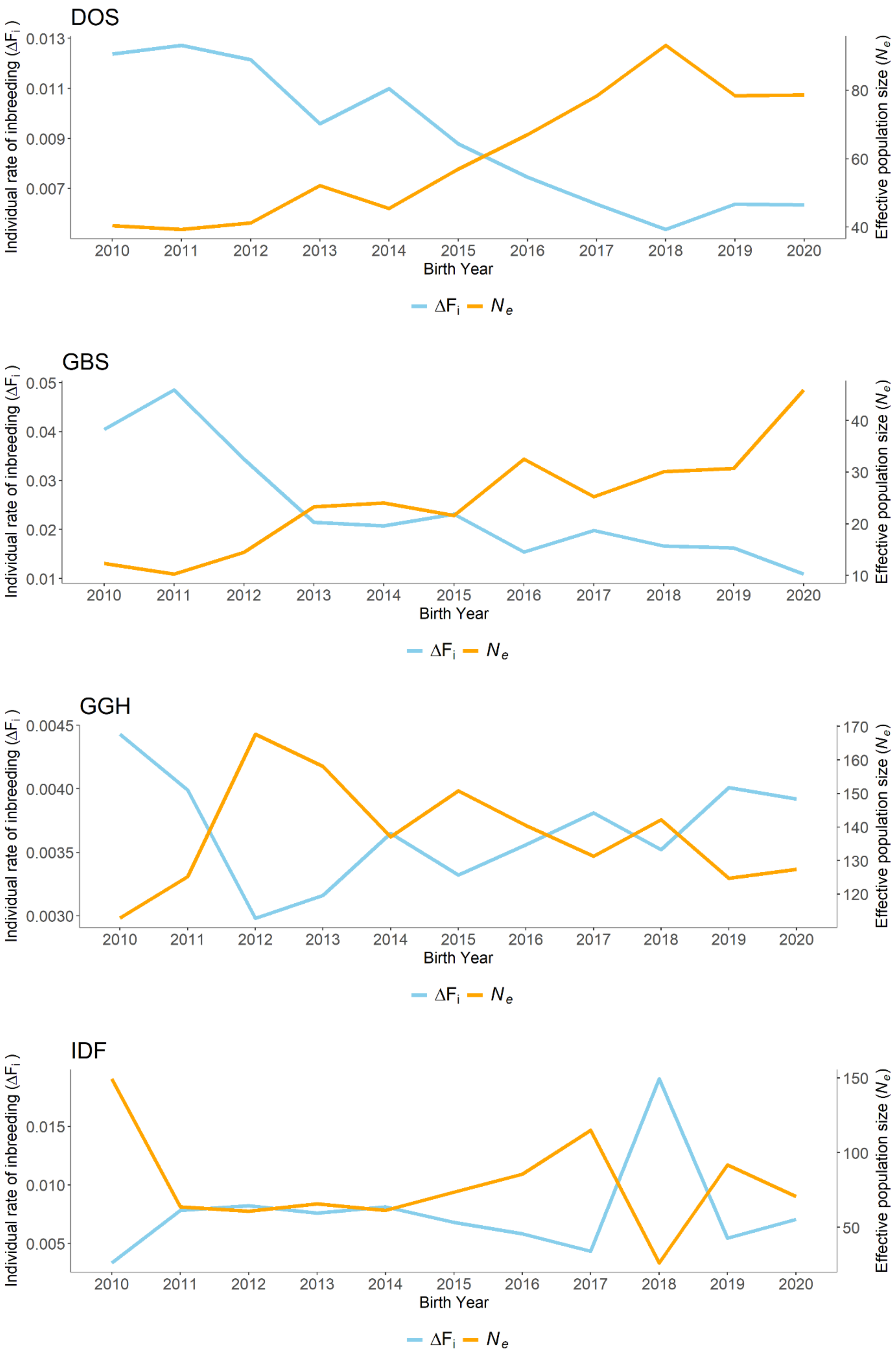
DOS
Individual rate of inbreeding (ΔFi)
0.013
0.011
0.009
0.007
Effective population size (Ne)
80
60
40
2010 2011 2012 2013 2014 2015 2016 2017 2018 2019 2020
Birth Year
ΔFi Ne
GBS
Individual rate of inbreeding (ΔFi)
0.05
0.04
0.03
0.02
0.01
Effective population size (Ne)
40
30
20
10
2010 2011 2012 2013 2014 2015 2016 2017 2018 2019 2020
Birth Year
ΔFi Ne
GGH
Individual rate of inbreeding (ΔFi)
0.0045
0.0040
0.0035
0.0030
Effective population size (Ne)
170
160
150
140
130
120
2010 2011 2012 2013 2014 2015 2016 2017 2018 2019 2020
Birth Year
ΔFi Ne
IDF
Individual rate of inbreeding (ΔFi)
0.015
0.010
0.005
Effective population size (Ne)
150
100
50
2010 2011 2012 2013 2014 2015 2016 2017 2018 2019 2020
Birth Year
ΔFi Ne

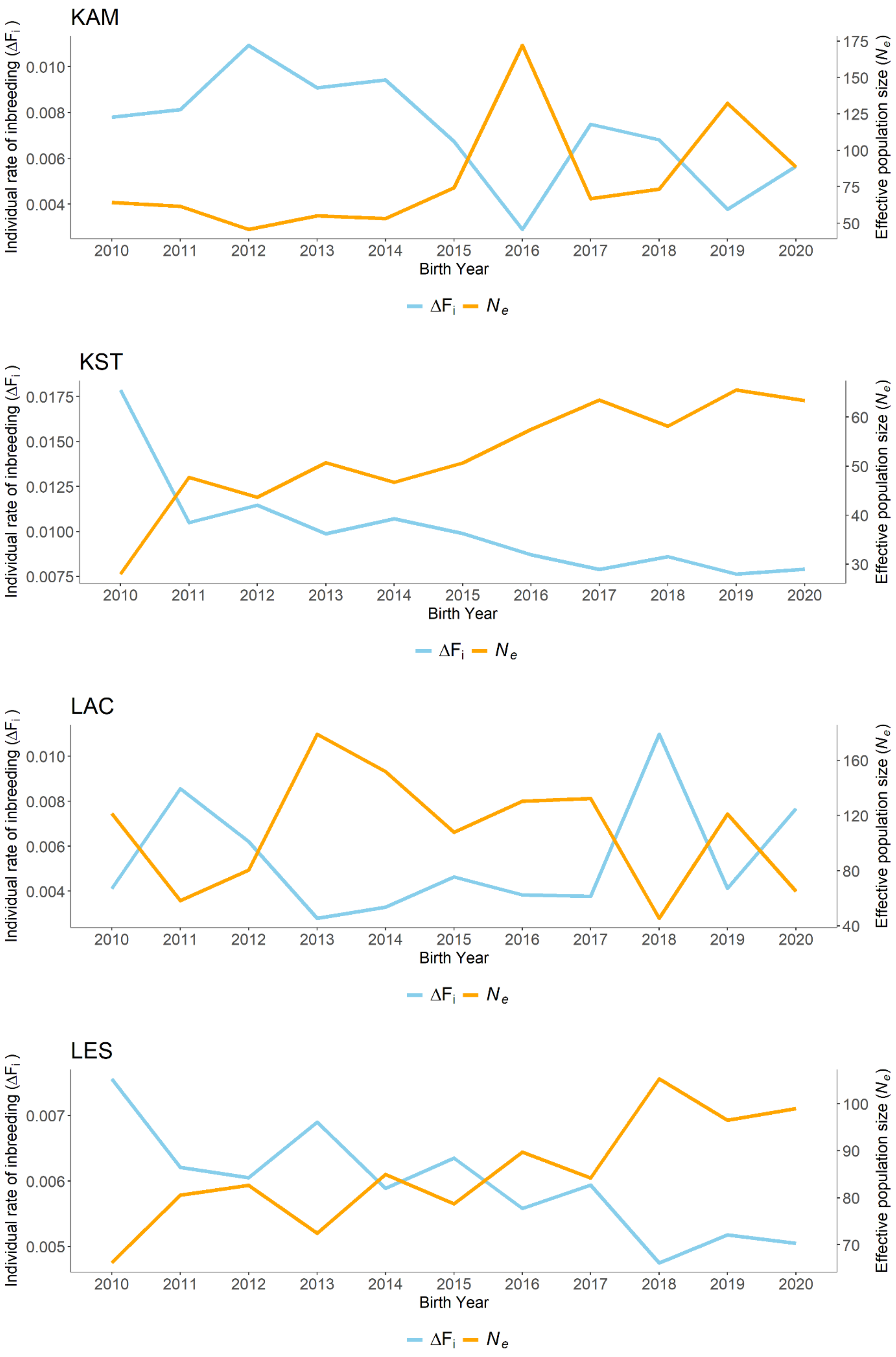

KAM
Individual rate of inbreeding (ΔFi)
0.010
0.008
0.006
0.004
175
150
125
100
75
50
Effective population size (Ne)
2010 2011 2012 2013 2014 2015 2016 2017 2018 2019 2020
Birth Year
ΔFi Ne
KST
Individual rate of inbreeding (ΔFi)
0.0175
0.0150
0.0125
0.0100
0.0075
60
50
40
30
Effective population size (Ne)
2010 2011 2012 2013 2014 2015 2016 2017 2018 2019 2020
Birth Year
ΔFi Ne
LAC
Individual rate of inbreeding (ΔFi)
0.010
0.008
0.006
0.004
160
120
80
40
Effective population size (Ne)
2010 2011 2012 2013 2014 2015 2016 2017 2018 2019 2020
Birth Year
ΔFi Ne
LES
Individual rate of inbreeding (ΔFi)
0.007
0.006
0.005
100
90
80
70
Effective population size (Ne)
2010 2011 2012 2013 2014 2015 2016 2017 2018 2019 2020
Birth Year
ΔFi Ne

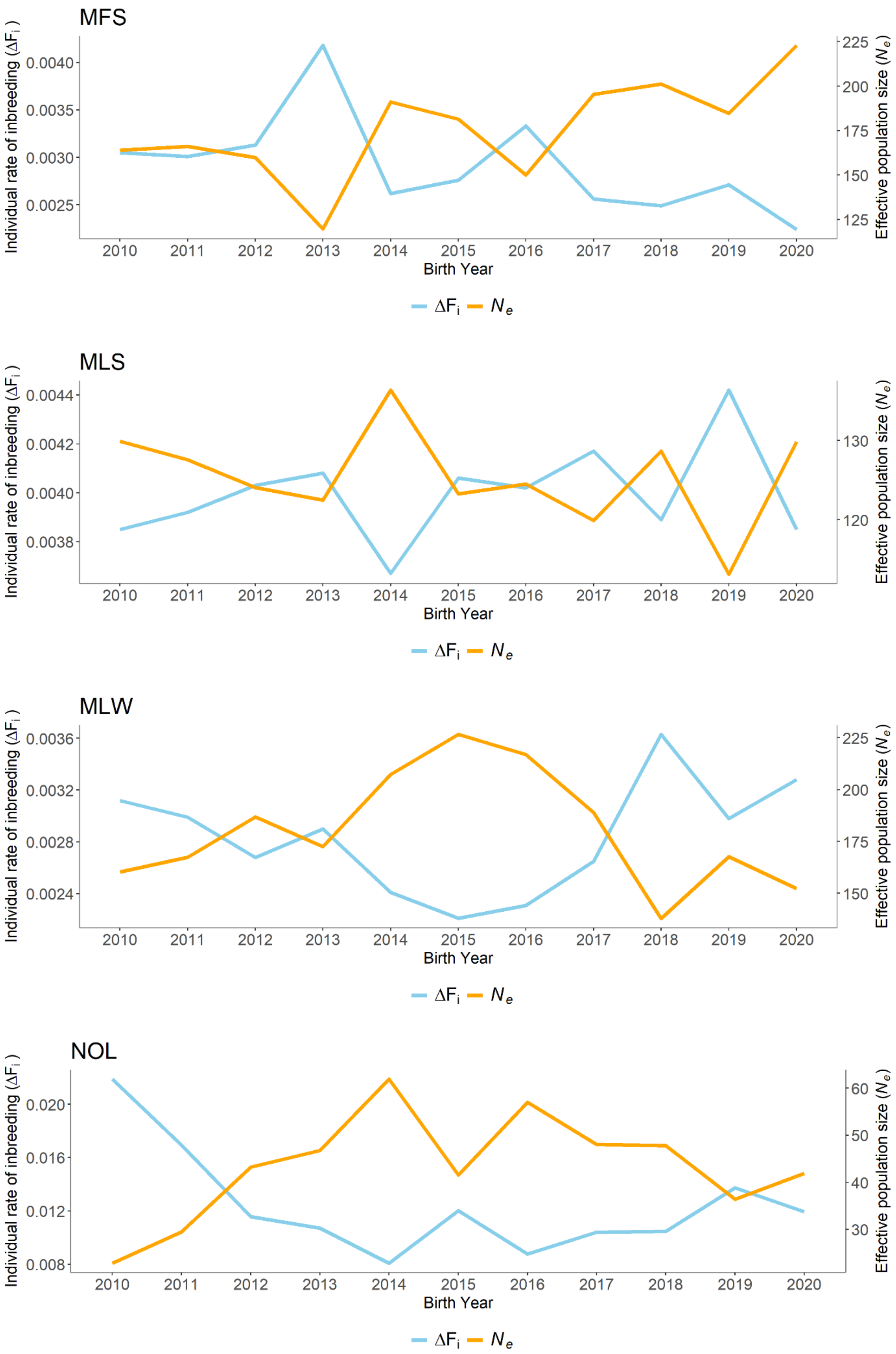
MFS
Individual rate of inbreeding (ΔF_i)
0.0040
0.0035
0.0030
0.0025
Effective population size (N_e)
225
200
175
150
125
2010
2011
2012
2013
2014
2015
2016
2017
2018
2019
2020
Birth Year
ΔF_i N_e
MLS
Individual rate of inbreeding (ΔF_i)
0.0044
0.0042
0.0040
0.0038
Effective population size (N_e)
130
120
2010
2011
2012
2013
2014
2015
2016
2017
2018
2019
2020
Birth Year
ΔF_i N_e
MLW
Individual rate of inbreeding (ΔF_i)
0.0036
0.0032
0.0028
0.0024
Effective population size (N_e)
225
200
175
150
2010
2011
2012
2013
2014
2015
2016
2017
2018
2019
2020
Birth Year
ΔF_i N_e
NOL
Individual rate of inbreeding (ΔF_i)
0.020
0.016
0.012
0.008
Effective population size (N_e)
60
50
40
30
2010
2011
2012
2013
2014
2015
2016
2017
2018
2019
2020
Birth Year
ΔF_i N_e

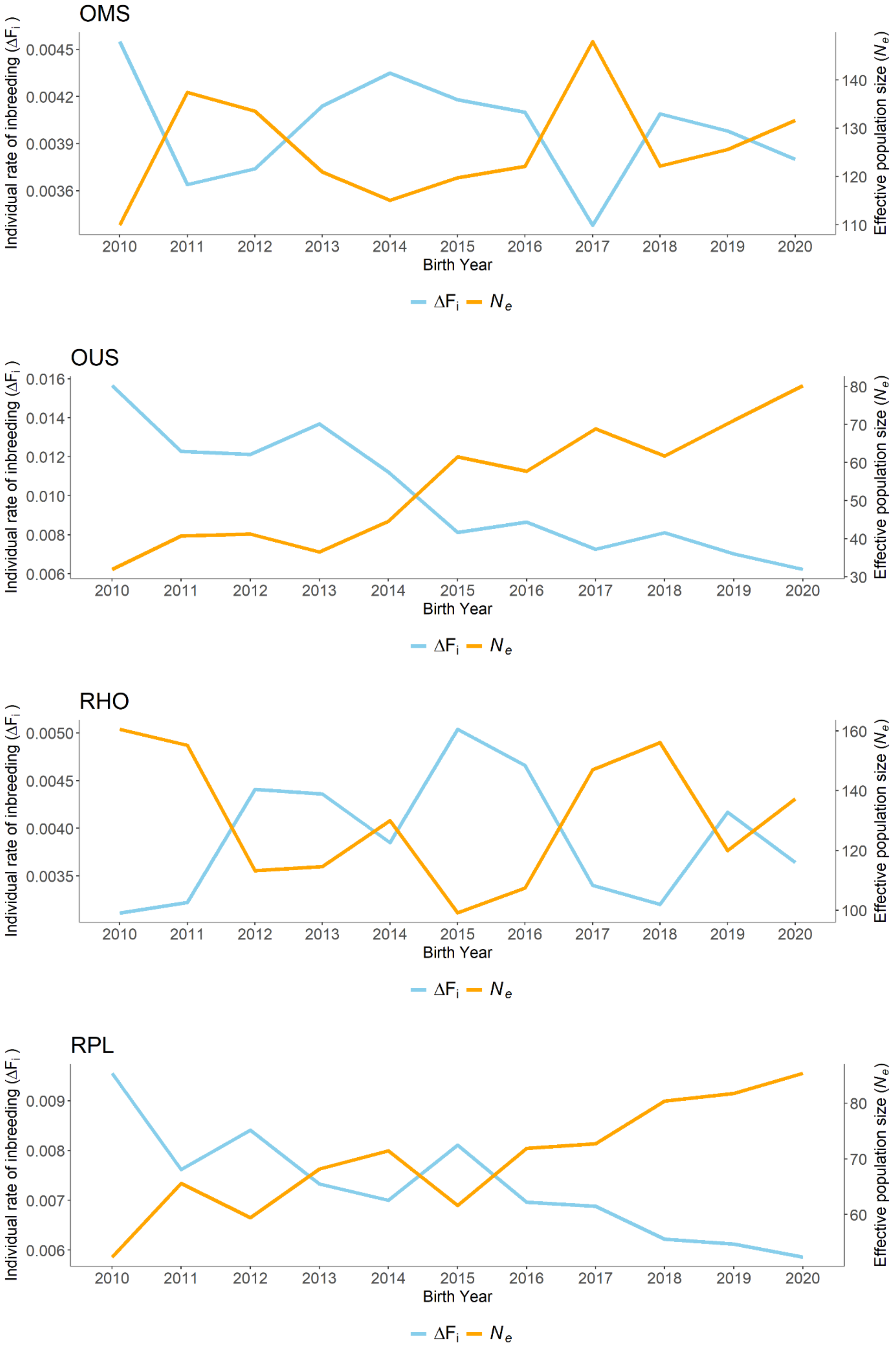

OMS
Individual rate of inbreeding (ΔFi)
0.0045
0.0042
0.0039
0.0036
Effective population size (Ne)
140
130
120
110
2010 2011 2012 2013 2014 2015 2016 2017 2018 2019 2020
Birth Year
ΔFi Ne
OUS
Individual rate of inbreeding (ΔFi)
0.016
0.014
0.012
0.010
0.008
0.006
Effective population size (Ne)
80
70
60
50
40
30
2010 2011 2012 2013 2014 2015 2016 2017 2018 2019 2020
Birth Year
ΔFi Ne
RHO
Individual rate of inbreeding (ΔFi)
0.0050
0.0045
0.0040
0.0035
Effective population size (Ne)
160
140
120
100
2010 2011 2012 2013 2014 2015 2016 2017 2018 2019 2020
Birth Year
ΔFi Ne
RPL
Individual rate of inbreeding (ΔFi)
0.009
0.008
0.007
0.006
Effective population size (Ne)
80
70
60
2010 2011 2012 2013 2014 2015 2016 2017 2018 2019 2020
Birth Year
ΔFi Ne

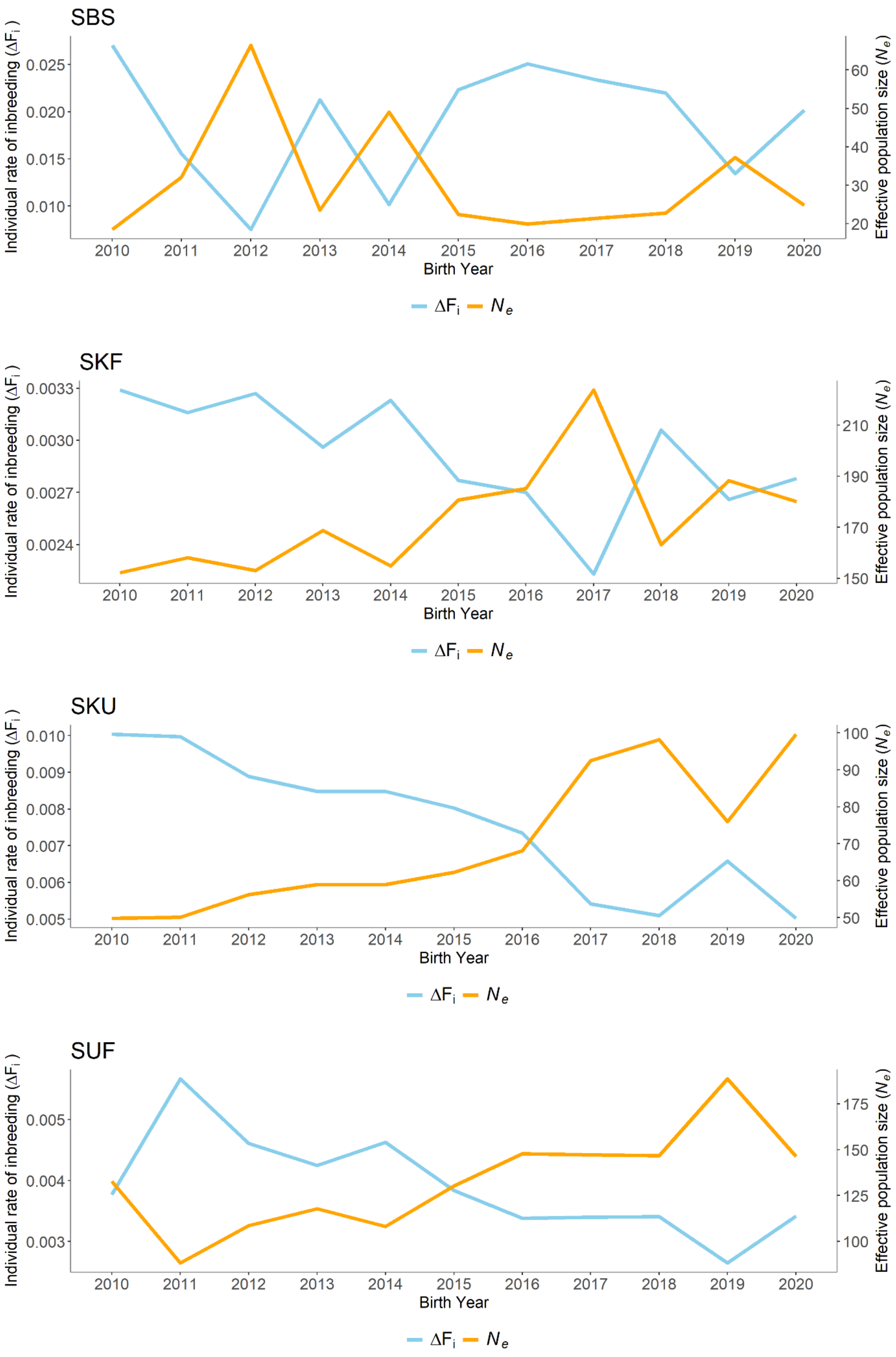
SBS
Individual rate of inbreeding (ΔFi)
0.025
0.020
0.015
0.010
Effective population size (Ne)
60
50
40
30
20
2010 2011 2012 2013 2014 2015 2016 2017 2018 2019 2020
Birth Year
ΔFi Ne
SKF
Individual rate of inbreeding (ΔFi)
0.0033
0.0030
0.0027
0.0024
Effective population size (Ne)
210
190
170
150
2010 2011 2012 2013 2014 2015 2016 2017 2018 2019 2020
Birth Year
ΔFi Ne
SKU
Individual rate of inbreeding (ΔFi)
0.010
0.009
0.008
0.007
0.006
0.005
Effective population size (Ne)
100
90
80
70
60
50
2010 2011 2012 2013 2014 2015 2016 2017 2018 2019 2020
Birth Year
ΔFi Ne
SUF
Individual rate of inbreeding (ΔFi)
0.005
0.004
0.003
Effective population size (Ne)
175
150
125
100
2010 2011 2012 2013 2014 2015 2016 2017 2018 2019 2020
Birth Year
ΔFi Ne

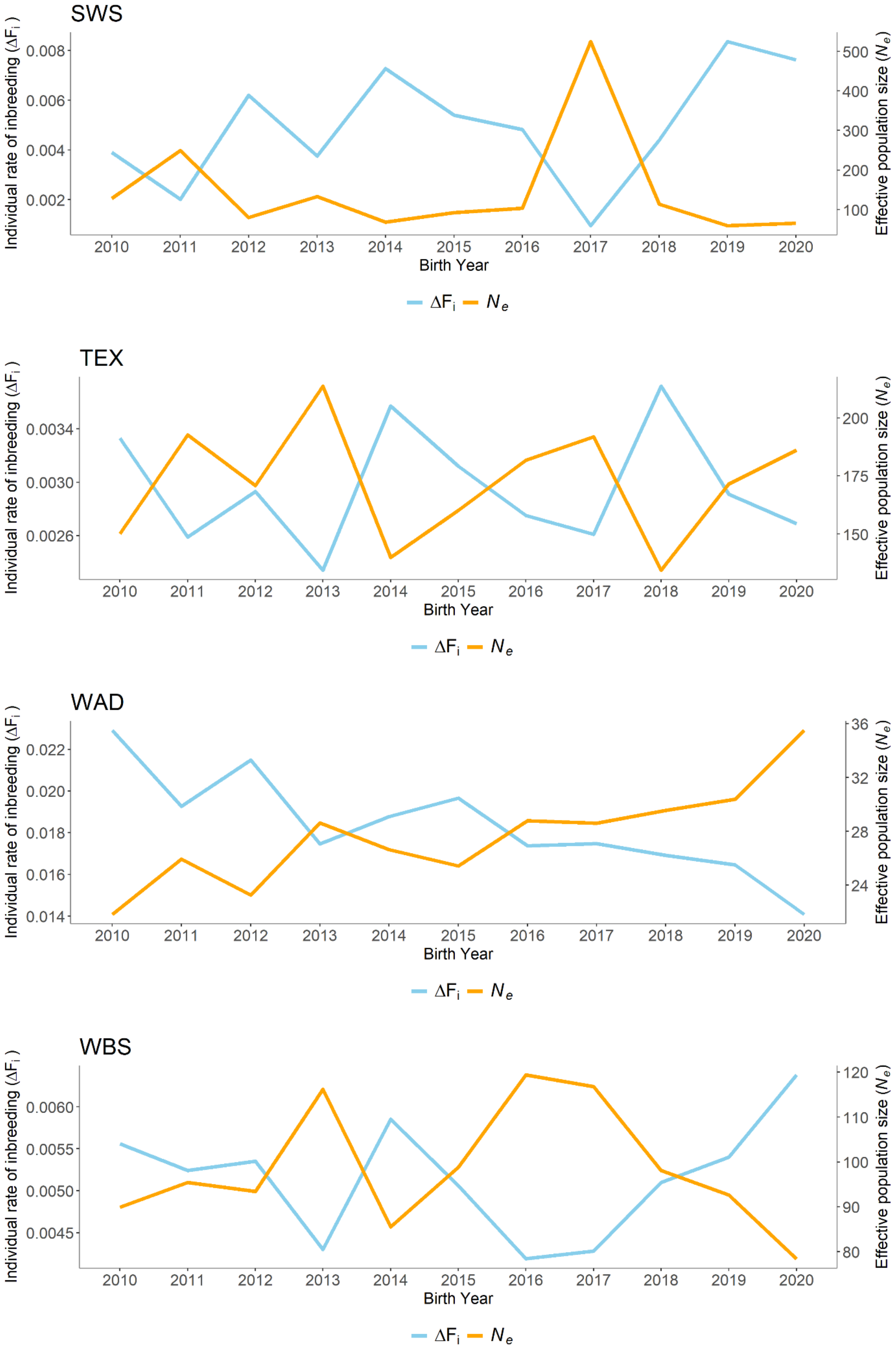
SWS
Individual rate of inbreeding (ΔFi)
0.008
0.006
0.004
0.002
Effective population size (Ne)
500
400
300
200
100
2010 2011 2012 2013 2014 2015 2016 2017 2018 2019 2020
Birth Year
ΔFi Ne
TEX
Individual rate of inbreeding (ΔFi)
0.0034
0.0030
0.0026
Effective population size (Ne)
200
175
150
2010 2011 2012 2013 2014 2015 2016 2017 2018 2019 2020
Birth Year
ΔFi Ne
WAD
Individual rate of inbreeding (ΔFi)
0.022
0.020
0.018
0.016
0.014
Effective population size (Ne)
36
32
28
24
2010 2011 2012 2013 2014 2015 2016 2017 2018 2019 2020
Birth Year
ΔFi Ne
WBS
Individual rate of inbreeding (ΔFi)
0.0060
0.0055
0.0050
0.0045
Effective population size (Ne)
120
110
100
90
80
2010 2011 2012 2013 2014 2015 2016 2017 2018 2019 2020
Birth Year
ΔFi Ne

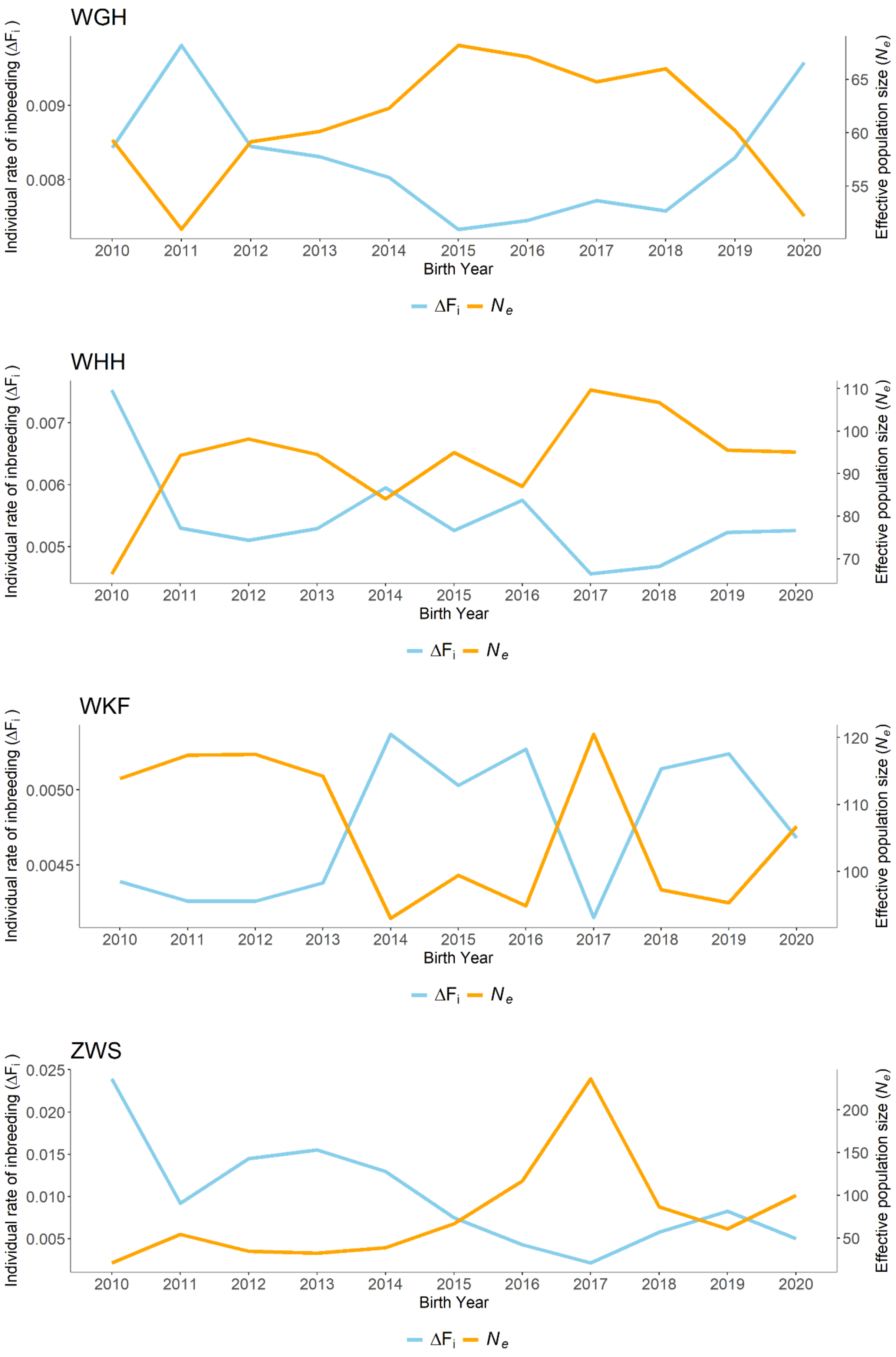

WGH
Individual rate of inbreeding (ΔFi)
0.009
0.008
Effective population size (Ne)
65
60
55
2010 2011 2012 2013 2014 2015 2016 2017 2018 2019 2020
Birth Year
ΔFi Ne
WHH
Individual rate of inbreeding (ΔFi)
0.007
0.006
0.005
Effective population size (Ne)
110
100
90
80
70
2010 2011 2012 2013 2014 2015 2016 2017 2018 2019 2020
Birth Year
ΔFi Ne
WKF
Individual rate of inbreeding (ΔFi)
0.0050
0.0045
Effective population size (Ne)
120
110
100
2010 2011 2012 2013 2014 2015 2016 2017 2018 2019 2020
Birth Year
ΔFi Ne
ZWS
Individual rate of inbreeding (ΔFi)
0.025
0.020
0.015
0.010
0.005
Effective population size (Ne)
200
150
100
50
2010 2011 2012 2013 2014 2015 2016 2017 2018 2019 2020
Birth Year
ΔFi Ne
