# Supplementary material for: Genetic Diversity and Trends of Ancestral and New Inbreeding in German Sheep Breeds by Pedigree Data
Source: Animals (Basel). 2023 Feb 10;13(4):623. doi: 10.3390/ani13040623 (PMC9951766; doi:10.3390/ani13040623)
Supplement: Supplementary file 1 [file animals-13-00623-s001.zip › Figure S5-Ancestral inbreeding.pdf]

Figure S5. Ancestral inbreeding coefficients according to Baumung (AHC), Ballou (F_{a_Bal}), Kalinowski (F_{a_Kal}) and the new inbreeding coefficient according to Kalinowski (F_{a_New}) for the birth years 2010 to 2020 for each of the 35 sheep breeds in Germany.

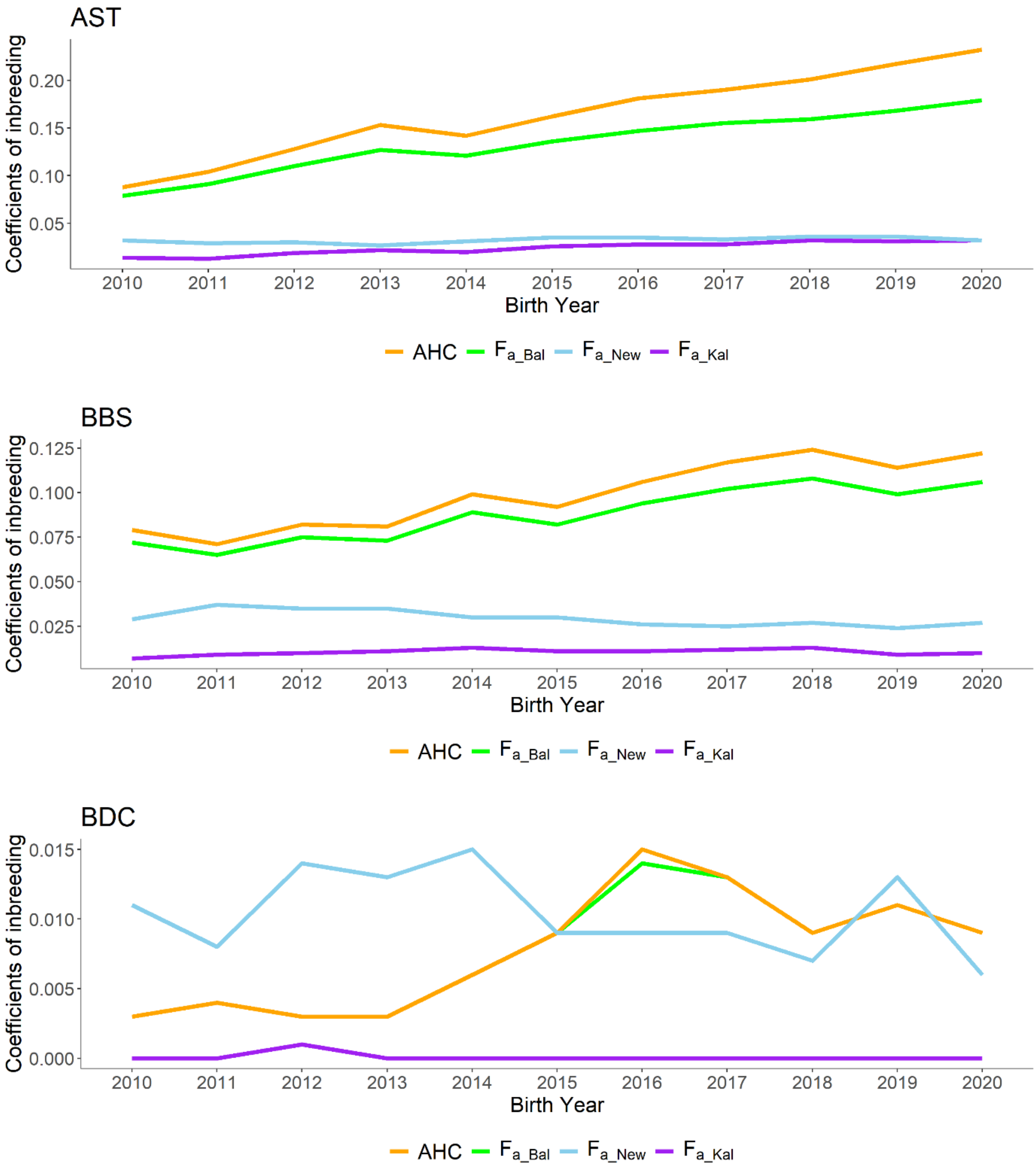

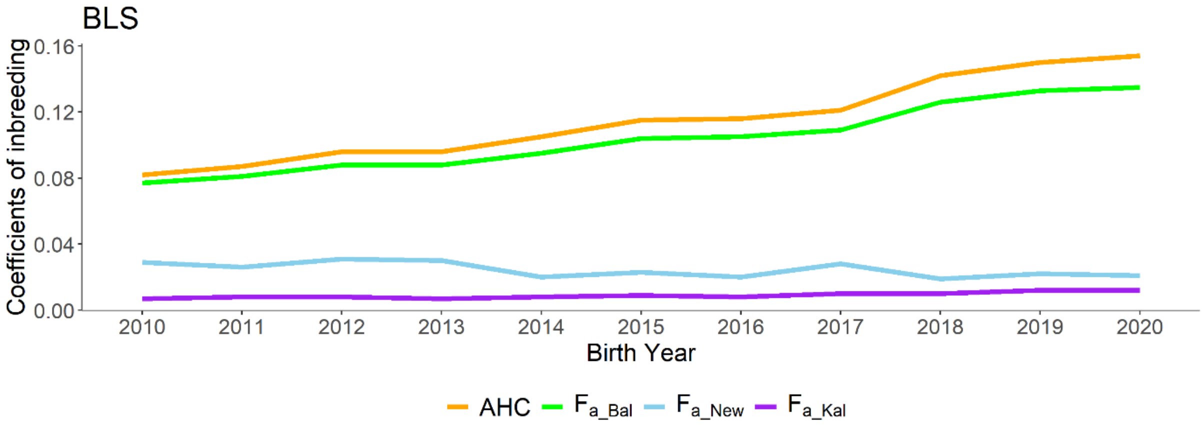

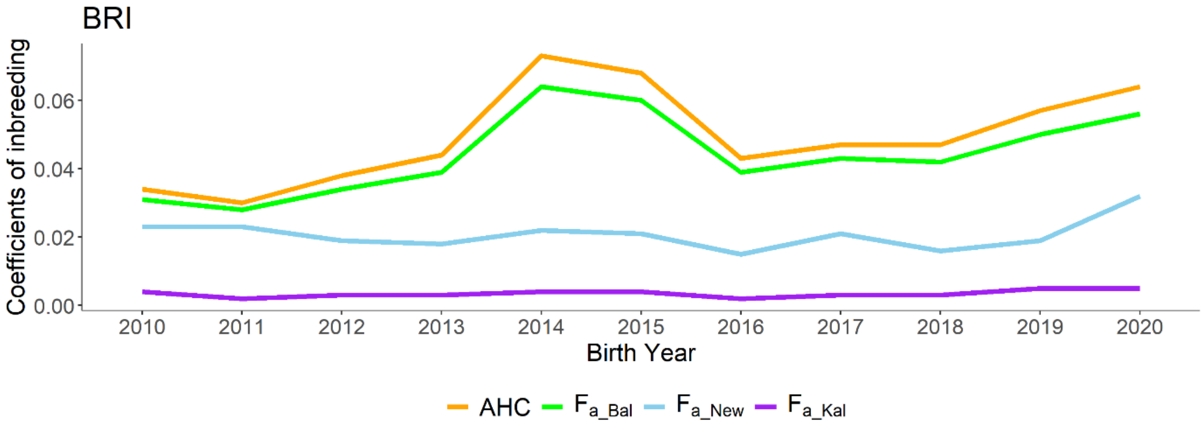

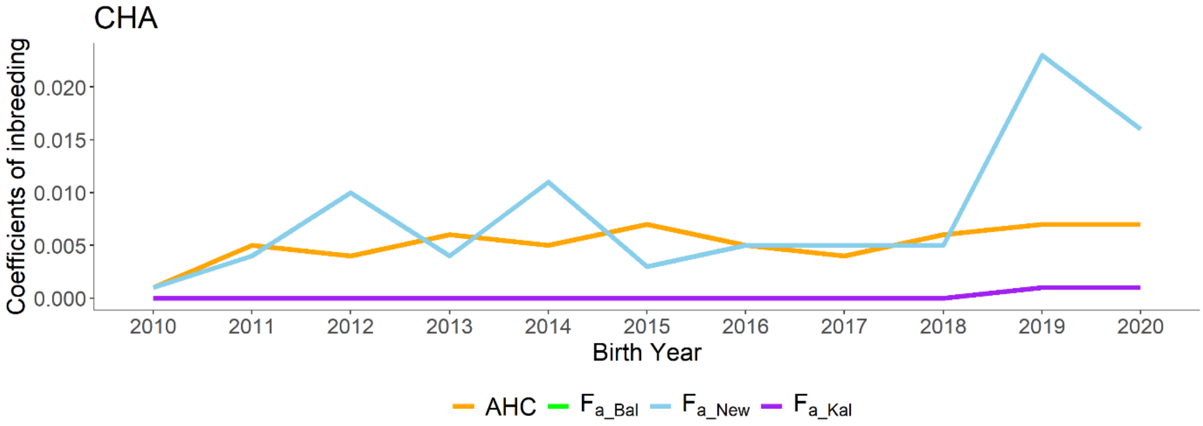

CHA, AHC and F_{a_Bal} showing exactly the same values.

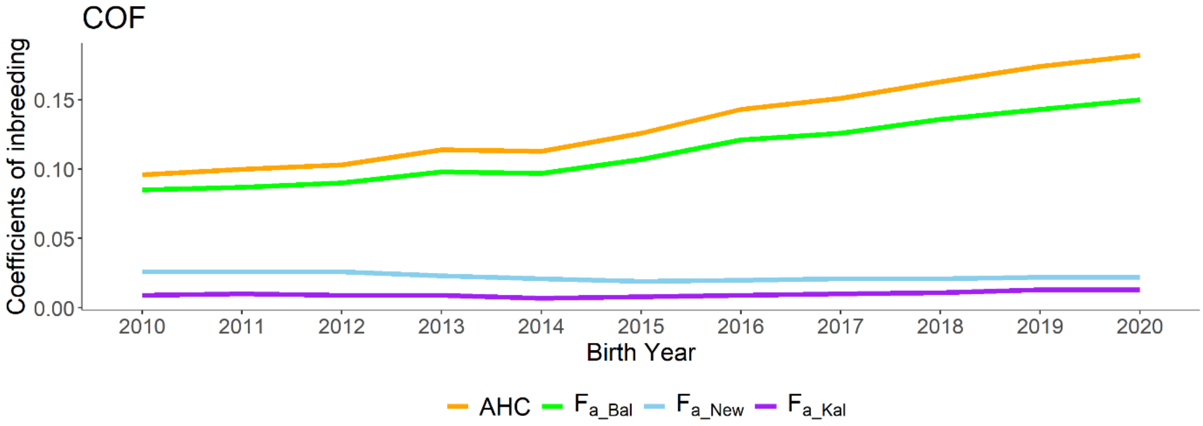

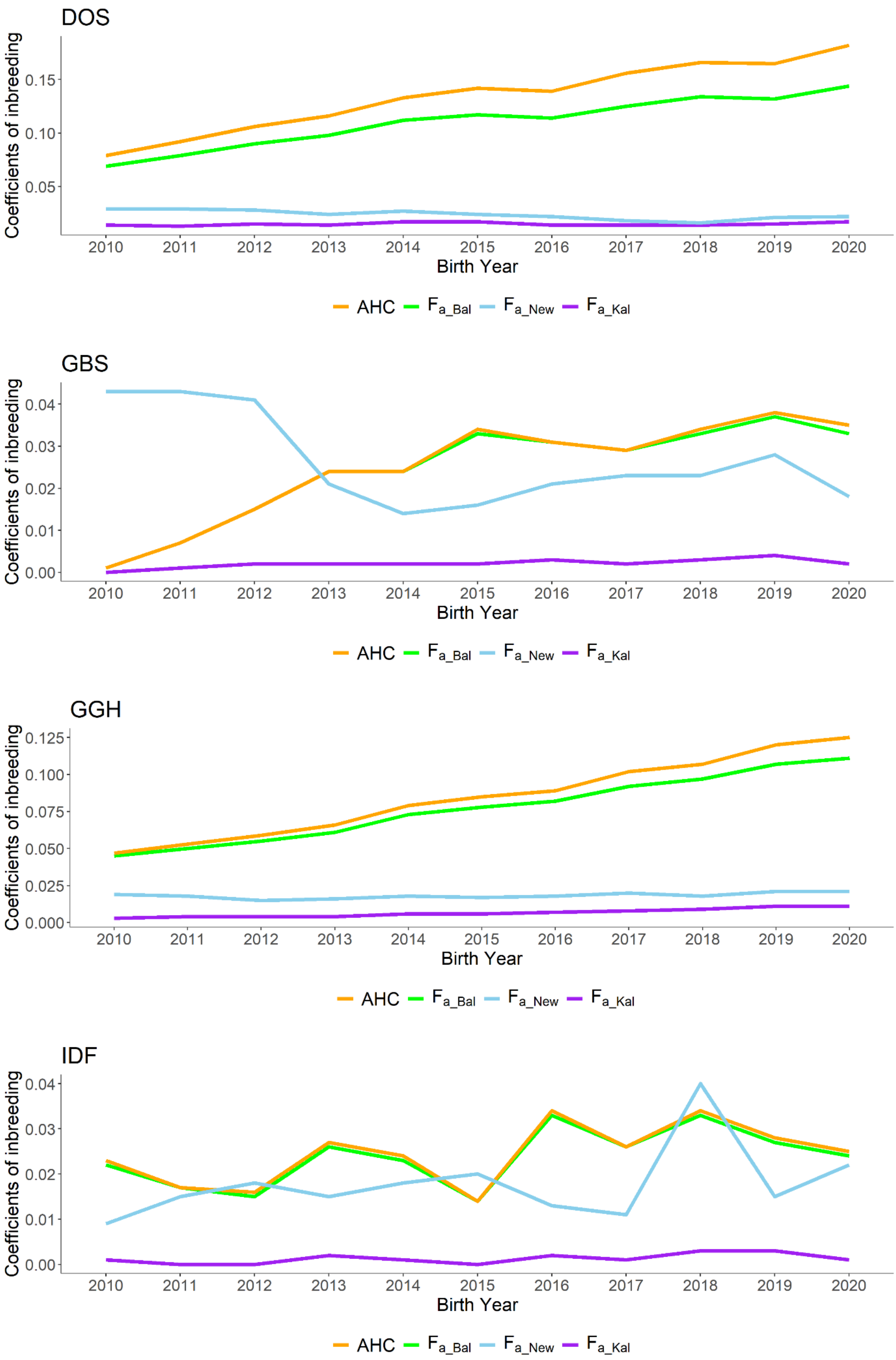

DOS
Coefficients of inbreeding
0.05
0.10
0.15
2010
2011
2012
2013
2014
2015
2016
2017
2018
2019
2020
Birth Year
AHC
F_{a_Bal}
F_{a_New}
F_{a_Kal}
GBS
Coefficients of inbreeding
0.00
0.01
0.02
0.03
0.04
2010
2011
2012
2013
2014
2015
2016
2017
2018
2019
2020
Birth Year
AHC
F_{a_Bal}
F_{a_New}
F_{a_Kal}
GGH
Coefficients of inbreeding
0.000
0.025
0.050
0.075
0.100
0.125
2010
2011
2012
2013
2014
2015
2016
2017
2018
2019
2020
Birth Year
AHC
F_{a_Bal}
F_{a_New}
F_{a_Kal}
IDF
Coefficients of inbreeding
0.00
0.01
0.02
0.03
0.04
2010
2011
2012
2013
2014
2015
2016
2017
2018
2019
2020
Birth Year
AHC
F_{a_Bal}
F_{a_New}
F_{a_Kal}

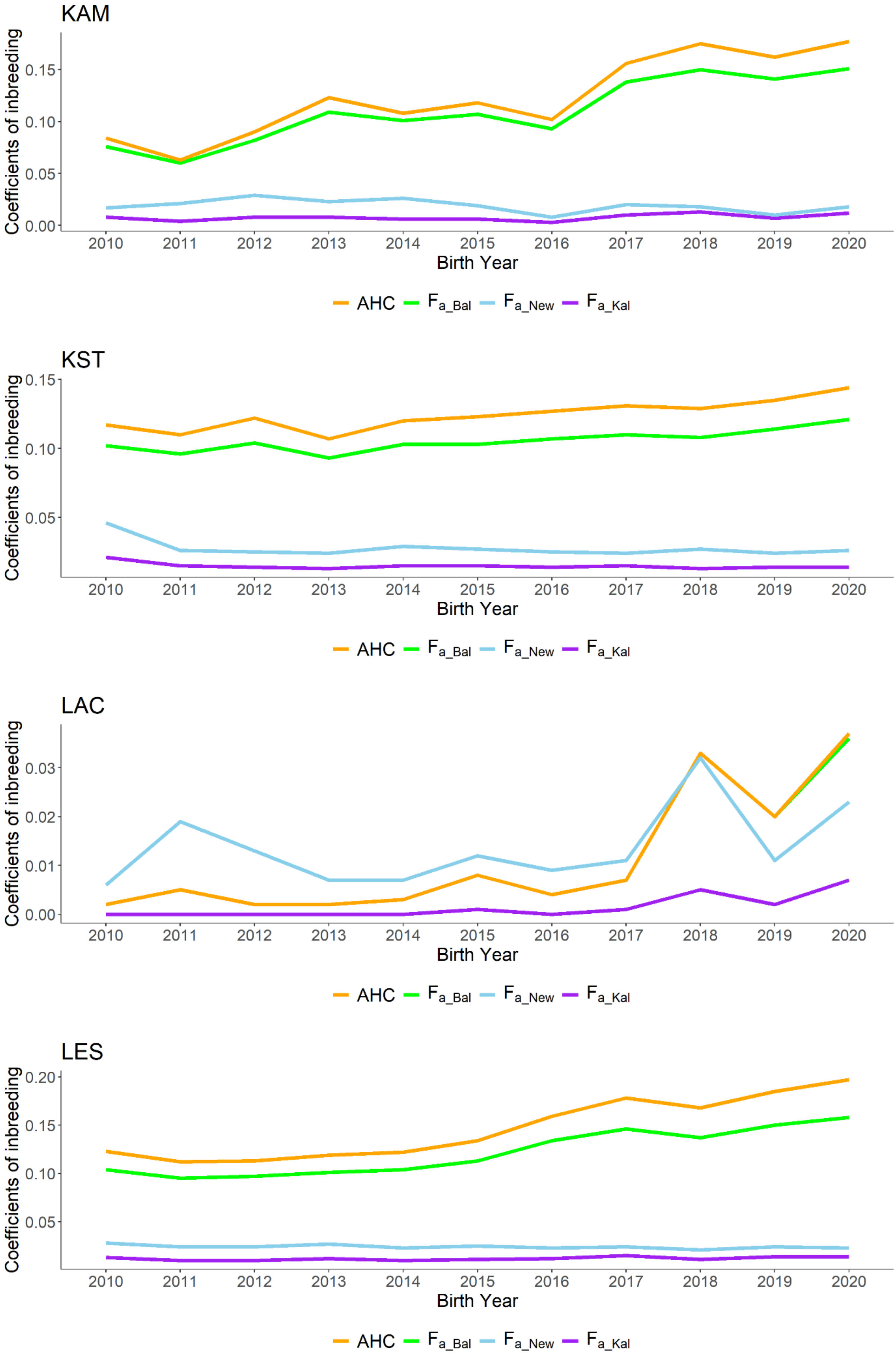
KAM
Coefficients of inbreeding
0.00
0.05
0.10
0.15
2010
2011
2012
2013
2014
2015
2016
2017
2018
2019
2020
Birth Year
AHC
F_{a_Bal}
F_{a_New}
F_{a_Kal}
KST
Coefficients of inbreeding
0.05
0.10
0.15
2010
2011
2012
2013
2014
2015
2016
2017
2018
2019
2020
Birth Year
AHC
F_{a_Bal}
F_{a_New}
F_{a_Kal}
LAC
Coefficients of inbreeding
0.00
0.01
0.02
0.03
2010
2011
2012
2013
2014
2015
2016
2017
2018
2019
2020
Birth Year
AHC
F_{a_Bal}
F_{a_New}
F_{a_Kal}
LES
Coefficients of inbreeding
0.05
0.10
0.15
0.20
2010
2011
2012
2013
2014
2015
2016
2017
2018
2019
2020
Birth Year
AHC
F_{a_Bal}
F_{a_New}
F_{a_Kal}

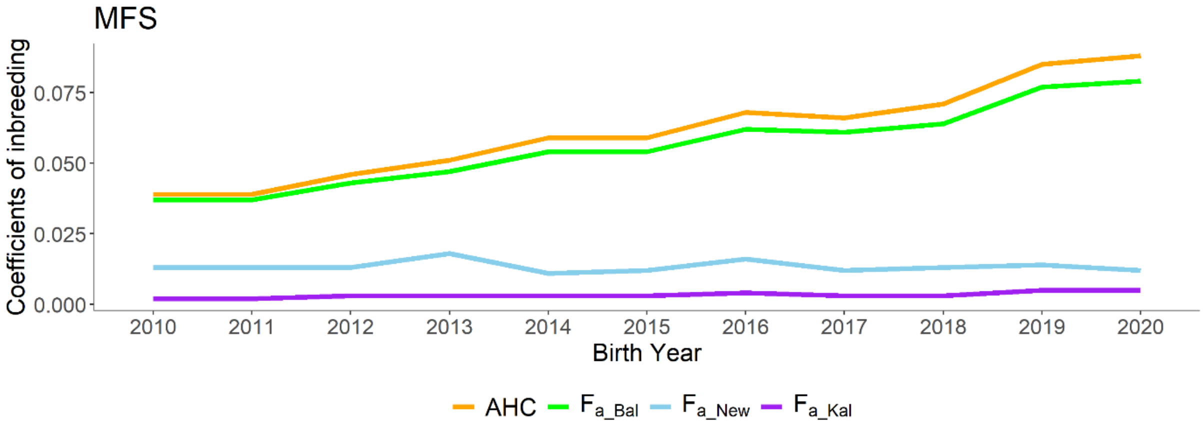
MFS
Coefficients of inbreeding
0.000
0.025
0.050
0.075
2010
2011
2012
2013
2014
2015
2016
2017
2018
2019
2020
Birth Year
AHC
F_{a_Bal}
F_{a_New}
F_{a_Kal}

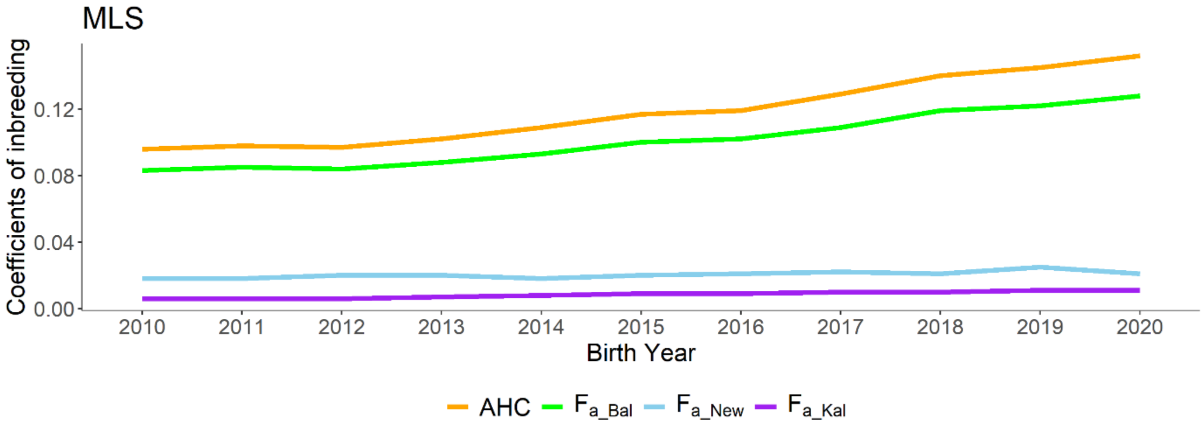
MLS
Coefficients of inbreeding
0.00
0.04
0.08
0.12
2010
2011
2012
2013
2014
2015
2016
2017
2018
2019
2020
Birth Year
AHC
F_{a_Bal}
F_{a_New}
F_{a_Kal}

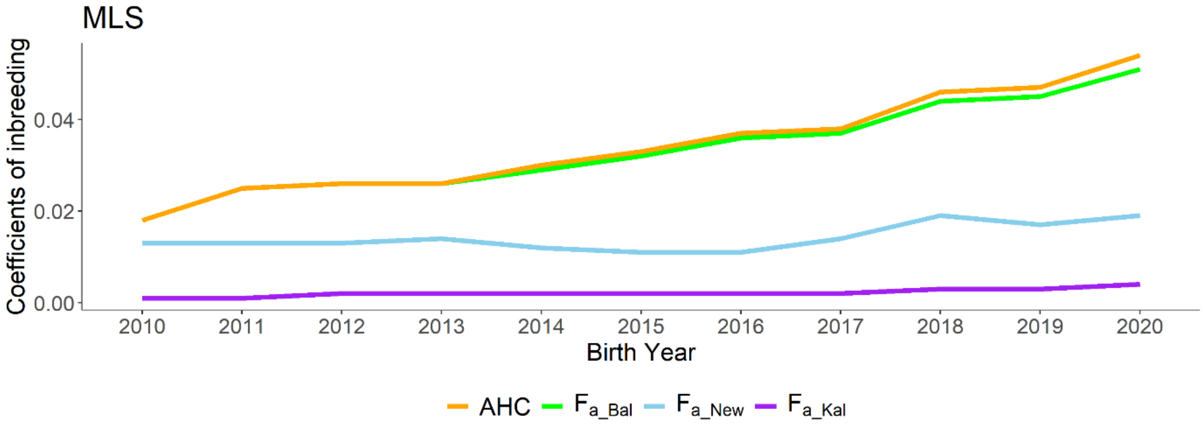
MLS
Coefficients of inbreeding
0.00
0.02
0.04
2010
2011
2012
2013
2014
2015
2016
2017
2018
2019
2020
Birth Year
AHC
F_{a_Bal}
F_{a_New}
F_{a_Kal}

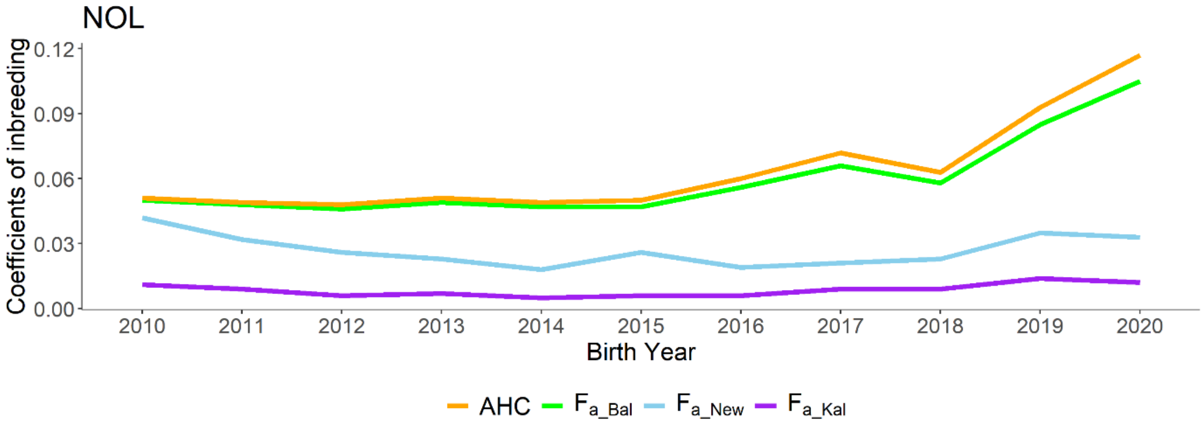
NOL
Coefficients of inbreeding
0.00
0.03
0.06
0.09
0.12
2010
2011
2012
2013
2014
2015
2016
2017
2018
2019
2020
Birth Year
AHC
F_{a_Bal}
F_{a_New}
F_{a_Kal}

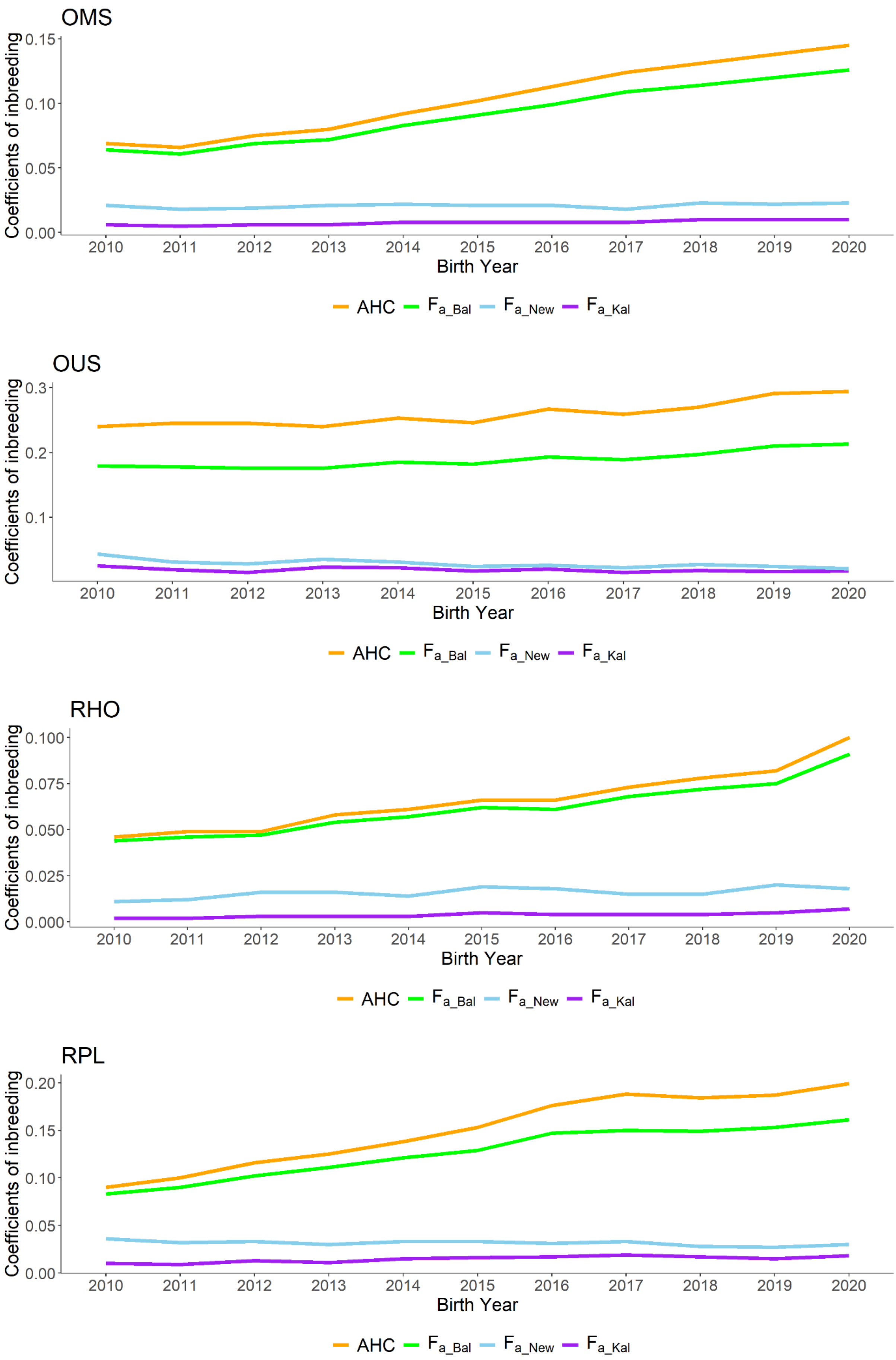
OMS
Coefficients of inbreeding
0.15
0.10
0.05
0.00
2010
2011
2012
2013
2014
2015
2016
2017
2018
2019
2020
Birth Year
AHC
Fa_Bal
Fa_New
Fa_Kal
OUS
0.3
0.2
0.1
RHO
0.100
0.075
0.050
0.025
0.000
RPL
0.20
0.15
0.10
0.05
0.00

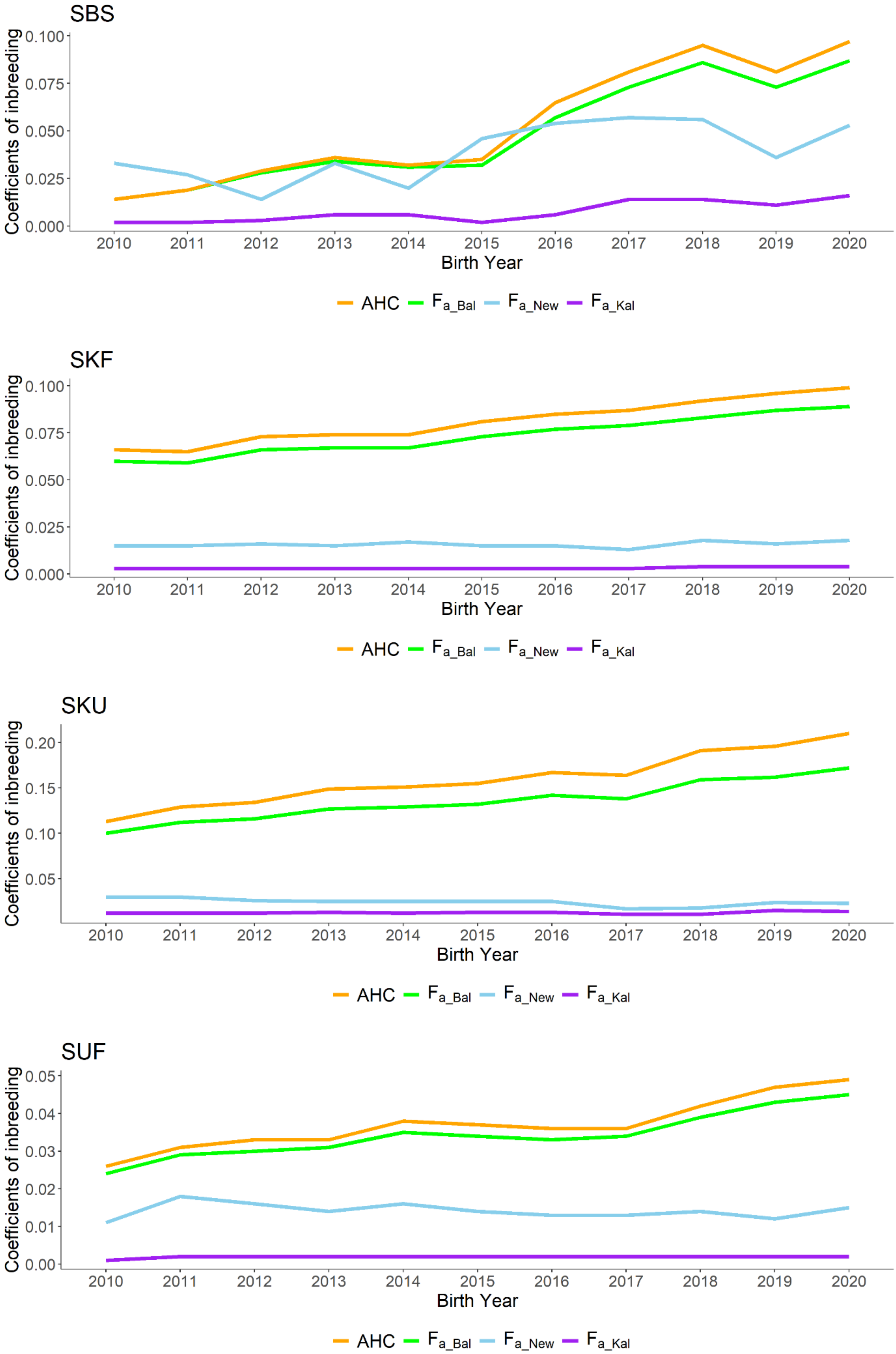
SBS
Coefficients of inbreeding
0.100
0.075
0.050
0.025
0.000
2010 2011 2012 2013 2014 2015 2016 2017 2018 2019 2020
Birth Year
AHC F_{a_Bal} F_{a_New} F_{a_Kal}
SKF
Coefficients of inbreeding
0.100
0.075
0.050
0.025
0.000
2010 2011 2012 2013 2014 2015 2016 2017 2018 2019 2020
Birth Year
AHC F_{a_Bal} F_{a_New} F_{a_Kal}
SKU
Coefficients of inbreeding
0.20
0.15
0.10
0.05
2010 2011 2012 2013 2014 2015 2016 2017 2018 2019 2020
Birth Year
AHC F_{a_Bal} F_{a_New} F_{a_Kal}
SUF
Coefficients of inbreeding
0.05
0.04
0.03
0.02
0.01
0.00
2010 2011 2012 2013 2014 2015 2016 2017 2018 2019 2020
Birth Year
AHC F_{a_Bal} F_{a_New} F_{a_Kal}

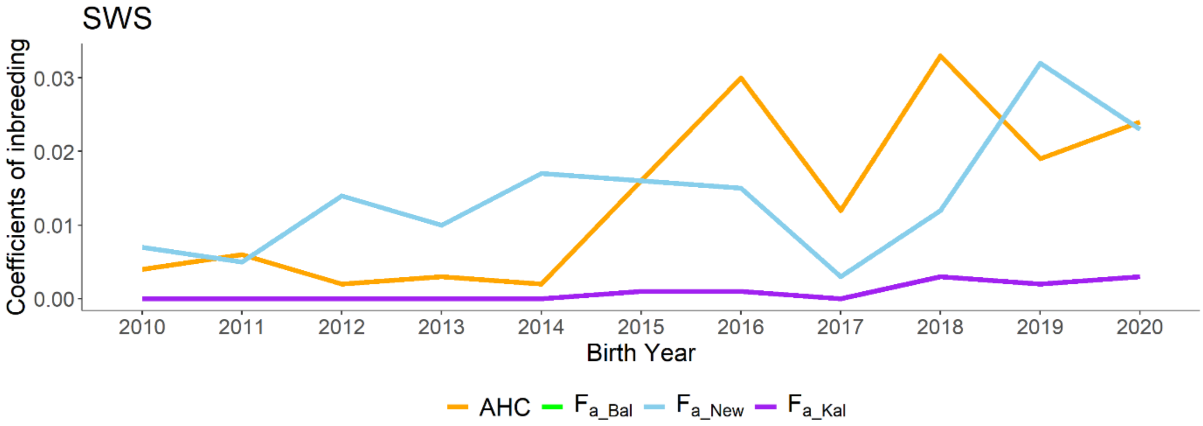

SWS, AHC and F_{a_Bal} showing exactly the same values.

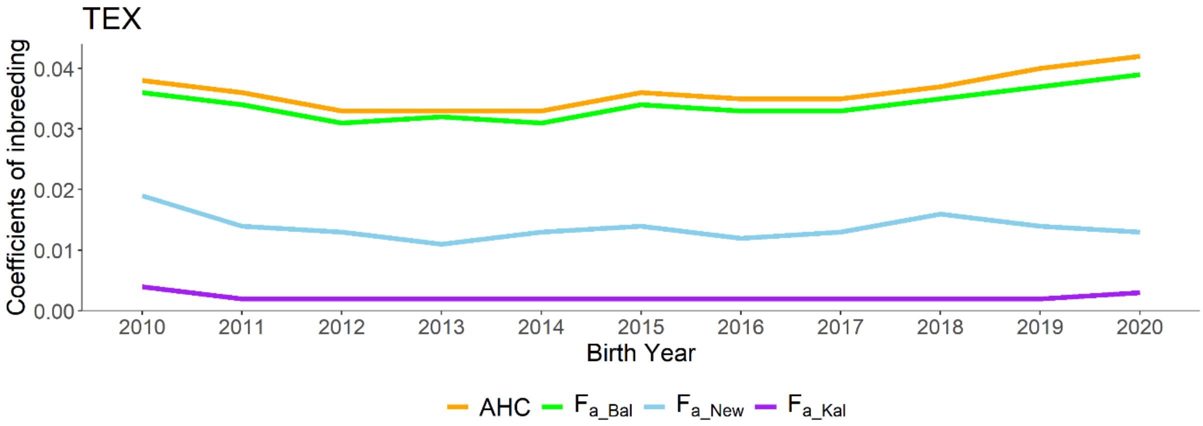

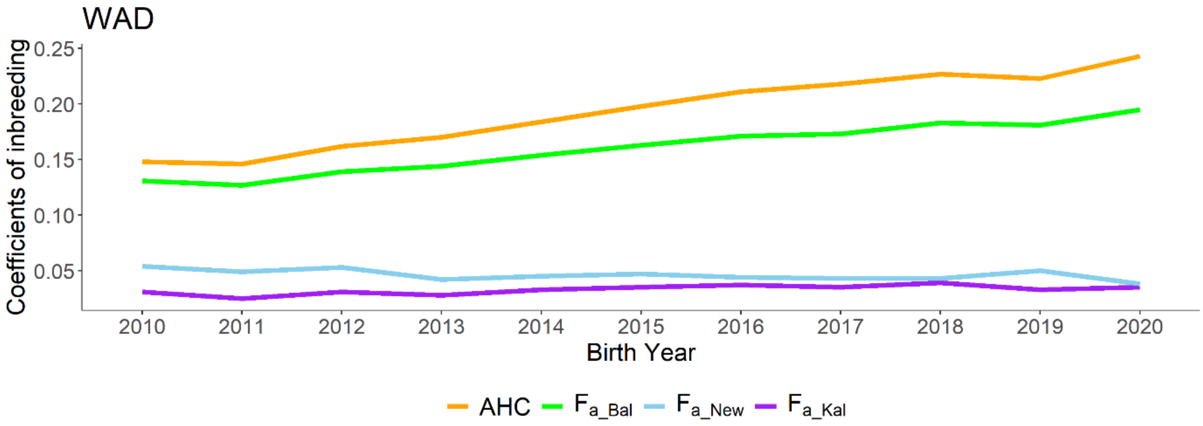

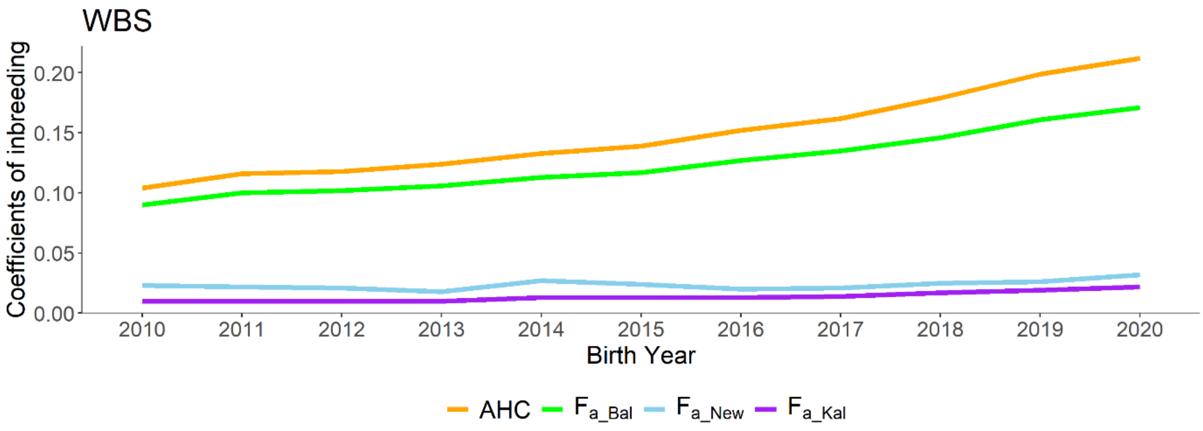

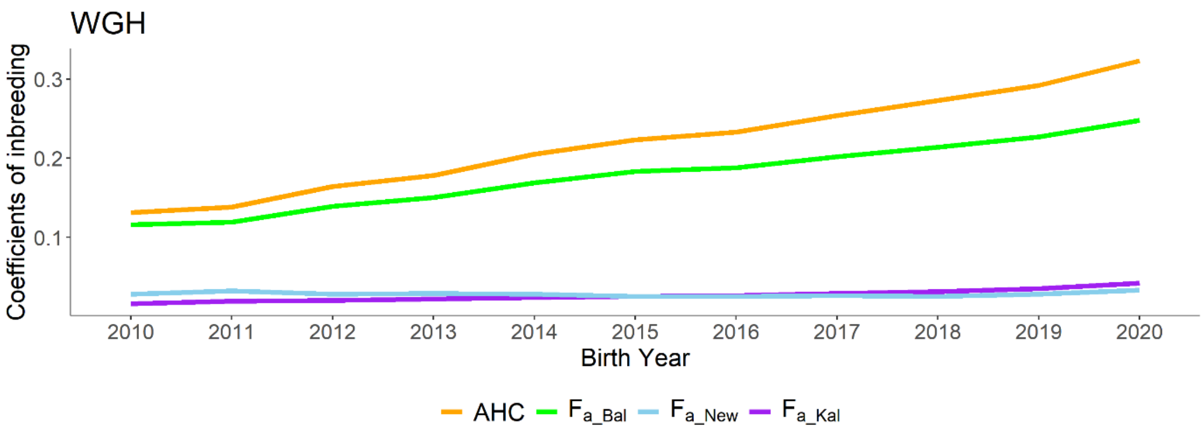
WGH
Coefficients of inbreeding
0.1
0.2
0.3
2010
2011
2012
2013
2014
2015
2016
2017
2018
2019
2020
Birth Year
AHC
F_{a_Bal}
F_{a_New}
F_{a_Kal}

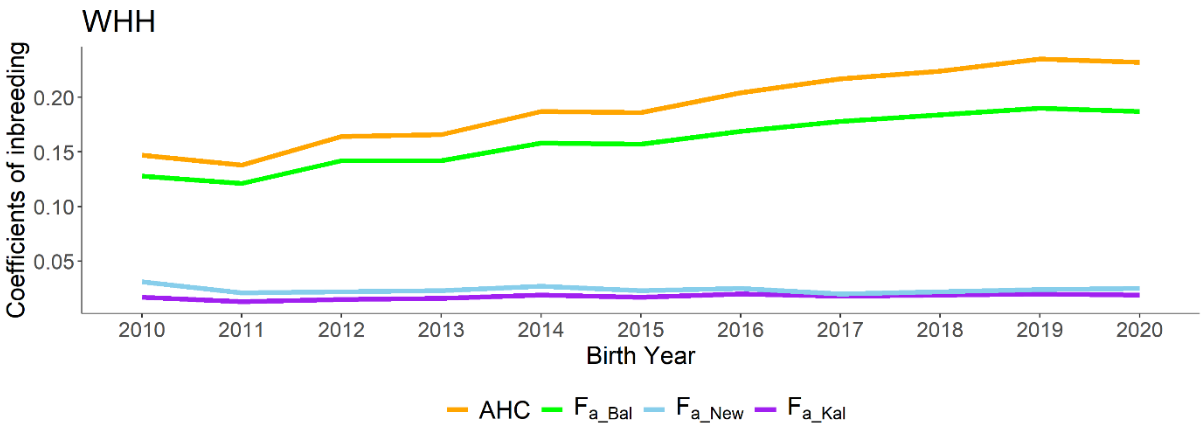
WHH
Coefficients of inbreeding
0.05
0.10
0.15
0.20
2010
2011
2012
2013
2014
2015
2016
2017
2018
2019
2020
Birth Year
AHC
F_{a_Bal}
F_{a_New}
F_{a_Kal}

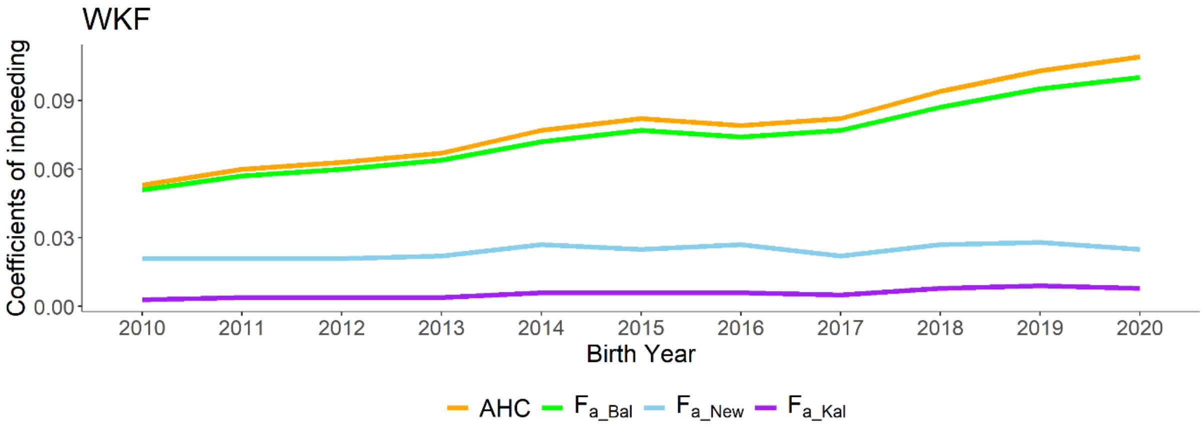
WKF
Coefficients of inbreeding
0.00
0.03
0.06
0.09
2010
2011
2012
2013
2014
2015
2016
2017
2018
2019
2020
Birth Year
AHC
F_{a_Bal}
F_{a_New}
F_{a_Kal}

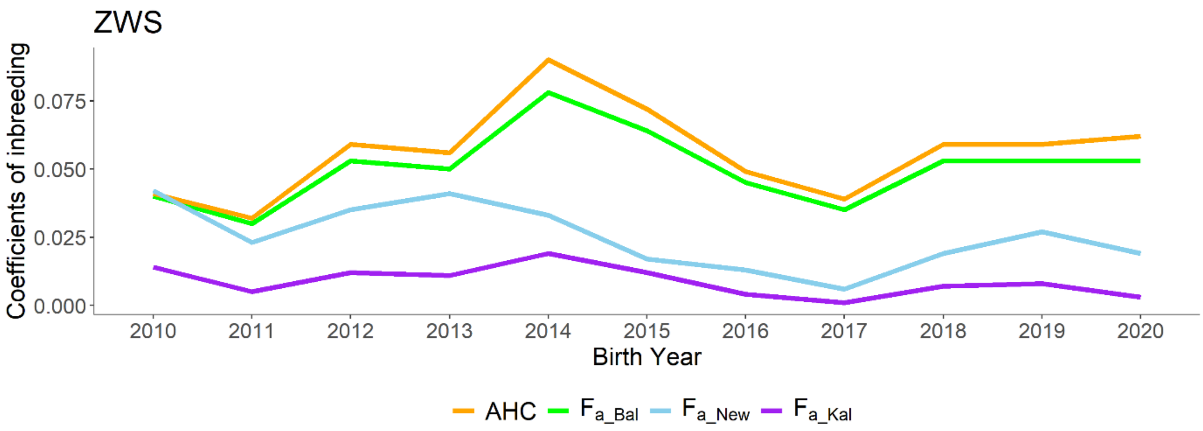
ZWS
Coefficients of inbreeding
0.000
0.025
0.050
0.075
2010
2011
2012
2013
2014
2015
2016
2017
2018
2019
2020
Birth Year
AHC
F_{a_Bal}
F_{a_New}
F_{a_Kal}
