# Supplementary material for: Genetic Diversity and Trends of Ancestral and New Inbreeding in German Sheep Breeds by Pedigree Data
Source: Animals (Basel). 2023 Feb 10;13(4):623. doi: 10.3390/ani13040623 (PMC9951766; doi:10.3390/ani13040623)
Supplement: Supplementary file 1 [file animals-13-00623-s001.zip › Table S1-S8.pdf]

Table S1. Pedigree analysis for measures of genetic diversity for 35 sheep breeds in Germany and **a**ncestors explaining 30/50/70/90/95% of the gene pool (30%, 50%, 70%, 90%, 95%).

Breed	f_e/f	f_a/f_e	f_g/f_e	30%	50%	70%	90%	95%
AST	0.084	0.707	0.446	2	6	14	54	101
BBS	0.114	0.601	0.280	7	20	50	185	288
BDC	0.264	0.576	0.355	11	26	60	162	224
BLS	0.065	0.607	0.260	5	14	34	121	267
BRI	0.149	0.608	0.327	8	23	62	236	342
CHA	0.145	0.539	0.429	11	36	96	331	487
COF	0.063	0.432	0.180	10	30	89	548	1000
DOS	0.030	0.690	0.434	3	13	43	193	368
GBS	0.167	0.950	0.598	6	16	36	99	141
GGH	0.049	0.383	0.165	9	27	96	893	1000
IDF	0.202	0.532	0.351	12	26	53	134	190
KAM	0.166	0.756	0.387	5	12	24	69	98
KST	0.133	0.555	0.341	4	10	19	61	115
LAC	0.132	0.627	0.411	5	12	29	129	197
LES	0.077	0.540	0.237	8	19	42	140	337
MFS	0.038	0.281	0.131	12	37	146	1000	1000
MLS	0.031	0.390	0.173	9	27	96	558	1000
MLW	0.056	0.558	0.245	9	19	43	132	281
NOL	0.057	0.878	0.514	5	16	54	245	396
OMS	0.096	0.405	0.166	14	35	91	403	769
OUS	0.091	0.770	0.442	6	16	37	137	224
RHO	0.051	0.662	0.270	17	48	120	657	1000
RPL	0.080	0.621	0.305	6	17	43	146	244
SBS	0.158	0.600	0.420	2	5	12	48	69
SKF	0.041	0.560	0.265	14	40	107	417	834
SKU	0.109	0.544	0.260	12	31	76	314	581
SUF	0.112	0.472	0.263	33	96	244	799	1000
SWS	0.296	0.458	0.303	4	12	30	70	95
TEX	0.114	0.564	0.325	26	71	161	443	680
WAD	0.086	0.727	0.374	4	10	23	85	156
WBS	0.082	0.460	0.177	5	14	38	237	446
WGH	0.073	0.500	0.222	3	8	21	113	253
WHH	0.043	0.614	0.218	5	13	31	193	478
WKF	0.130	0.509	0.234	9	20	39	107	175
ZWS	0.254	0.381	0.201	5	13	38	127	180

Table S2. Amount of genetic diversity accounting for the loss of genetic diversity resulting from genetic drift *(GD)* or unequal contribution of founders *(GD*)*, **l**osses due to drift in % ($Loss_{drift}$), losses due to unequal contributions of founders in % ($Loss_{founder}$) as relative effects for each of the 35 sheep breeds.

**Breed**	***GD***	***GD****	**1- *GD***	***GD*-GD***	**1-*GD****	**$Loss_{drift}$**	**$Loss_{founder}$**
AST	0.953	0.979	0.047	0.026	0.021	55.440	44.560
BBS	0.980	0.994	0.020	0.014	0.006	72.010	27.990
BDC	0.989	0.996	0.011	0.007	0.004	64.490	35.510
BLS	0.972	0.993	0.028	0.021	0.007	73.980	26.020
BRI	0.985	0.995	0.015	0.010	0.005	67.270	32.730
CHA	0.992	0.997	0.008	0.004	0.003	57.080	42.920
COF	0.984	0.997	0.016	0.013	0.003	82.010	17.990
DOS	0.972	0.988	0.028	0.016	0.012	56.590	43.410
GBS	0.982	0.989	0.018	0.007	0.011	40.200	59.800
GGH	0.981	0.997	0.019	0.016	0.003	83.480	16.520
IDF	0.990	0.996	0.010	0.007	0.004	64.850	35.150
KAM	0.972	0.989	0.028	0.017	0.011	61.330	38.670
KST	0.972	0.991	0.028	0.018	0.009	65.880	34.120
LAC	0.979	0.991	0.021	0.012	0.009	58.890	41.110
LES	0.980	0.995	0.020	0.015	0.005	76.260	23.740
MFS	0.988	0.998	0.012	0.010	0.002	86.890	13.110
MLS	0.983	0.997	0.017	0.014	0.003	82.730	17.270
MLW	0.981	0.995	0.019	0.014	0.005	75.510	24.490
NOL	0.979	0.989	0.021	0.010	0.011	48.580	51.420
OMS	0.988	0.998	0.012	0.010	0.002	83.390	16.610
OUS	0.980	0.991	0.020	0.011	0.009	55.750	44.250
RHO	0.990	0.997	0.010	0.007	0.003	72.960	27.040
RPL	0.978	0.993	0.022	0.015	0.007	69.530	30.470
SBS	0.953	0.980	0.047	0.027	0.020	58.040	41.960
SKF	0.990	0.997	0.010	0.007	0.003	73.470	26.530
SKU	0.988	0.997	0.012	0.009	0.003	74.040	25.960
SUF	0.996	0.999	0.004	0.003	0.001	73.710	26.290
SWS	0.976	0.993	0.024	0.017	0.007	69.710	30.290
TEX	0.995	0.999	0.004	0.003	0.001	67.450	32.550
WAD	0.967	0.988	0.033	0.021	0.012	62.590	37.410
WBS	0.969	0.994	0.031	0.026	0.006	82.330	17.670
WGH	0.954	0.990	0.046	0.036	0.010	77.760	22.240
WHH	0.965	0.992	0.035	0.027	0.008	78.200	21.800
WKF	0.981	0.996	0.019	0.014	0.004	76.560	23.440
ZWS	0.976	0.995	0.024	0.019	0.005	79.850	20.150

Table S3. Linear regression coefficients of the inbreeding coefficient for all (F), for inbred animals (F_{inbred}), individual rate of inbreeding (ΔF_i), realized effective population size (N_e), effective number of sires and dams (effective sires, effective dams), and effective number of sires and dams corrected for yearly trend of population size (effective sires corrected, effective dams corrected) within breed on birth year with their *p*-values testing difference to zero for each of the 35 sheep breeds.

Breed	F	P-value	F_{inbred}	P-value	ΔF_i	P-value	N_e	P-value
AST	0.00257182	<0.0001	-0.00030000	0.8064	-0.00017255	0.4103	0.3785000	0.9115
BBS	-0.00080000	0.1479	-0.00225455	0.0662	-0.00049136	0.0195	3.5031909	0.3039
BDC	-0.00042000	0.4469	-0.00957273	<0.0001	-0.00043464	0.0386	15.3790273	<.0001
BLS	-0.00021818	0.6927	9.0909E-06	0.9941	-0.00021236	0.3110	4.7276091	0.1656
BRI	0.00025727	0.6412	-0.00273636	0.0259	-0.00036800	0.0796	3.3294273	0.3285
CHA	0.00121000	0.0290	-0.00644545	<0.0001	0.00015582	0.4571	-13.5814818	<0.0001
COF	-0.00017455	0.7518	-0.00091818	0.4533	-0.00032136	0.1256	4.9428636	0.1472
DOS	-0.00094909	0.0863	-0.00318182	0.0097	-0.00077927	0.0002	5.2498273	0.1238
GBS	-0.00178364	0.0013	-0.00489091	<0.0001	-0.00307918	<0.0001	2.8032636	0.4106
GGH	0.00113364	0.0407	0.00010909	0.9290	3.2727E-06	0.9875	-0.5076364	0.8815
IDF	0.00123455	0.0259	-0.00580909	<0.0001	0.00029791	0.1556	-2.3897182	0.4829
KAM	-0.00045727	0.4077	-0.00260909	0.0336	-0.00045600	0.0301	5.7300000	0.0931
KST	-0.00127727	0.0212	-0.00264545	0.0313	-0.00068955	0.0011	2.9791182	0.3819
LAC	0.00159909	0.0040	-0.00129091	0.2919	0.00015364	0.4634	-2.2760091	0.5040
LES	-5.0909E-05	0.9265	-0.00060909	0.6188	-0.00020727	0.3227	2.9423091	0.3878
MFS	0.00017091	0.7568	-0.00071818	0.5574	-8.8182E-05	0.6738	5.4839000	0.1080
MLS	0.00105364	0.0570	0.00090000	0.4623	1.9182E-05	0.9270	-0.5564636	0.8702
MLW	0.00082091	0.1376	0.00051818	0.6720	2.7364E-05	0.8961	-1.3051273	0.7015
NOL	-8.1818E-06	0.9882	-0.00234545	0.0560	-0.00059827	0.0045	1.2214455	0.7198
OMS	0.00074818	0.1759	0.00012727	0.9172	-2.8273E-05	0.8926	0.7979455	0.8147
OUS	-0.00211818	0.0001	-0.00377273	0.0022	-0.00086891	<0.0001	4.5640545	0.1807
RHO	0.00095727	0.0836	0.0011000	0.3691	1.5546E-05	0.9408	-0.7858273	0.8175
RPL	0.00021455	0.6975	0.0002000	0.8702	-0.00029055	0.1660	2.7436273	0.4206
SBS	0.00451909	<0.0001	-0.00177273	0.1481	0.00017755	0.3969	-1.0219636	0.7641
SKF	0.00029636	0.5914	0.00021818	0.8585	-6.5182E-05	0.7557	3.9129364	0.2509
SKU	-0.00086818	0.1164	-0.00085455	0.4852	-0.00052127	0.0133	5.0414909	0.1394
SUF	-0.00011182	0.8395	-0.00158182	0.1968	-0.00018527	0.3767	6.2154455	0.0686
SWS	0.00179273	0.0013	-0.00661818	<0.0001	0.00027727	0.1862	-1.3932636	0.6824
TEX	0.00019273	0.7270	-0.00025455	0.8352	1.5455E-06	0.9941	-0.1431273	0.9665
WAD	-0.00013909	0.8011	-9.0909E-06	0.9941	-0.00064055	0.0024	0.9736818	0.7749
WBS	0.00175636	0.0016	0.00158182	0.1968	2.0818E-05	0.9208	-0.1801636	0.9578
WGH	0.00211909	0.0001	0.00137273	0.2625	-4.2364E-05	0.8397	0.3264000	0.9236
WHH	0.00020545	0.7097	0.00030000	0.8064	-0.00013227	0.5278	1.8864091	0.5796
WKF	0.00117727	0.0336	0.00122727	0.3163	6.7727E-05	0.7464	-1.5443636	0.6502
ZWS	-0.00301909	<0.0001	-0.00638182	<0.0001	-0.00145382	<0.0001	9.6558091	0.0048
**Breed**	**Effective sires**	**P-value**	**Effective sires corrected**	**P-value**	**Effective dams**	**P-value**	**Effective dams corrected**	**P-value**
AST	1.3977620	0.0128	0.1376330	0.8768	25.523959	0.2284	8.052918	0.7752
BBS	0.6553785	0.2415	0.2351312	0.6975	0.155689	0.9941	3.263861	0.8651
BDC	-0.1929619	0.7299	0.0837152	0.8977	3.317003	0.8755	2.022670	0.9221
BLS	-0.2986127	0.5933	-0.8679244	0.1194	30.571048	0.1493	10.042333	0.5696
BRI	-0.0101136	0.9856	-0.0527645	0.9181	-0.728827	0.9725	-1.885221	0.9079
CHA	0.8955413	0.1098	0.9157122	0.0729	-8.206857	0.6982	-6.093119	0.7063
COF	2.3174354	<0.0001	3.1909834	0.0206	79.504973	0.0002	29.770881	0.4944
DOS	1.0511908	0.0607	-1.3963288	0.2491	78.407749	0.0002	25.284403	0.5107

GBS	1.4891248	0.0081	0.9014552	0.4946	15.310816	0.4696	4.519480	0.9141
GGH	-1.3195299	0.0188	-0.5834189	0.3274	-50.145884	0.0183	8.774497	0.6426
IDF	-0.2291243	0.6819	-0.0924949	0.9259	18.522311	0.3818	10.663401	0.7357
KAM	-0.0765157	0.8911	-0.1616196	0.7967	-8.636978	0.6833	1.808054	0.9277
KST	1.4702897	0.0089	-1.4514672	0.6292	33.223530	0.1172	-1.777999	0.9851
LAC	0.1214333	0.8280	0.0739809	0.8853	-0.921454	0.9653	-3.985348	0.8067
LES	0.6642898	0.2352	1.3028256	0.1652	62.628535	0.0033	11.517747	0.6988
MFS	-1.3033198	0.0202	-0.2063265	0.7900	-87.672303	<.0001	29.687739	0.2282
MLS	-2.8295127	<0.0001	-0.0608170	0.9615	-235.08740	<.0001	-13.023427	0.7445
MLW	-1.0504635	0.0609	-1.3942702	0.0331	-32.590520	0.1243	27.741858	0.1807
NOL	0.5586895	0.3179	1.2648623	0.0254	-6.997348	0.7410	1.112850	0.9504
OMS	-0.2301639	0.6805	0.3033274	0.6012	-8.857299	0.6756	16.362185	0.3749
OUS	1.4854559	0.0082	-1.5512434	0.2066	16.551681	0.4344	2.635116	0.9461
RHO	1.5928732	0.0046	1.3717309	0.0108	46.426011	0.0289	19.879423	0.2423
RPL	-2.0946461	0.0002	-0.0105269	0.9920	-90.884191	<.0001	0.303790	0.9927
SBS	0.0833519	0.8815	0.0713525	0.9188	3.830274	0.8564	1.801805	0.9353
SKF	-0.5337182	0.3400	1.9383048	0.0063	-64.561623	0.0025	-1.454652	0.9482
SKU	-1.3658783	0.0150	-0.9385917	0.1636	-38.057980	0.0729	-1.073040	0.9599
SUF	0.0482998	0.9311	-0.1801453	0.7725	-29.385841	0.1657	2.455903	0.9012
SWS	0.3924671	0.4827	0.6370204	0.3560	-4.157089	0.8443	-1.877261	0.9317
TEX	-3.9376294	<0.0001	-0.8419759	0.6393	-74.998110	0.0005	-2.358563	0.9670
WAD	0.7058926	0.2072	0.6469372	0.2348	-6.142446	0.7717	1.195780	0.9448
WBS	0.6680274	0.2326	0.5561513	0.2776	13.722460	0.5169	10.608552	0.5140
WGH	1.1826512	0.0350	1.0697427	0.0369	10.101046	0.6333	6.545693	0.6864
WHH	-0.2012795	0.7188	0.0864004	0.8924	-45.729781	0.0313	8.088175	0.6901
WKF	-0.9858266	0.0785	-0.8963372	0.1294	15.253782	0.4713	13.623847	0.4672
ZWS	0.5894136	0.2921	-0.1687871	0.8805	6.884984	0.7450	0.194688	0.9956

Table S4. Linear regression coefficients of the inbreeding coefficients according to Ballou (F_{a_Bal}), Kalinowski (F_{a_Kal}, F_{a_New}) and Baumung (AHC), within breed on birth year with their *p*-values testing difference to zero for each of the 35 sheep breeds.

Breed	F_{a_Bal}	P-value	F_{a_Kal}	P-value	F_{a_New}	P-value	AHC	P-value
AST	0.0094272727	<0.0001	0.0020090909	<0.0001	0.0005636364	0.2213	0.0136727273	<0.0001
BBS	0.0042545455	<0.0001	0.0002181818	0.1900	-0.0010000000	0.0304	0.0053818182	<0.0001
BDC	0.0009454545	0.1171	-0.0000272727	0.8697	-0.0003636364	0.4297	0.0009545455	0.1895
BLS	0.0060363636	<0.0001	0.0004818182	0.0040	-0.0008727273	0.0587	0.0073727273	<0.0001
BRI	0.0020000000	0.0010	0.0001363636	0.4123	0.0001727273	0.7075	0.0023727273	0.0012
CHA	0.0003636364	0.5460	0.0000818182	0.6227	0.0012000000	0.0095	0.0003636364	0.6168
COF	0.0069727273	<0.0001	0.0003818182	0.0222	-0.0005090909	0.2692	0.0091818182	<0.0001
DOS	0.0070454545	<0.0001	0.0001545455	0.3529	-0.0010909091	0.0183	0.0097545455	<0.0001
GBS	0.0031909091	<0.0001	0.0002363636	0.1557	-0.0020727273	<0.0001	0.0033454545	<0.0001
GGH	0.0068636364	<0.0001	0.0008363636	<0.0001	0.0003545455	0.4413	0.0080363636	<0.0001
IDF	0.0010363636	0.0860	0.0001818182	0.2745	0.0010727273	0.0203	0.0010545455	0.1473
KAM	0.0086636364	<0.0001	0.0004363636	0.0090	-0.0008727273	0.0587	0.0106909091	<0.0001
KST	0.0019727273	0.0012	-0.0003545455	0.0336	-0.0009636364	0.0369	0.0028272727	0.0001
LAC	0.0030363636	<0.0001	0.0005454545	0.0011	0.0010909091	0.0183	0.0030818182	<0.0001
LES	0.0066363636	<0.0001	0.0002909091	0.0809	-0.0003636364	0.4297	0.0089272727	<0.0001
MFS	0.0042636364	<0.0001	0.0002545455	0.1264	-0.0000727273	0.8745	0.0049363636	<0.0001
MLS	0.0048090909	<0.0001	0.0005818182	0.0005	0.0004818182	0.2956	0.0060090909	<0.0001
MLW	0.0029818182	<0.0001	0.0002363636	0.1557	0.0005727273	0.2140	0.0032636364	<0.0001
NOL	0.0045636364	<0.0001	0.0003545455	0.0336	-0.0004090909	0.3744	0.0054909091	<0.0001
OMS	0.0070090909	<0.0001	0.0005090909	0.0024	0.0002818182	0.5405	0.0085909091	<0.0001
OUS	0.0035909091	<0.0001	-0.0005545455	0.0009	-0.0015636364	0.0008	0.0052818182	<0.0001
RHO	0.0041636364	<0.0001	0.0003909091	0.0192	0.0006000000	0.1930	0.0047636364	<0.0001
RPL	0.0080636364	<0.0001	0.0008545455	<0.0001	-0.0005545455	0.2288	0.0114636364	<0.0001
SBS	0.0078090909	<0.0001	0.0014090909	<0.0001	0.0031272727	<0.0001	0.0089454545	<0.0001
SKF	0.0031090909	<0.0001	0.0001090909	0.5118	0.0001727273	0.7075	0.0034818182	<0.0001
SKU	0.0065818182	<0.0001	0.0001454545	0.3819	-0.0009000000	0.0512	0.0088181818	<0.0001
SUF	0.0017454545	0.0040	0.0000454545	0.7845	-0.0001363636	0.7670	0.0019090909	0.0090
SWS	0.0026454545	<0.0001	0.0003000000	0.0719	0.0015090909	0.0011	0.0026454545	0.0003
TEX	0.0003909091	0.5164	-0.0000454545	0.7845	-0.0001636364	0.7222	0.0004909091	0.4994
WAD	0.0067545455	<0.0001	0.0008545455	<0.0001	-0.0009545455	0.0388	0.0100090909	<0.0001
WBS	0.0077545455	<0.0001	0.0011363636	<0.0001	0.0006545455	0.1557	0.0104545455	<0.0001
WGH	0.0130909091	<0.0001	0.0022090909	<0.0001	-0.0000818182	0.8589	0.0189363636	<0.0001
WHH	0.0070909091	<0.0001	0.0005000000	0.0028	-0.0002363636	0.6077	0.0101090909	<0.0001
WKF	0.0046000000	<0.0001	0.0005363636	0.0014	0.0006000000	0.1930	0.0052454545	<0.0001
ZWS	0.0008545455	0.1565	-0.0008454545	<0.0001	-0.0021545455	<0.0001	0.0012545455	0.0850

Table S5. Least-Square (LS) means for the size of the reference population, genealogical estimators number of founders (f), effective number of founders (f_e), effective number of founder genomes (f_g), effective number of ancestors (f_a), and the ratios f_e/f, f_a/f_e, f_g/f_e, losses due to drift and unequal contributions from founders, inbreeding coefficient (F), individual rate of inbreeding (ΔF_i), realized effective population size (N_e), expected ΔF_i in 50 years, degree of deviation (α) of random mating from Hardy–Weinberg proportions, number of progeny per sire and year, effective number of sires and dams by breeding direction groups with their standard errors, and p-values for differences among LS-means.

Group	Size of reference population	P-value for differences among LS-means					
		Merino	Meat	Country	Mountain /Stone	*Heath*	Milk
Merino	95913 ± 15505						
Meat	38753 ± 10151	0.0046					
Country	28979 ± 10151	0.0012	0.5016				
Mountain/Stone	9068 ± 10964	<0.0001	0.0568	0.1934			
Heath	23482 ± 13429	0.0015	0.3721	0.7464	0.4128		
Milk	16456 ± 15506	0.0011	0.2390	0.5048	0.7002	0.7345	
Exotic	4305 ± 12011	<0.0001	0.0370	0.1279	0.7718	0.2962	0.5406
**Group**	**No of founders**	**Merino**	**Meat**	**Country**	**Mountain /Stone**	**Heath**	**Milk**
Merino	5335 ± 803						
Meat	2237 ± 525	0.0032					
Country	1562 ± 525	0.0005	0.3717				
Mountain/Stone	501 ± 568	<0.0001	0.0329	0.1808			
Heath	1713 ± 695	0.0020	0.5518	0.8647	0.1879		
Milk	1108 ± 803	0.0009	0.2490	0.6392	0.5420	0.5737	
Exotic	527 ± 622	<0.0001	0.0448	0.2137	0.9758	0.2141	0.5717
**Group**	**No of effective founders**	**Merino**	**Meat**	**Country**	**Mountain /Stone**	**Heath**	**Milk**
Merino	202 ± 51						
Meat	205 ± 33	0.9612					
Country	107 ± 33	0.1291	0.0466				
Mountain/Stone	55 ± 36	0.0252	0.0047	0.2950			
Heath	107 ± 44	0.1683	0.0864	0.9986	0.3664		
Milk	127 ± 51	0.3061	0.2101	0.7437	0.2551	0.7666	
Exotic	77 ± 39	0.0612	0.0190	0.5604	0.6844	0.6122	0.4394
**Group**	**No of founder genomes**	**Merino**	**Meat**	**Country**	**Mountain /Stone**	**Heath**	**Milk**
Merino	33.0 ± 11.9						
Meat	62.8 ± 7.8	0.0464					
Country	27.8 ± 7.8	0.7172	0.0037				
Mountain/Stone	17.9 ± 8.5	0.3107	0.0006	0.3980			
Heath	22.9 ± 10.4	0.5269	0.0047	0.7083	0.7122		
Milk	29.1 ± 11.9	0.8148	0.0253	0.9320	0.4540	0.7009	
Exotic	26.8 ± 9.3	0.6844	0.0061	0.9360	0.4835	0.7797	0.8847
**Group**	**No of effective ancestors**	**Merino**	**Meat**	**Country**	**Mountain /Stone**	***Heath***	**Milk**
Merino	73.2 ± 22.8						
Meat	108.0 ± 14.9	0.2113					
Country	61.8 ± 14.9	0.6799	0.0370				
Mountain/Stone	33.3 ± 16.1	0.1648	0.0020	0.2056			
Heath	52.4 ± 19.7	0.4975	0.0328	0.7078	0.4599		
Milk	57.0 ± 22.8	0.6202	0.0717	0.8615	0.4037	0.8806	

Exotic	46.9 ± 17.7	0.3694	0.0132	0.5233	0.5762	0.8348	0.7275
**Group**	**f_e/f**	**Merino**	**Meat**	**Country**	**Mountain /Stone**	**Heath**	**Milk**
Merino	0.042 ± 0.034						
Meat	0.111 ± 0.022	0.0974					
Country	0.082 ± 0.022	0.3306	0.3574				
Mountain/Stone	0.123 ± 0.024	0.0583	0.7053	0.2106			
Heath	0.069 ± 0.029	0.5518	0.2582	0.7244	0.1581		
Milk	0.175 ± 0.034	0.0093	0.1232	0.0282	0.2215	0.0242	
Exotic	0.166 ± 0.026	0.0067	0.1141	0.0192	0.2304	0.0185	0.8475
**Group**	**f_a/f_e**	**Merino**	**Meat**	**Country**	**Mountain /Stone**	**Heath**	**Milk**
Merino	0.410 ± 0.075						
Meat	0.552 ± 0.049	0.1218					
Country	0.600 ± 0.049	0.0427	0.5016				
Mountain/Stone	0.645 ± 0.053	0.0157	0.2077	0.5302			
Heath	0.510 ± 0.065	0.3185	0.6077	0.2808	0.1174		
Milk	0.497 ± 0.075	0.4186	0.5374	0.2592	0.1156	0.8912	
Exotic	0.672 ± 0.058	0.0097	0.1254	0.3462	0.7345	0.0729	0.0739
**Group**	**f_g/f_e**	**Merino**	**Meat**	**Country**	**Mountain /Stone**	**Heath**	**Milk**
Merino	0.183 ± 0.056						
Meat	0.329 ± 0.037	0.0382					
Country	0.279 ± 0.037	0.1628	0.3459				
Mountain/Stone	0.377 ± 0.040	0.0087	0.3838	0.0817			
Heath	0.216 ± 0.049	0.6565	0.0752	0.3117	0.0163		
Milk	0.293 ± 0.056	0.1753	0.6000	0.8334	0.2349	0.3085	
Exotic	0.380 ± 0.044	0.0097	0.3781	0.0875	0.9581	0.0182	0.3100
**Group**	**Loss due to drift**	**Merino**	**Meat**	**Country**	**Mountain /Stone**	**Heath**	**Milk**
Merino	0.0127 ± 0.0039						
Meat	0.0078 ± 0.0025	0.2844					
Country	0.0146 ± 0.0025	0.6736	0.0602				
Mountain/Stone	0.0198 ± 0.0027	0.1362	0.0026	0.1661			
Heath	0.0221 ± 0.0033	0.0690	0.0016	0.0771	0.5821		
Milk	0.0129 ± 0.0038	0.9736	0.2677	0.7023	0.1460	0.0741	
Exotic	0.0129 ± 0.0029	0.9720	0.1942	0.6508	0.0913	0.0435	0.9984
**Group**	**Loss due to unequal founders**	**Merino**	**Meat**	**Country**	**Mountain /Stone**	**Heath**	**Milk**
Merino	0.0030 ± 0.0024						
Meat	0.0041 ± 0.0016	0.7042					
Country	0.0059 ± 0.0016	0.3123	0.4118				
Mountain/Stone	0.0120 ± 0.0017	0.0048	0.0019	0.0141			
Heath	0.0061 ± 0.0021	0.3411	0.4549	0.9626	0.0353		
Milk	0.0059 ± 0.0024	0.3985	0.5331	0.9886	0.0473	0.9591	
Exotic	0.0078 ± 0.0019	0.1267	0.1419	0.4591	0.1040	0.5461	0.5432
**Group**	**F**	**Merino**	**Meat**	**Country**	**Mountain /Stone**	***Heath***	**Milk**
Merino	0.0203±0.0077						
Meat	0.0207±0.0050	0.9614					
Country	0.0374±0.0050	0.0721	0.0260				
Mountain/Stone	0.0420±0.0054	0.0282	0.0075	0.5388			
Heath	0.0391±0.0066	0.0738	0.0355	0.8388	0.7386		

Milk	0.0186±0.0077	0.8815	0.8222	0.0501	0.0191	0.0532	
Exotic	0.0301±0.0059	0.3217	0.2399	0.3528	0.1486	0.3180	0.2491
**Group**	**ΔF_i**	**Merino**	**Meat**	**Country**	**Mountain /Stone**	**Heath**	**Milk**
Merino	0.00324±0.00227						
Meat	0.00490±0.00149	0.5457					
Country	0.00758±0.00149	0.1216	0.2140				
Mountain/Stone	0.01319±0.00161	0.0013	0.0008	0.0162			
Heath	0.00627±0.00197	0.3222	0.5835	0.6004	0.0111		
Milk	0.00456±0.00228	0.6840	0.9014	0.2764	0.0044	0.5745	
Exotic	0.00857±0.00176	0.0742	0.1226	0.6695	0.0632	0.3909	0.1740
**Group**	N_e	**Merino**	**Meat**	**Country**	**Mountain /Stone**	**Heath**	**Milk**
Merino	158.3±19.5						
Meat	119.7±12.8	0.1095					
Country	82.8±12.8	0.0031	0.0510				
Mountain/Stone	48.4±13.8	<0.0001	0.0007	0.0785			
Heath	88.5±16.9	0.0117	0.1535	0.7880	0.0768		
Milk	110.6±19.5	0.0955	0.7006	0.2438	0.0148	0.4010	
Exotic	65.8±15.1	0.0008	0.0111	0.3988	0.4030	0.3251	0.0807
**Group**	**Expected ΔF_i in 50 years**	**Merino**	**Meat**	**Country**	**Mountain /Stone**	**Heath**	**Milk**
Merino	0.0399 ± 0.0256						
Meat	0.0631 ± 0.0167	0.4554					
Country	0.0846 ± 0.0167	0.1549	0.3705				
Mountain/Stone	0.1605 ± 0.0181	0.0006	0.0005	0.0046			
Heath	0.0761 ± 0.0221	0.2944	0.6429	0.7608	0.0063		
Milk	0.0712 ± 0.0256	0.3945	0.7921	0.6642	0.0081	0.8864	
Exotic	0.1149 ± 0.0198	0.0281	0.0556	0.2534	0.0998	0.2025	0.1880
**Group**	α	**Merino**	**Meat**	**Country**	**Mountain /Stone**	**Heath**	**Milk**
Merino	0.00753±0.00562						
Meat	0.01235±0.00397	0.4897					
Country	0.02139±0.00368	0.0485	0.1062				
Mountain/Stone	0.02093±0.00397	0.0615	0.1377	0.9340			
Heath	0.01312±0.00435	0.4383	0.8970	0.1579	0.1955		
Milk	0.01073±0.00562	0.6902	0.8159	0.1239	0.1494	0.7395	
Exotic	0.01808±0.00435	0.1489	0.3391	0.5664	0.6319	0.4270	0.3100
**Group**	**Number of progeny per sire and year**	**Merino**	**Meat**	**Country**	**Mountain /Stone**	**Heath**	**Milk**
Merino	68.4 ± 4.5						
Meat	21.6 ± 2.8	<0.0001					
Country	25.5 ± 3.0	<0.0001	0.3492				
Mountain/Stone	16.2 ± 3.2	<0.0001	0.2046	0.0406			
Heath	26.3 ± 3.9	<0.0001	0.3384	0.8697	0.0541		
Milk	20.7 ± 5.5	<0.0001	0.8830	0.4530	0.4803	0.4167	
Exotic	9.9 ± 3.5	<0.0001	0.0137	0.0020	0.1978	0.0041	0.1095
**Group**	**Number of effective sires**	**Merino**	**Meat**	**Country**	**Mountain /Stone**	**Heath**	**Milk**
Merino	58.7 ± 5.0						
Meat	58.7 ± 3.1	0.9961					
Country	44.4 ± 3.3	0.0174	0.0016				

Mountain/Stone	22.7 ± 3.5	<0.0001	<0.0001	<0.0001			
Heath	41.2 ± 4.3	0.0087	0.0011	0.5579	0.0010		
Milk	37.7 ± 6.1	0.0082	0.0023	0.3314	0.0355	0.6343	
Exotic	24.4 ± 3.9	<0.0001	<0.0001	<0.0001	0.7461	0.0041	0.0688

**Group**	**Number of effective dams**	**Merino**	**Meat**	**Country**	**Mountain /Stone**	**Heath**	**Milk**
Merino	4096.9 ± 182.8						
Meat	1467.9 ± 111.9	<0.0001					
Country	1405.4 ± 119.7	<0.0001	0.7031				
Mountain/Stone	385.7 ± 129.3	<0.0001	<0.0001	<0.0001			
Heath	1443.4 ± 158.3	<0.0001	0.8995	0.8483	<0.0001		
Milk	979.9 ± 223.9	<0.0001	0.0520	0.0945	0.0221	0.0918	
Exotic	240.7 ± 141.6	<0.0001	<0.0001	<0.0001	0.4497	<0.0001	0.0055

Assignment of breeds to breed directions:

**Group**	**Breed**
Merino	MFS, MLS, MLW
Meat	CHA, DOS, IDF, SKF, SUF, SWS, TEX, WKF
Country	BLS, BRI, COF, LES, RHO, RPL, WAD
Mountain/Stone	AST, BBS, GBS, KST, SBS, WBS
Heath	GGH, SKU, WGH, WHH
Milk	LAC, OMS
Exotic	BDC, KAM, NOL, OUS, ZWS

Table S6. Least-Square (LS) means for the ancestral inbreeding coefficient according to Ballou (F_{a_Bal}), Kalinowski (F_{a_Kal}) and Baumung (AHC) and new inbreeding coefficient according to Kalinowski (F_{a_New}) and its proportion of inbreeding by breeding direction groups with their standard errors, and *p*-values for differences among LS-means.

**Group**	F_{a_Bal}	**P-value for differences among LS-means**					
		**Merino**	**Meat**	**Country**	**Mountain /Stone**	**Heath**	**Milk**
Merino	0.0620±0.0264						
Meat	0.0511±0.0173	0.7333					
Country	0.1044±0.0173	0.1895	0.0378				
Mountain/Stone	0.0893±0.0187	0.4050	0.1445	0.5577			
Heath	0.1355±0.0229	0.0444	0.0355	0.2875	0.1290		
Milk	0.0373±0.0264	0.5142	0.6650	0.0424	0.1190	0.0089	
Exotic	0.0818±0.0204	0.5580	0.2619	0.4051	0.7875	0.0909	0.1937
**Group**	F_{a_Kal}	**Merino**	**Meat**	**Country**	**Mountain /Stone**	**Heath**	**Milk**
Merino	0.00433±0.00425						
Meat	0.00414±0.00278	0.9703					
Country	0.01214±0.00278	0.1353	0.0515				
Mountain/Stone	0.01233±0.00300	0.1354	0.0552	0.9632			
Heath	0.01525±0.00368	0.0622	0.0229	0.5061	0.5442		
Milk	0.0033±0.004249	0.8690	0.8745	0.0938	0.0947	0.0430	
Exotic	0.00840±0.00329	0.4556	0.3316	0.3924	0.3849	0.1762	0.3539
**Group**	F_{a_New}	**Merino**	**Meat**	**Country**	**Mountain /Stone**	**Heath**	**Milk**
Merino	0.01600 ± 0.00398						
Meat	0.01671 ± 0.00260	0.8816					
Country	0.02629 ± 0.00260	0.0392	0.0147				
Mountain/Stone	0.02967 ± 0.00281	0.0090	0.0022	0.3852			
Heath	0.02375 ± 0.00344	0.1519	0.1144	0.5617	0.1941		
Milk	0.01567 ± 0.00398	0.9532	0.8272	0.0337	0.0077	0.1357	
Exotic	0.02160 ± 0.00308	0.2751	0.2359	0.2552	0.0633	0.6454	0.2482
**Group**	**Proportion of** F_{a_New}	**Merino**	**Meat**	**Country**	**Mountain /Stone**	**Heath**	**Milk**
Merino	0.812 ± 0.069						
Meat	0.860 ± 0.045	0.5688					
Country	0.738 ± 0.045	0.3708	0.0653				
Mountain/Stone	0.722 ± 0.049	0.2902	0.0463	0.8104			
Heath	0.629 ± 0.059	0.0538	0.0045	0.1576	0.2397		
Milk	0.879 ± 0.069	0.4971	0.8141	0.0958	0.0716	0.0103	
Exotic	0.761 ± 0.053	0.5588	0.1674	0.7410	0.5898	0.1105	0.1844
**Group**	**AHC**	**Merino**	**Meat**	**Country**	**Mountain /Stone**	**Heath**	**Milk**
Merino	0.06933±0.03446						
Meat	0.05742±0.02256	0.7747					
Country	0.12171±0.02256	0.2139	0.0536				
Mountain/Stone	0.10500±0.02436	0.4052	0.1631	0.6187			
Heath	0.16100±0.02984	0.0541	0.0099	0.3027	0.1572		
Milk	0.04133±0.03446	0.5702	0.6989	0.0611	0.1426	0.0139	
Exotic	0.10040± 0.02669	0.4819	0.2291	0.5469	0.8996	0.1414	0.1862

Assignment of breeds to breed directions:

Group	Breed
Merino	MFS, MLS, MLW
Meat	CHA, DOS, IDF, SKF, SUF, SWS, TEX, WKF
Country	BLS, BRI, COF, LES, RHO, RPL, WAD
Mountain/Stone	AST, BBS, GBS, KST, SBS, WBS
Heath	GGH, SKU, WGH, WHH
Milk	LAC, OMS
Exotic	BDC, KAM, NOL, OUS, ZWS

Table S7. Number of dams (No_dams) and effective number of dams (Effective_dams) for each birth year from 2010 to 2020 with their standard deviations (SD) for each of the 35 sheep breeds.

Breed	Mean (No_dams)	Min (No_dams)	Max (No_dams)	SD (mean No_dams)	Mean (Effective _dams)	Min (Effective _dams)	Max (Effective _dams)	SD (mean Effective_ dams)
AST	444.091	277	587	107.897	369.971	225.518	495.061	96.087
BBS	661.273	595	699	31.471	535.350	472.204	562.307	26.952
BDC	165.727	144	194	13.093	136.939	115.497	165.024	14.326
BLS	1742.273	1376	2002	227.525	1495.256	1193.530	1713.536	187.780
BRI	370.909	308	426	36.503	304.210	253.526	348.023	30.137
CHA	441.727	279	727	116.506	385.298	237.772	636.028	103.735
COF	2322.091	1882	2763	305.660	1962.299	1572.063	2354.509	272.995
DOS	1458.636	872	1955	328.512	1184.069	689.635	1622.352	279.428
GBS	164.182	78	243	62.740	134.852	61.357	199.254	53.318
GGH	2979.455	2324	3400	415.697	2637.074	2068.887	3001.615	363.576
IDF	451.273	357	578	72.875	378.945	300.755	492.084	66.296
KAM	181.000	78	264	64.271	158.449	68.504	230.890	56.391
KST	420.455	251	635	123.186	366.935	211.240	563.590	112.085
LAC	212.545	88	317	84.718	183.859	78.373	274.994	73.372
LES	2022.545	1476	2487	291.552	1735.992	1285.448	2103.036	237.992
MFS	4655.182	3358	5323	556.907	3949.151	2856.223	4490.338	458.165
MLS	8262.727	6080	9788	1055.943	6870.422	5121.823	8147.578	856.561
MLW	1781.545	1195	2232	359.665	1471.050	1019.260	1853.580	280.113
NOL	437.636	272	507	71.697	383.625	240.447	441.720	61.561
OMS	2051.273	1726	2271	173.392	1776.035	1493.843	1979.645	155.121
OUS	438.727	368	512	55.254	429.639	358.822	509.031	59.503
RHO	2729.364	2170	3292	394.919	2262.116	1809.736	2712.228	320.119
RPL	1818.000	1152	2318	403.809	1613.333	1038.586	2033.847	345.458
SBS	103.000	68	124	19.173	83.742	53.702	99.758	15.837
SKF	5328.364	3715	5742	558.233	4229.367	1837.026	4871.272	909.691
SKU	1036.091	583	1378	218.083	907.290	521.388	1210.947	190.383
SUF	2792.364	2351	3055	207.073	2384.935	2022.827	2616.254	185.159
SWS	134.364	100	161	19.382	118.246	88.360	140.174	16.333
TEX	2352.182	1752	2836	303.322	2085.110	1567.501	2490.501	260.149
WAD	556.636	399	693	83.885	464.919	333.942	578.202	67.829
WBS	1003.091	884	1192	118.973	823.622	717.978	982.998	100.399
WGH	868.364	625	1069	137.002	767.756	547.798	945.009	121.281
WHH	1654.909	1081	1962	316.006	1461.652	952.350	1730.780	283.370
WKF	1137.000	1047	1242	62.844	977.556	913.852	1059.076	54.187
ZWS	112.364	73	169	27.707	94.679	57.525	147.366	25.923

Table S8. Number of sires (No_sires) and effective number of sires (Effective_sires) for each birth year from 2010 to 2020 with their standard deviations (SD) for each of the 35 sheep breeds.

Breed	Mean (No_sires)	Min (No_sires)	Max (No_sires)	SD (mean No_sires)	Mean (Effective _sires)	Min (Effective _sires)	Max (Effective_ sires)	SD (mean Effective_sires)
AST	49.636	37	58	8.213	25.575	15.067	32.291	6.086
BBS	67.909	55	96	11.211	29.736	22.249	38.416	5.642
BDC	33.455	14	48	10.577	12.778	8.771	18.006	2.256
BLS	87.273	77	100	7.016	32.427	18.932	41.616	5.930
BRI	44.909	39	53	4.437	18.720	16.713	21.040	1.527
CHA	69.727	31	94	21.546	14.286	7.861	19.321	3.888
COF	179.273	158	199	14.423	80.212	59.954	100.391	15.177
DOS	153.364	138	174	11.387	57.967	43.480	72.564	9.286
GBS	25.455	8	37	9.791	11.104	2.469	17.067	5.463
GGH	105.636	83	119	11.578	55.836	39.538	61.915	6.577
IDF	30.000	23	40	4.940	11.672	9.797	16.044	1.776
KAM	22.273	14	27	4.541	12.190	7.179	19.174	3.772
KST	60.182	41	70	9.765	33.363	25.994	44.909	6.144
LAC	18.091	7	40	12.446	6.486	2.764	10.629	2.254
LES	80.636	66	93	7.865	37.738	25.730	50.912	8.344
MFS	113.727	84	133	15.749	55.283	39.703	71.966	9.779
MLS	220.545	171	257	22.460	102.225	75.294	112.083	11.220
MLW	41.909	28	55	6.804	18.560	11.891	28.002	4.711
NOL	46.545	32	60	10.492	22.786	16.948	32.134	6.027
OMS	166.273	139	179	12.142	68.826	63.706	76.201	4.376
OUS	105.364	95	116	6.712	64.106	51.850	79.435	8.753
RHO	138.727	121	158	11.136	59.755	47.359	75.045	7.753
RPL	123.364	91	150	17.727	56.831	43.692	69.528	8.378
SBS	14.364	11	20	2.335	6.861	5.180	8.973	1.226
SKF	232.182	195	247	13.884	107.246	81.491	115.904	9.918
SKU	100.636	67	134	19.007	47.219	37.117	56.580	6.284
SUF	263.818	193	325	47.027	97.702	79.857	110.198	8.622
SWS	14.909	12	20	2.386	6.638	4.716	10.385	1.882
TEX	239.000	166	293	41.701	123.099	95.650	140.323	14.932
WAD	49.727	40	59	4.628	25.197	17.885	32.713	4.839
WBS	66.364	56	85	9.069	29.726	24.611	41.190	5.136
WGH	52.636	41	64	7.201	31.063	17.416	38.153	5.654
WHH	63.818	55	70	5.363	30.791	22.776	38.579	4.489
WKF	91.818	74	109	10.515	50.679	46.259	61.842	4.515
ZWS	25.182	19	37	5.135	10.276	6.860	16.098	2.623
