# Supplementary material for: Genetic Diversity and Trends of Ancestral and New Inbreeding in German Sheep Breeds by Pedigree Data
Source: Animals (Basel). 2023 Feb 10;13(4):623. doi: 10.3390/ani13040623 (PMC9951766; doi:10.3390/ani13040623)
Supplement: Supplementary file 1 [file animals-13-00623-s001.zip › Table S10-S11.pdf]

Table S10. Correlations of measures of inbreeding of animals with inbreeding measures of their sires and dams for each of the 35 sheep breeds.

Breed	$F_{a\_New-Animal-Sire}$	$F_{a\_New-Animal-Dam}$	$F_{a\_Kal-Animal-Sire}$	$F_{a\_Kal-Animal-Dam}$
**AST**	0.13837	0.06601	0.49270	0.55178
**BBS**	0.07419	0.07710	0.53921	0.22777
**BDC**	0.05125	-0.01279	0.4645	0.0289
**BLS**	-0.02438	0.07694	0.27409	0.40731
**BRI**	-0.00660	0.03330	0.18763	0.14847
**CHA**	0.03491	0.00000	0.00294	0.02139
**COF**	0.00818	0.15090	0.24521	0.39312
**DOS**	0.08496	0.06601	0.52112	0.51472
**GBS**	-0.07592	0.02063	0.19462	0.33778
**GGH**	-0.02855	0.13541	0.38357	0.51675
**IDF**	0.02414	0.02108	0.19352	0.08657
**KAM**	0.05523	0.02372	0.31034	0.24844
**KST**	0.15901	0.10669	0.48694	0.40385
**LAC**	0.25131	0.08099	0.30219	0.34816
**LES**	-0.02985	0.02379	0.45041	0.49458
**MFS**	0.01292	0.07860	0.21648	0.30354
**MLS**	0.01859	0.06570	0.31097	0.45202
**MLW**	0.06913	0.02958	0.25778	0.22866
**NOL**	0.20894	0.14218	0.27668	0.39773
**OMS**	0.06469	0.10418	0.30886	0.44932
**OUS**	0.07861	0.07974	0.41796	0.36235
**RHO**	0.01701	0.05930	0.26803	0.34067
**RPL**	0.04758	0.03049	0.39663	0.45521
**SBS**	0.06554	0.06710	0.27460	0.27921
**SKF**	0.01947	0.00670	0.24631	0.22119
**SKU**	-0.00137	0.07501	0.35124	0.44240
**SUF**	0.02485	0.02730	0.22783	0.33802
**SWS**	0.09310	0.03247	0.21757	0.09802
**TEX**	0.04753	0.03342	0.31418	0.23824
**WAD**	0.07382	0.05855	0.70530	0.70605
**WBS**	0.07507	0.09170	0.35525	0.49978
**WGH**	0.03117	0.14312	0.36861	0.63548
**WHH**	0.05500	0.11224	0.21255	0.48343
**WKF**	0.08006	0.05246	0.28569	0.35815
**ZWS**	0.15553	0.23412	0.35603	0.50122

Table S11. Correlations of measures of inbreeding among sires and dams for each of the 35 sheep breeds.

Breed	$F_{\text{-Sire-Dam}}$	$F_{a\_\text{New-Sire-Dam}}$	$F_{a\_\text{Kal-Sire-Dam}}$	$F_{a\_\text{Bal-Sire-Dam}}$
**AST**	0.04049	0.04736	0.07234	0.12100
**BBS**	0.02391	0.01999	0.05307	0.17651
**BDC**	0.03202	0.03268	-0.00265	0.03613
**BLS**	-0.00224	0.00419	0.03653	0.15586
**BRI**	-0.01539	-0.01620	-0.01456	0.08888
**CHA**	-0.00929	-0.00914	0.00000	-0.00574
**COF**	0.00959	0.00838	0.02918	0.14127
**DOS**	0.09852	0.08504	0.10076	0.22255
**GBS**	0.07162	0.08112	0.03217	0.21770
**GGH**	0.04272	0.01803	0.18805	0.34799
**IDF**	0.00286	0.00516	-0.00494	-0.02863
**KAM**	0.08806	0.08326	0.07331	0.05273
**KST**	0.06659	0.07261	0.04322	0.07150
**LAC**	0.08845	0.08486	0.03965	0.24088
**LES**	0.00455	-0.01583	0.19333	0.49472
**MFS**	0.02503	0.02327	0.03423	0.12634
**MLS**	0.03559	0.01543	0.09768	0.13896
**MLW**	0.01186	0.01268	0.03485	0.12613
**NOL**	0.03327	0.01769	0.10528	0.17370
**OMS**	0.09341	0.06926	0.15764	0.36024
**OUS**	0.05483	0.01202	0.14523	0.31077
**RHO**	0.09116	0.08543	0.10313	0.28865
**RPL**	0.09177	0.03336	0.21152	0.38838
**SBS**	-0.03929	-0.03982	-0.03450	0.10966
**SKF**	0.01079	0.00445	0.05290	0.09017
**SKU**	0.08389	0.04167	0.15260	0.31496
**SUF**	0.03155	0.01960	0.07753	0.14248
**SWS**	-0.04932	-0.04818	-0.00208	-0.05506
**TEX**	0.02971	0.01021	0.03925	0.18644
**WAD**	0.22830	0.01404	0.49712	0.38190
**WBS**	0.10305	0.09025	0.17339	0.23167
**WGH**	0.16332	0.10158	0.23037	0.26595
**WHH**	-0.03605	-0.02564	-0.01113	0.11426
**WKF**	-0.00211	-0.00644	0.02882	0.19529
**ZWS**	0.16002	0.09930	0.11352	0.24013
