# Supplementary material for: Genetic Diversity and Trends of Ancestral and New Inbreeding in German Sheep Breeds by Pedigree Data
Source: Animals (Basel). 2023 Feb 10;13(4):623. doi: 10.3390/ani13040623 (PMC9951766; doi:10.3390/ani13040623)
Supplement: Supplementary file 1 [file animals-13-00623-s001.zip › Table S12-Autochthon imported sheep breeds.pdf]

Table S12. Autochthonous and imported sheep breeds in Germany with their risk levels, sustainable developmental goal (SDG) risk status and their local endangerment status.

Code	Breed	Risk level	SDG local risk status	Detailed local risk status	Country of origin
AST	Alpine Steinschaf	at risk	at risk	endangered	Germany
BBS	Brown Mountain	global not at risk, national at risk	at risk	endangered	Germany
BDC	Berrichon du Cher	global not at risk, national at risk	at risk	critical	France
BLS	Bentheim	at risk	at risk	endangered	Germany
BRI	Carinthian	global not at risk, national at risk	at risk	endangered	Germany
CHA	Charollais	global not at risk, national at risk	at risk	endangered	France
COF	Coburg	global at risk, national at risk	at risk	vulnerable	Germany
DOS	Dorper	global not at risk, national at risk	at risk	endangered	South Africa
GBS	Baraka	global at risk, national at risk	at risk	critical	Germany
GGH	German Grey Heath	national at risk	at risk	endangered	Germany
IDF	Ile-de-France	national at risk	at risk	endangered	France
KAM	Kamerun	national at risk	at risk	critical	West Africa

KST	Krainer Steinschaf	global not at risk,<br>national at risk	at risk	endangered	Germany
LAC	Lacaune	global not at risk,<br>national at risk	at risk	critical	France
LES	Leine	global at risk,<br>national at risk	at risk	endangered	Germany
MFS	German Mutton Merino	global not at risk,<br>national at risk	at risk	vulnerable	Germany
MLS	German Merino	global not at risk,<br>national not at risk	not at risk		Germany
MLW	Merino Longwool	national at risk	at risk	endangered	Germany
NOL	Nolana	national at risk	at risk	endangered	Germany
OMS	East Friesian	global not at risk,<br>national at risk	at risk	endangered	Germany
OUS	Ouessant	global not at risk,<br>national at risk	at risk	endangered	France
RHO	Rhön	national at risk	at risk	vulnerable	Germany
RPL	Pomeranian Coarsewool	national at risk	at risk	endangered	Germany
SBS	Black Mountain	national at risk	at risk	critical	Germany
SKF	German Blackhead Mutton	global not at risk,<br>national at risk	at risk	vulnerable	Germany
SKU	Skudde	global at risk,<br>national at risk	at risk	endangered	Germany

SUF	Suffolk	global not at risk, national at risk	at risk	vulnerable	United Kingdom
SWS	Swifter	global at risk, national at risk	at risk	critical	Holland
TEX	Texel	global not at risk, national at risk	at risk	endangered	Holland
WAD	Wald	global at risk, national at risk	at risk	endangered	Germany
WBS	White Mountain	global not at risk, national at risk	at risk	endangered	Germany
WGH	German White Heath	national at risk	at risk	endangered	Germany
WHH	White Polled Heath	national at risk	at risk	endangered	Germany
WKF	German Whitehead Mutton	national at risk	at risk	endangered	Germany
ZWS	Zwartbles	global not at risk, national at risk	at risk	critical	Holland
